# Bioequivalence evaluation and food effect assessment of Lisinopril/amlodipine tablets in healthy Chinese subjects under fasting and fed conditions

**DOI:** 10.1186/s40360-022-00590-6

**Published:** 2022-07-07

**Authors:** Ting Li, Yan-ping Liu, Shu-qin Liu, Ping Shi, Xin Jiang, Ye Tao, Xiao-meng Gao, Ya-ping Ma, Yu Cao

**Affiliations:** Phase I Clinical Research Center, The Affiliated Hospital of Qingdao University, Qingdao, Shandong China

**Keywords:** Lisinopril, Amlodipine, Bioequivalence, Pharmacokinetic, Food effect

## Abstract

**Purpose:**

The combination of lisinopril and amlodipine has a marked additional effect on blood pressure and fewer side effects than individual monotherapy. This study was conducted to compare the pharmacokinetic parameters and evaluate the bioequivalence between two Lisinopril/amlodipine tablets in healthy Chinese subjects.

**Methods:**

A single center, randomized, open-label, single-dose, two-period crossover bioequivalence study was designed in healthy Chinese subjects under both fasting and fed conditions. Blood samples were collected before drug administration and at 1, 2, 3, 4, 5, 6, 7, 8, 9, 10, 11, 12, 13, 24, 36, 48, 72, 96, 144, 168 h after administration. Liquid chromatography-tandem mass spectrometry (LC–MS/MS) was applied to determine the plasma concentration of lisinopril and amlodipine. Maximum concentration (C_max_) and area under the concentration–time curve (AUC) were used to evaluate bioequivalence. Adverse events were recorded.

**Results:**

Ninety-two healthy subjects were enrolled, and 75 completed the study. The 90% confidence intervals (CIs) of the ratio of geometric means (GMRs) of C_max_, AUC_0-t,_ and AUC_0-∞_ of lisinopril and amlodipine under both fasting and fed conditions fell within the conventional bioequivalence criteria of 0.80–1.25. A high-fat meal appeared to decrease the C_max_ and AUC of lisinopril. No severe adverse events were observed.

**Conclusion:**

The trial demonstrated that the test and the reference lisinopril/amlodipine tablets were bioequivalent and well tolerated in Chinese people under fasting and fed conditions.

**Trial registration:**

Clinical Trails.gov identifier, NCT04885660 (retrospectively registered in 13/05/ 2021).

**Supplementary Information:**

The online version contains supplementary material available at 10.1186/s40360-022-00590-6.

## Introduction

Hypertension is an independent and major risk factor for cardiovascular diseases, and a lowering blood pressure (BP) substantially reduces premature morbidity and mortality [1]. The 2019 annual report on cardiovascular health and diseases in China indicated that, the number of Chinese residents with hypertension has reached 245 million [2]. However, only 45.8% of the patients are treated, and the control of hypertension was 16.8% [3]. Five classes of anti-hypertensive drugs, including calcium channel blockers (CCB), angiotensin-converting enzyme inhibitors (ACEI), angiotensin receptor blockers (ARB), diuretics, β-blockers, and fixed-ratio preparations composed of the above drugs, are often recommended. High risk group of patients with BP ≥ 160/100 mmHg and 20/10 mmHg higher than that of the target BP, or patients who receive mono-therapy and do not achieve the goal BP should be treated with combination therapy [4]. One of the preferred specific drug regimens is ACEI/CCB, as the most common adverse effects of CCBs, peripheral edema and tachycardia, are partially neutralized by ACEI [5].

Lisinopril, an ACEI, can decrease peripheral vascular resistance and reduce blood pressure, preload, and after load, without changes in heart rate [6]. Lisinopril is the only ACE inhibitor that exhibits a linear dose–response curve [7]. The antihypertensive effect of lisinopril usually appears within 1 h after oral administration, and peaks at about 6 h. Bioavailability of lisinopril is about 20 − 28%, and its cumulative effective half-life after multiple administration is about 12 h [8–10]. Lisinopril does not bind to other plasma proteins other than ACE, and it is excreted from the urine in its original form without undergoing metabolic transformation [11].

Amlodipine, a dihydropyridine-based CCB, inhibits the transmembrane influx of calciumions into vascular smooth muscle and is indicated for the management of stable angina and hypertension. Amlodipine is almost completely absorbed and is converted to inactive metabolites by CYP3A4 in liver [12]. After single oral administration, amlodipine reaches at C_max_ within 6.0–8.0 h and has a terminal elimination half-life of 40–50 h, with high oral bioavailability of 60%–65% [13, 14].

The combination of lisinopril and amlodipine, two classes of long-acting drugs, has a marked additional effect on blood pressure and fewer side effects than individual monotherapy [15, 16]. Though many pharmacokinetics (PK) studies for lisinopril and amlodipine as a single pill have been reported, very few were focused on the combination. Lisinopril/amlodipine tablets (Lisonorm®, 10 mg/ 5 mg) has been developed by Gedeon Richter Ltd and approved in multiple countries in the European Union, but not yet in China. The aim of this study was to compare the PK characteristics and evaluate the bioequivalence between Lisonorm and a newly developed Lisinopril/amlodipine tablets in healthy Chinese subjects.

## Material and methods

### Formulations

The test product of Lisinopril/amlodipine tablets (batch no.:180101; expiration date: December 2019) was produced by Sichuan MEIDAKANG Pharmaceutical Co. Ltd (Sichuan Province,China), and developed by Sichuan Sunrise Biopharm Co. Ltd (Sichuan Province, China).

The reference product of Lisonorm® (batch no.:T79030A; expiration date: September 2019) was produced by Gedeon Richter Ltd (Hungary).

### Subjects

Healthy volunteers that meet the inclusion criteria and not the exclusion criteria were enrolled in the study after the clinical and laboratory examinations. The inclusion criteria included as follows: 1) healthy male and female aged over 18 years; 2) the Body Mass Index is in the range of 19.0 to 26.0 kg/m^2^ (both inclusive), and males with minimum of 50 kg weight, females with minimum of 45 kg weight; 3) subjects have no clinically significant abnormalities, including vital signs (BP should not be less than 90/60 mmHg and the heart rate ranges from 50–100 beats per minute), physical examinations, laboratory tests, and ECG as determined by clinical examination; 4) agree to follow approved birth control methods.

Subjects were excluded if any of the following conditions were present: 1) allergic diathesis or hypersensitivity to investigational products; 2) history or presence of significant cardiovascular, urogenital, pulmonary, hepatic, renal, gastrointestinal, endocrine, immunological, dermatological, neurological or psychiatric disease or disorder, or other medical history affecting drug absorption; 3) use of any drugs or herbal medicine within 14 days; 4) smoking more than five cigarettes a day, abuse of alcohol or drugs, drinking too much tea, coffee or caffeinated drinks (more than 8 cups a day, 250 ml/cup); 5) donation or loss of blood or plasma > 400 mL in the past 3 months; 6) consumption of any beverages or food containing caffeine or products rich in grapefruit, such as coffee, tea and chocolate, etc., within 48 h prior to receiving study drug.

### Ethic

The bioequivalence study has been registered on ClinicalTrials.gov (No.: NCT04885660, retrospectively registered in 13/05/2021) and been approved by the Medical Ethics Committee of the Affiliated Hospital of Qingdao University on August 01, 2018 (No.: QYFYEC 2018–055–01). The study was performed at Phase I Clinical Research Center of the Affiliated Hospital of Qingdao University and was conducted in accordance with the Declaration of Helsinki, Good Clinical Practice (GCP) and applicable laws and regulations of China National Medical Products Administration (NMPA).Written informed consent was obtained from all subjects before their participation in the study.

### Study design

**Fig. 1:**
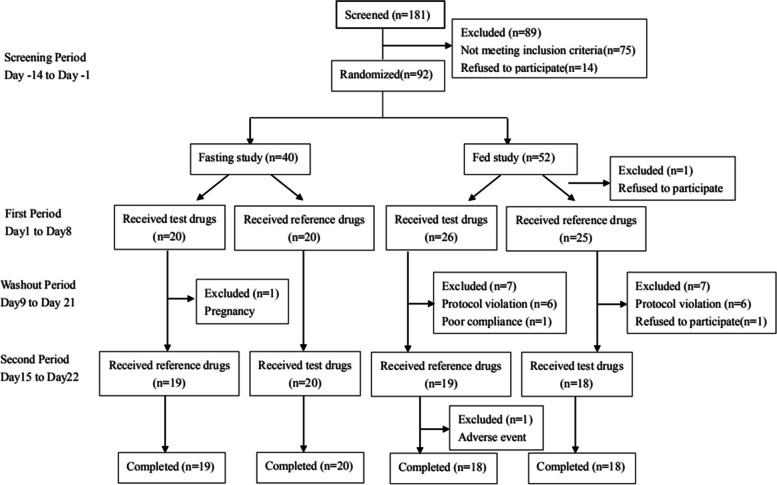
The study flow diagram

This was a single center, randomized, open-label, single-dose, two-period crossover bioequivalence study in both fasting and fed conditions. According to pre-test and previous studies, the intra-subject coefficient of variation (CV), which was calculated by the standard deviation (SD) according to the formula: CV = [exp (SD^2^)-1]^1/2^, of lisinopril ranged from 20%-28% [8–10], while amlodipine ranged from 10–18% [13, 14]. Under the condition that α = 0.05, statistical test efficiency 1-β = 0.9, intra-subject CV was 0.28, equivalent lower limit was 0.80, upper limit was1.25 and actual ratio was 1.05, 35 samples were needed. Considering the possibility of shedding, 40 subjects were planned to be selected in each fasting and fed bioequivalence study. If the number of finally assessable subjects is less than 35, the substitute subjects can replace the subjects falling off. All eligible subjects were assigned according to the random table generated by SAS 9.4 and took the test (T) and reference(R) drugs respectively with 240 ml water after an overnight fast of at least 10 h (fasting study) or after the high-fat breakfast within half an hour before dosing (fed study). The high-fat breakfast contained about 900 kcal calories, and consisted of three pork buns with cabbage, spinach mixed with Yuba, and millet gruel. Subjects were forbidden to drink water within 1 h before and after taking the drug, and the lunch and dinner were provided at 4 h and 8 h respectively post-drug administration. No other food and beverage intake was permitted except the provided diets. A washout of 14 days was set between the two administrations, according to the half-life recorded in original drug instructions. 4 ml venous blood samples were collected before drug administration and at 1,2,3,4,5,6,7,8,9,10,11,12,13,24,36,48,72,96,144,168 h after administration. The samples were centrifuged at 1700gear per minute for 10 min at 4℃ to separate the plasma, which was divided into two aliquots (drug monitoring at least 800ul and backup) and stored at - 80℃until analysis. The study flow chart is presented in Fig. 1.

### Bioanalytical assay

Plasma concentrations of lisinopril and amlodipine were measured by an established and validated liquid chromatography-tandem mass spectrometry (LC–MS/MS) method at Suzhou Haike Pharmaceutical Technology Co., Ltd (Suzhou, Jiangsu Province, China). For the analysis of lisinopril, plasma samples were pretreated by liquid liquid extraction with isopropanol: ethyl acetate(1:2,V/V); LSN-d5 was used as internal standard; 5 mM ammonium acetate aqueous solution, 0.01% formic acid and methanol were used as mobile phase; chromatographic separation was performed on Atlantis-dC18 column(Waters, Massachusetts, USA) and the analytes were detected using Triple Quad TM 5500 tandem mass spectrometer(Sciex, Canada) in positive ion mode, with ion spray in multiple reaction monitoring mode. The lower limit of quantification (LLOQ) was 0.500 ng/mL and the assay dynamic range was 0.500-100 ng/mL. The intra- and inter-day accuracy ranges were 93.8–108.5% and 98.7–104.6%, while 93.8–99.9% and 96.5% for LLOQ, respectively. The intra- and inter-day precision coefficient of variation (CV) % were < 4.3% and < 5.6%, while 5.5% and 5.2% for LLOQ, respectively. The analytes in matrix were stable when stored at -20℃ for 26 days, at -80℃ for 169 days and after four freeze–thaw cycles.

Amlodipine plasma concentrations were determined using a liquid chromatography unit (Shimadzu, LC-30AD, Japan) and a mass spectrometer (Sciex, Triple Quad TM 6500 plus, Canada). Under multiple reaction monitoring, LC–MS/MS system adopts positive ionization mode. For the analysis of amlodipine, the LLOQ was 0.050 ng/mL and the assay dynamic range was 0.050–10.0 ng/mL. The intra- and inter-day accuracy ranges were 99.4–105.5% and 102.0–103.8%, while 104.7–107.6% and 105.8% for LLOQ, respectively. The intra- and inter-day precision coefficient of variation (CV) % were < 6.0% and < 5.3%, while 8.1% and 5.2% for LLOQ, respectively. The analytes in matrix were stable when stored at -20℃ for 91 days, at -80℃ for 207 days and after four freeze–thaw cycles.

Data collection and analysis was performed with Analyst 1.6.3 software (Sciex, Canada) and Watson LIMS (Thermo, USA). Calibration curves were constructed using linear regression equation obtained by the weighted (W = 1/X2) least square method fitting for both analytes. Quantitation of quality control and clinical samples were also performed by the Analyst software using the same mathematical algorithm as that used in the calibration of standard curves.

### Pharmacokinetic analysis

PK parameters for lisinopril and amlodipine in plasma were estimated by a non-compartmental model (NCA) using Phoenix WinNonlin version 7.0 software (Pharsight Corporation, St Louis, MO, USA). For the purpose of bioequivalence analysis, the maximum observed concentration (C_max_), the area under the plasma concentration–time curve from time 0 to the last measured time point (AUC_0-t_), and the area under the plasma concentration–time curve from time 0 to infinity (AUC_0-∞_) were considered as primary PK parameters. The secondary PK parameters were the observed time to C_max_ (T_max_) and the apparent terminal half-life (T_1/2_). C_max_ and T_max_ were the factually measured data and AUC_0-t_ was calculated using the linear and logarithmic trapezoidal methods. AUC_0-∞_ was calculated according to the following formula: AUC_0-∞_ = AUC0-t + C_last_/λ_z_ (C_last_ is the last measurable concentration and is the first order rate constant of terminal elimination determined from a linear regression line after logarithmic transformation at the end of concentration time curve. λ_z_ is the slope calculated by linear regression after logarithmic conversion at the end of the concentration–time curve). T_1/2_ was calculated to be ln2/λ.

### Safety assessment

The safety was evaluated by monitoring vital signs, physical examination, laboratory tests, electrocardiogram (ECG) and adverse events (AEs) collected after dosing throughout the study. Vital signs, including body temperature, blood pressure (BP) and heart rate, were measured at screening, before drug administration and at 2, 4, 6, 8, 12, 24, 36, 48, 72, 96, 144, 168 h after administration. Routine laboratory tests (hematology, urinalysis, serum chemistry and pregnancy test for females) and 12-lead ECG were conducted at screening and before removal from the study. The AEs, including all subjective symptoms reported by subjects and objective signs observed by clinical investigators, were recorded and assessed for their severity and the correlation with research drugs.

### Statistical analysis

Statistical analysis was performed by SAS 9.4. Demographic characteristics, safety parameters and pharmacokinetic data were summarized using descriptive statistics, the results were presented as the mean ± SD and the differences between groups were determined by two one-sided tests. The probability value (P) less than 0.05 was considered statistically significant. AUC and C_max_ were logarithmically transformed and analyzed by linear mixed effect model. Sequence, period and formulation were fixed effects, and subject within sequence was included as a random effect. Analysis of variance (ANOVA) of cross-over design was performed on the log-transformed variables. The geometric mean ratios (GMRs) of the primary pharmacokinetic parameters and their 90% confidence intervals (CIs) were calculated, and the test formulation was judged as bioequivalence if it fell within the equivalent range (80–125%). Bioequivalence was assessed separately in both the fasting and fed groups.

## Results

### Subject characteristics

A total of 181 subjects were screened for inclusion; 92 healthy subjects (40 fasting group and 52 fed group) were randomized into each of the study group, and 75 subjects (39 of fasting group and 36 of fed group) completed the study. 1 subject in fasting group withdrew because of pregnancy before admission in second period. 16 subjects in fed group fell off, among which 12 subjects dropped out as a result of failing to finish the high-fat breakfast within 30 min, and another 4 subjects dropped out due to poor compliance, voluntary withdrawal and AE of tonsillitis. There were 12 eligible subjects in the waiting list and these subjects were all enrolled into the fed group to ensure sufficient pharmacokinetic data.

Data from the subjects who received a study drug at least once were used for safety assessment and subjects who completed the study were included in the PK analysis. The baseline demographic characteristics of subjects showed no statistical difference between the sequence groups (Table 1).

**Table 1:** Baseline demographic characteristics

Sequence	Fasting study (*n* = 40)	Fed study (*n* = 51)	Total	P
Age(years)	28.1 ± 7.7	31.0 ± 9.0	29.7 ± 8.5	0.11
Male/Female	30/10	38/13	62/23	0.96
Height(cm)	169.0 ± 9.1	169.7 ± 7.5	169.4 ± 8.2	0.69
Weight(kg)	63.4 ± 8.9	64.4 ± 8.2	64.0 ± 8.5	0.58
BMI(kg/m^2^)	22.1 ± 1.9	22.3 ± 2.0	22.2 ± 1.9	0.63
PR(beats/min)	74.7 ± 9.7	75.1 ± 11.5	74.9 ± 10.7	0.86
SBP(mmHg)	121.2 ± 10.9	121.7 ± 12.2	121.5 ± 11.6	0.84
DBP(mmHg)	72.5 ± 7.5	73.0 ± 7.7	72.7 ± 7.6	0.76

*Notes*: T, test formulation, compound lisinopril tablet (lisinopril /amlodipine besylate 10 mg /5 mg); R, reference formulation, Lisonorm® (lisinopril /amlodipine besylate 10 mg /5 mg); *P*-values were determined by independent t test

*Abbreviation*: *BMI* Body mass index, *PR* Pulse rate, *SBP* Systolic blood pressure, *DBP* Diastolic blood pressure

### Pharmacokinetics

**Fig. 2:**
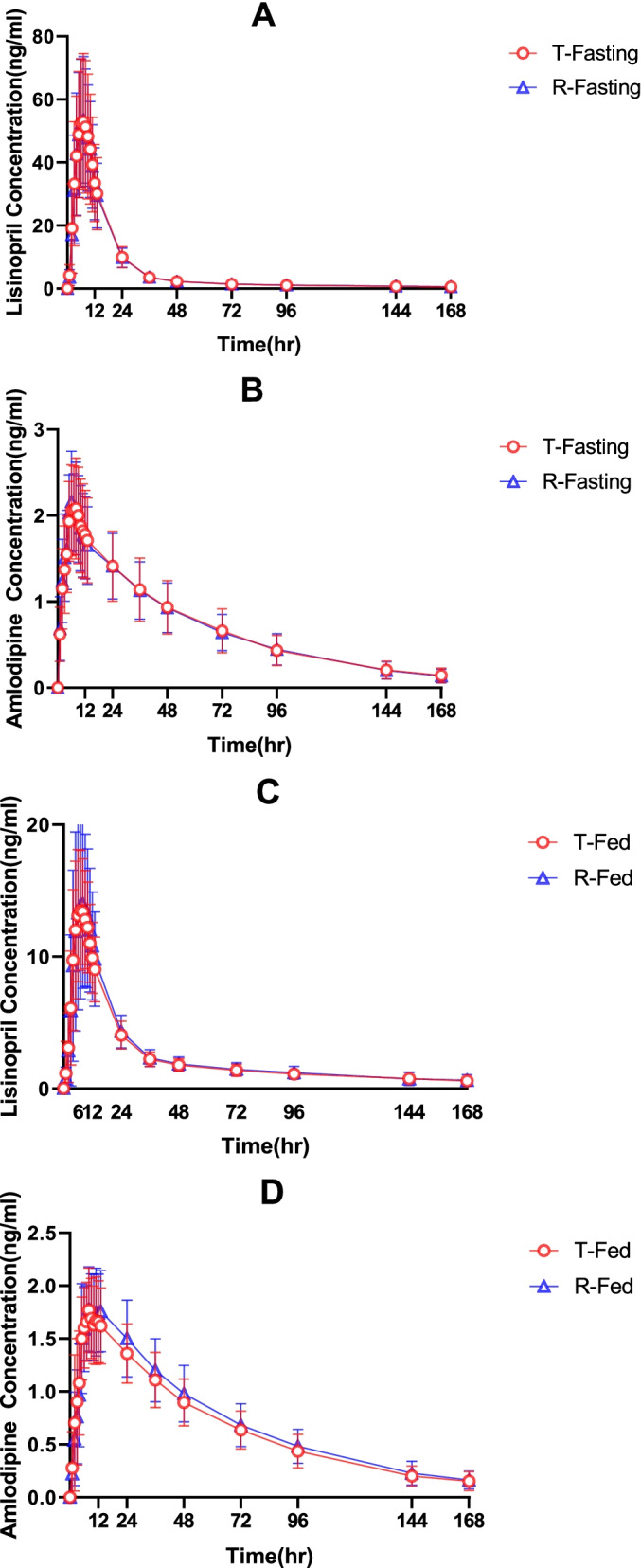
Mean plasma concentration versus time profiles of lisinopril (**A**), amlodipine (**B**) under fasting conditions and lisinopril (**C**), amlodipine (**D**) under fed conditions, following a single dose of the test and reference Lisinopril/amlodipine tablets in Chinese subjects

The mean plasma concentration versus time profiles of lisinopril and amlodipine following a single dose of the test or reference products under fasting and fed conditions are illustrated in Fig. 2, the PK parameters are summarized in Table 2.

**Table 2:** Pharmacokinetic parameters of lisinopril and amlodipine following a single dose of the test and reference formulations under fasting and fed conditions, presented as arithmetic mean ± SD

	Fasting group		Fed group	
	T (*N* = 40)	R (*N* = 39)	T (*N* = 38)	R (*N* = 37)
**Lisinopril**				
C_max_(ng/mL)	55.1 ± 22.7	54.8 ± 20.4	14.1 ± 4.52	14.8 ± 6.80
T_max_ (h)	6.80 ± 1.32	6.54 ± 1.14	7.34 ± 1.48	7.95 ± 1.79
AUC_0-t_ (h × ng/mL)	950 ± 334	940 ± 286	385 ± 92.7	402 ± 120
AUC_0-∞_(h × ng/mL)	1040 ± 353	1030 ± 294	494 ± 140	506 ± 155
T_1/2Z_ (h)	90.6 ± 35.0	92.8 ± 20.7	98.6 ± 29.2	89.9 ± 27.4
AUC_%Extrap	9.28 ± 4.81	9.26 ± 3.19	21.2 ± 6.71	20.2 ± 6.52
**Amlodipine**				
C_max_(ng/mL)	2.30 ± 0.574	2.31 ± 0.586	1.95 ± 0.414	1.98 ± 0.398
T_max_ (h)	6.48 ± 1.88	6.54 ± 1.80	7.97 ± 2.91	8.49 ± 2.70
AUC_0-t_ (h × ng/mL)	118 ± 38.2	117 ± 35.5	111 ± 26.6	120 ± 30.6
AUC_0-∞_(h × ng/mL)	128 ± 44.2	126 ± 41.0	122 ± 34.4	131 ± 37.0
T_1/2Z_ (h)	42.5 ± 6.2	42.1 ± 6.66	44.3 ± 9.71	44.9 ± 8.95

Regarding the C_max_, AUC_0-t_, and AUC_0-∞_ of lisinopril and amlodipine respectively, the 90% CIs for the GMRs fell within the predefined acceptance range of 80–125%, and provided supportive evidence for bioequivalence (Table 3). In the fasting study, although the sample collected at 168 h after administration did not reach 3–5 half-lives, the last detectable concentration of all subjects was lower than 1/20 of the corresponding peak concentration and only 2.5% (2/79) of AUC__%Extrap_ was more than 20%. Therefore, the plasma concentration from 0-168 h can completely describe the pharmacokinetic behavior of lisinopril. Compared with the fasting study, the C_max_ and AUC of lisinopril under fed condition were significantly reduced. Although 54.5% (40/74) of AUC__%Extrap_ was higher than 20%, 89.2% (66/74) of the final concentration at 168 h were lower than 1/10 of the corresponding peak concentration, which could basically describe the pharmacokinetic behavior of lisinopril. After eliminating the data with AUC__%Extrap_ greater than 20% for sensitivity analysis, the 90% CI for the GMRs of AUC_0-∞_ of the test and reference preparation was 96.2% (86.7–106.7%).

**Table 3:** The geometric mean ratios of primary pharmacokinetic parameters for lisinopril and amlodipine and their 90%CIs under fasting and fed conditions

	Geometric mean		T/R (%)	Intra-CV (%)	90%CIs
	T	R			
**Lisinopril, fasting**					
C_max_(ng/mL)	50.71	51.12	99.19	24.3	90.54 ~ 108.67
AUC_0-t_(h × ng/mL)	890.83	898.61	99.05	19.3	92.10 ~ 106.52
AUC_0-∞_(h × ng/mL)	982.55	991.14	99.13	17.0	92.97 ~ 105.71
**Amlodipine, fasting**					
C_max_(ng/mL)	2.23	2.25	99.3	9.7	95.70 ~ 103.04
AUC_0-t_ (h × ng/mL)	112.1	113.27	98.97	9.9	95.30 ~ 102.78
AUC_0-∞_(h × ng/mL)	120.52	121.66	99.07	10.5	95.19 ~ 103.04
**Lisinopril, fed**					
C_max_(ng/mL)	13.49	13.11	102.95	22.4	94.28 ~ 112.41
AUC_0-t_(h × ng/mL)	376.67	383.16	98.31	13.3	93.28 ~ 103.61
AUC_0-∞_(h × ng/mL)	483.99	484.98	99.79	12.9	94.83 ~ 105.02
**Amlodipine, fed**					
C_max_(ng/mL)	1.88	1.9	98.94	11.6	94.51 ~ 103.57
AUC_0-t_ (h × ng/mL)	105.04	112.61	93.28	9.3	89.92 ~ 96.77
AUC_0-∞_(h × ng/mL)	114.06	122.33	93.24	9.8	89.67 ~ 96.65

Abbreviation: *CI* Confidence interval, *Intra-CV* Intra-subject coefficient of variation

A high-fat breakfast appeared to produce an alteration in the C_max_ and AUC of lisinopril after a dose of either reference or test drug in Chinese healthy subjects. Compared with fasting study, after high -fat postprandial administration, lisinopril C_max_, AUC_0-t_, and AUC_0-∞_ under fed condition were greatly decreased by 74%, 59%, 53% for test products (*P* < 0.001), and 73%, 57%, 51% for reference products (*P* < 0.001). In addition, there was a nearly 1.5-h delay in median T_max_ under fed conditions for the test products. However, no changes were observed in T_max_ for reference products and in T_1/2_ for both the test and reference products between the two fasting and fed studies.

### Safety assessment

**Fig. 3:**
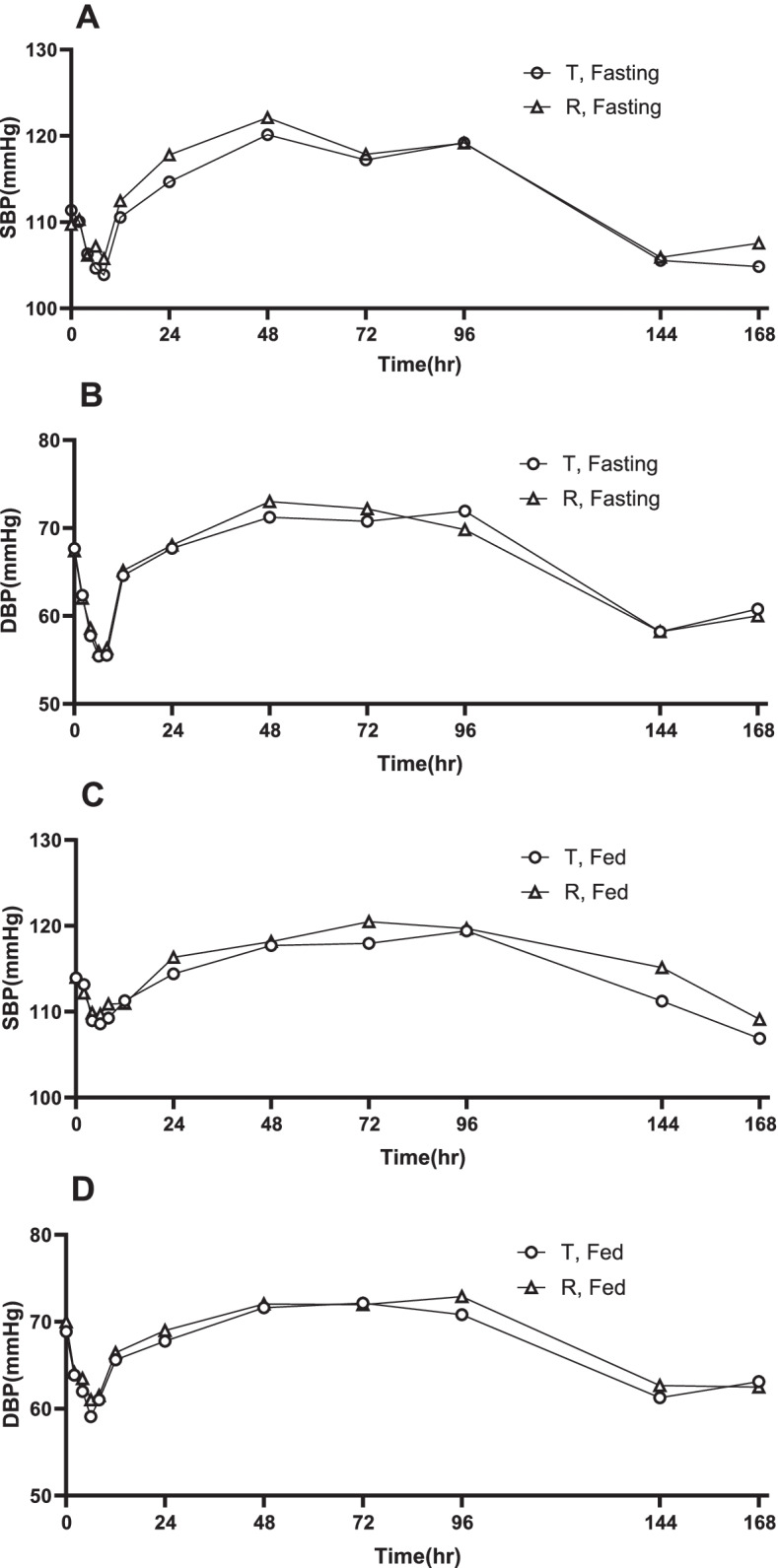
Mean (**A**) systolic blood pressure (SBP), (**B**) diastolic blood pressure (DBP) under fasting condition and mean (**C**) SBP, (**D**) DBP under fed condition following a single administration of test and reference Lisinopril/amlodipine tablets in Chinese subjects

The test and reference Lisinopril/amlodipine tablets showed good tolerance in all subjects. During the study, the vital signs of subjects were stable except that some subjects had signs of blood pressure reduction due to the expected effect of the study drug, and there was no clinically significant change in the follow-up laboratory examination after the administration compared with the baseline value. In the study of fasting condition, a total of 33 treatment emergent adverse events (TEAEs) were recorded in 20 subjects (50% of 40 subjects) after T treatment, and 25 TEAEs were recorded in 16 subjects (40% of 40 subjects) after R treatment. In the fed study, 13 TEAEs were recorded in 10 subjects (22.7% of 44 subjects) after T treatment, and 12 TEAEs were recorded in 9 subjects (20.5% of 44 subjects) after R treatment. All AEs were light and spontaneously recovered without specific intervention except for one instance of tonsillitis, which may be irrelevant to the study drugs, and a case of atopic dermatitis. No subjects withdrew from the study due to AEs except for one case of tonsillitis and no severe adverse events (SAE) occurred. There was no significant difference in the incidence of AEs between the two treatments. All TEAEs were summarized according to system organ classification (SOC) and preferred term (PT), and were presented in Table 4. Hypotension was the most common AE, and Fig. 3 illustrates the changes in mean systolic blood pressure (SBP) and diastolic blood pressure (DBP) from baseline to 168 h. The results showed that the blood pressure decreased maximally from pre-dose values by 6 h after one dose of Lisinopril/amlodipine tablets and the suppression lasted up to 12 h. The changes in blood pressure showed no statistical differences between the test and reference groups under both fasting and fed conditions. Although the difference comparison in mean BP between fasting and fed groups was not the objective of our study, we tried to make an exploratory pharmacodynamic comparison. There was no significant difference in mean SBP between the fasting and fed groups, except at 8 h after administration of test product (*P* = 0.038). While the DBP decreased more at 4 h, 6 h, 8 h following the dosing with both test and reference regimens in the fasting state, compared to fed study (*P* < 0.05).

**Table 4:** Treatment emergent adverse events (TEAEs) after a single dose of the test and reference formulations under fasting and fed conditions

AE	T, fasting		R, fasting		T, fed		R, fed	
	nTEAE	n (%)	nTEAE	n (%)	nTEAE	n (%)	nTEAE	n (%)
Hypotension	26	18 (45)	21	15 (37.5)	9	7 (15.9)	7	5 (11.4)
Headache	0	0	1	1 (2.5)	0	0	0	0
Dizziness	1	1 (2.5)	1	1 (2.5)	0	0	0	0
Sinus tachycardia	1	1 (2.5)	0	0	0	0	0	0
Fever	1	1 (2.5)	1	1 (2.5)	0	0	0	0
Hypertriglyceridenmia	3	2 (5)	0	0	1	1 (2.3)	1	1 (2.3)
Leukocyturia	0	0	1	1 (2.5)	0	0	1	1 (2.3)
Blood bilirubin increased	0	0	0	0	0	0	1	1(2.3)
Creatine kinase increased	0	0	0	0	0	0	1	1 (2.3)
Hypophosphatemia	0	0	0	0	0	0	1	1 (2.3)
Atopic dermatitis	0	0	0	0	1	1 (2.3)	0	0
2nd degree Atrioventricular block	0	0	0	0	1	1 (2.3)	0	0
Anaemia	1	1 (2.5)	0	0	1	1 (2.3)	0	0

## Discussion

The purpose of this study was to compare the PK properties to examine whether the new Lisinopril/amlodipine tablets was equivalent to the reference for a new drug application to the NMPA. In this study, the GMR and its 90% CI for the C_max_, AUC_0-t_, and AUC_0-∞_ of lisinopril and amlodipine respectively, under both fasting and fed conditions, fell within the conventional bioequivalence criteria of 0.80–1.25. In addition, compared with the reference drug, the incidence of AEs of the test drug had no difference, and showed similar safety and tolerance. These results indicated that the two Lisinopril/amlodipine tablets were bioequivalent and exchangeable in clinical practice.

In our study, lisinopril in the fix-combination formulations was absorbed slowly with the C_max_ occurring at 6 h which is similar to literatures [8–11]. The plasma concentration decrease slowly and according to the reviews of lisinopril from FDA, the T_1/2_ was 55 ± 38 h in fasting study and 57 ± 40 h in fed study [17]. The measured T_1/2_ in this study was about 90 h, a bit longer than recorded previously, which may be related to the binding saturation of the drug and ACE. The T_max_ of Amlodipine in the study was about 6 h and the measured T_1/2_ was approximately 40 h, which is consistent with previous report [13, 14].

Although the study was primarily designed to evaluate the bioequivalence of two Lisinopril/amlodipine tablets, the results indicated there may be a food effect, based on the parallel comparison of lisinopril PK parameters between fasting and fed study, which are inconsistent with the instructions of the original lisinopril tablet (Zestril® produced by AstraZeneca UK limited) and the reference Lisonorm (produced by Gedeon Richter Ltd). The instructions say the gastrointestinal absorption of lisinopril is not affected by food. A previous study investing the influence of food consumption on the rate or extent of absorption of orally administered lisinopril in healthy volunteers observed that, a breakfast (524 kcal, consisting of one fried egg, two pieces of toast or bread, 20 g of orange marmalade or jelly, two stripes of bacon, 150 ml of skimmed milk and 100 ml of orange juice) did not affect the bioavailability of Lisinopril [18]. This inconsistency may probably be due to the fact that (i) high-caloric and high-fat foods have a more obvious impact on the physiology of the gastrointestinal tract and lead to more significant changes in the bioavailability of pharmaceuticals [19]; (ii) spinach in breakfast is rich in oxalic acid, which may change gastrointestinal PH and gastrointestinal peristalsis [20]; and (iii) the participants of this study were all young Chinese adults, and there may be ethnic differences in the pharmacokinetics of lisinopril. Among ACE inhibitors, lisinopril has a unique property that does not require hydrolysis to exert ACE inhibition, and only lisinopril and captopril are not ester prodrugs and less lipophilic [21]. Food has been shown to reduce the bioavailability of captopril by 35% to 50% after a single oral administration, but not the bioavailability of inhibitors administered as ester prodrugs [22]. With high solubility, low membrane permeability and poor metabolism, the pharmacokinetic of lisinopril may be dominated by absorptive transporter effects [23]. Only about a quarter of the administered dose is absorbed and the low bioavailability is due to poor gastrointestinal absorption rather than first-pass hepatic metabolism, as demonstrated by the fact that mean fecal recovery of lisinopril was 69% of intact drug [24].

In order to better understand the possible reasons for the decrease of lisinopril absorption in the fed study, we searched for various factors that affect lisinopril bioavailability. Little is known about pharmacokinetic interaction of lisinopril so far. Drugs that often used with lisinopril, such as nifedipine, digoxin, hydrochlorothiazide, have no substantial effect on the pharmacokinetics of Lisinopril [25–27]. No drug-drug interactions (DDIs) were found between the active components amlodipine and lisinopril. One of the factors that has been reported to affect the kinetic properties of lisinopril was age. Drug concentrations of elderly patients (> 65 years) have been reported to be approximately double those of younger patients [28]. And a recent study demonstrated that a concomitant ingestion of epigallocatechin gallate (EGCG)-concentrated green tea extract significantly decreased lisinopril C_max_, AUC_0-24_ and AUC_0-∞_ by 71%, 69% and 67%, without altering renal clearance of Lisinopril [29]. However, in the present study, the enrolled subjects was all between 18 and 50 years old, and those who have drink too much tea were excluded. Moreover, it was forbidden to take tea within 48 h before taking the first administration and during the test.

We acknowledge a number of limitations in our study. First, 16 subjects in the fed study fell off due to various issues and 12 substitute subjects re included in the study, which may have slight influence on study data. Second, the study has not been primarily designed to assess the food effect, and the sample size might not be sufficient to demonstrate the presence of food effect in parallel fashion. In addition, the lisinopril has a large inter-subject variability in bioavailability (6–60%), which may introduced a bias when making comparisons between different groups of subjects. Additional studies using a cross-over design approach may actually be needed to evaluate the effect of food on the bioavailability of Lisinopril in Chinese. In addition, the effect of different meal composition may also be evaluated in future studies.

## Conclusion

In conclusion, the new Lisinopril/amlodipine tablets (specification: lisinopril 10 mg / amlodipine 5 mg) developed by Sichuan Sunrise Biopharm Co. Ltd (Sichuan Province, China) are equivalent to the Lisonorm (specification: lisinopril 10 mg / amlodipine 5 mg) produced by Gedeon Richter Ltd (Hungary). If the test formulation can be approved by NMPA, it can be used in the treatment of hypertension in Chinese adult patients. Since the study suggested that a high-fat meal could reduce the bioavailability of lisinopril in both test and reference formulations, further investigations may be required to determine the clinical impact of this observation and confirm dosing recommendations in Chinese.

## Supplementary Information

**Additional file 1: Table S1.** Plasma concentration of lisinopril (ng/ml) after oral administration of test Lisinopril/amlodipine tablets (lisinopril 10mg / amlodipine 5 mg) to 40 subjects under fasting condition. **Table S2.** Plasma concentration of lisinopril (ng/ml) after oral administration of reference Lisinopril/amlodipine tablets (lisinopril 10mg / amlodipine 5 mg) to 39 subjects under fasting conditions. **Table S3. **Plasma concentration of amlodipine (ng/ml) after oral administration of test Lisinopril/amlodipine tablets (lisinopril10mg / amlodipine 5 mg) to 40 subjects under fasting condition. **Table S4.** Plasma concentration of amlodipine (ng/ml) after oral administration of reference Lisinopril/amlodipine tablets (lisinopril 10mg / amlodipine 5 mg) to 39 subjects under fasting condition. **Table S5.** Plasma concentration of lisinopril (ng/ml) after oral administration of test Lisinopril/amlodipine tablets (lisinopril 10mg / amlodipine 5 mg) to 38 subjects under fed condition. **Table S6.** Plasma concentration of lisinopril (ng/ml) after oral administration of reference Lisinopril/amlodipine tablets (lisinopril 10mg / amlodipine 5 mg) to 37 subjects under fed condition. **Table S7.** Plasma concentration of amlodipine (ng/ml) after oral administration of test Lisinopril/amlodipine tablets (lisinopril 10mg / amlodipine 5 mg) to 38 subjects under fed condition. **Table S8.** Plasma concentration of amlodipine (ng/ml) after oral administration of reference Lisinopril/amlodipine tablets (lisinopril 10mg / amlodipine 5 mg) to 37 subjects under fed condition. **Table S9.** Changes of SBP after drug administration under fasting condition. **Table S10.** Changes of DBP after drug administration under fasting condition. **Table S11.** Changes of SBP after drug administration under fed condition. **Table S12.** Changes of DBP after drug administration under fed condition.

## Data Availability

All data generated or analysed during this study are included in the supplementary file.
