# Supplementary material for: Bioequivalence evaluation and food effect assessment of Lisinopril/amlodipine tablets in healthy Chinese subjects under fasting and fed conditions
Source: BMC Pharmacol Toxicol. 2022 Jul 7;23:45. doi: 10.1186/s40360-022-00590-6 (PMC9260974; doi:10.1186/s40360-022-00590-6)
Supplement: Supplementary file 1 — Additional file 1: Table S1. Plasma concentration of lisinopril (ng/ml) after oral administration of test Lisinopril/amlodipine tablets (lisinopril 10mg / amlodipine 5 mg) to 40 subjects under fasting condition. Table S2. Plasma concentration of lisinopril (ng/ml) after oral administration of reference Lisinopril/amlodipine tablets (lisinopril 10mg / amlodipine 5 mg) to 39 subjects under fasting conditions. Table S3. Plasma concentration of amlodipine (ng/ml) after oral administration of test Lisinopril/amlodipine tablets (lisinopril10mg / amlodipine 5 mg) to 40 subjects under fasting condition. Table S4. Plasma concentration of amlodipine (ng/ml) after oral administration of reference Lisinopril/amlodipine tablets (lisinopril 10mg / amlodipine 5 mg) to 39 subjects under fasting condition. Table S5. Plasma concentration of lisinopril (ng/ml) after oral administration of test Lisinopril/amlodipine tablets (lisinopril 10mg / amlodipine 5 mg) to 38 subjects under fed condition. Table S6. Plasma concentration of lisinopril (ng/ml) after oral administration of reference Lisinopril/amlodipine tablets (lisinopril 10mg / amlodipine 5 mg) to 37 subjects under fed condition. Table S7. Plasma concentration of amlodipine (ng/ml) after oral administration of test Lisinopril/amlodipine tablets (lisinopril 10mg / amlodipine 5 mg) to 38 subjects under fed condition. Table S8. Plasma concentration of amlodipine (ng/ml) after oral administration of reference Lisinopril/amlodipine tablets (lisinopril 10mg / amlodipine 5 mg) to 37 subjects under fed condition. Table S9. Changes of SBP after drug administration under fasting condition. Table S10. Changes of DBP after drug administration under fasting condition. Table S11. Changes of SBP after drug administration under fed condition. Table S12. Changes of DBP after drug administration under fed condition. [file 40360_2022_590_MOESM1_ESM.docx]

Table S1.Plasma concentration of lisinopril (ng/ml) after oral administration of test Lisinopril/amlodipine tablets (lisinopril 10mg / amlodipine 5 mg) to 40 subjects under fasting condition

|  | | | | | | Nominal_Time | | | | | | | | | | | | | | | | | | | | |
| --- | --- | --- | --- | --- | --- | --- | --- | --- | --- | --- | --- | --- | --- | --- | --- | --- | --- | --- | --- | --- | --- | --- | --- | --- | --- | --- |
|  |  |  |  |  |  | (h) | | | | | | | | | | | | | | | | | | | | |
|  |  |  |  |  |  | 0 | 1 | 2 | 3 | 4 | 5 | 6 | 7 | 8 | 9 | 10 | 11 | 12 | 13 | 24 | 36 | 48 | 72 | 96 | 144 | 168 |
| Group | Analyte | Formulation | Subject | Period | Sequence | Concentration | | | | | | | | | | | | | | | | | | | | |
|  |  |  |  |  |  | (ng/ml) | | | | | | | | | | | | | | | | | | | | |
| FAS | LSN | T | FAS001 | 2 | RT | BQL | 1.62 | 8.80 | 18.0 | 29.7 | 35.3 | 38.0 | 38.9 | 36.4 | 35.0 | 35.7 | 31.5 | 31.4 | 26.6 | 8.68 | 3.76 | 2.59 | 1.78 | 1.42 | 0.916 | 0.888 |
|  | | | FAS002 | 2 | RT | BQL | 10.2 | 18.7 | 27.0 | 33.4 | 38.5 | 42.4 | 44.7 | 45.2 | 41.4 | 40.2 | 33.0 | 29.2 | 27.2 | 8.93 | 3.11 | 2.00 | 1.27 | 1.01 | 0.637 | 0.551 |
|  | | | FAS003 | 1 | TR | BQL | 1.53 | 10.4 | 24.8 | 35.9 | 40.2 | 43.0 | 45.3 | 46.3 | 43.2 | 40.4 | 35.2 | 29.5 | 28.1 | 8.30 | 2.86 | 1.73 | 1.10 | 0.723 | 0.538 | BQL |
|  | | | FAS004 | 1 | TR | BQL | 5.56 | 24.1 | 37.3 | 45.2 | 47.8 | 46.9 | 44.1 | 38.9 | 35.8 | 31.7 | 27.4 | 22.2 | 18.9 | 5.92 | 2.42 | 1.78 | 1.17 | 0.910 | 0.653 | 0.503 |
|  | | | FAS005 | 2 | RT | BQL | 3.03 | 11.0 | 25.9 | 35.9 | 39.0 | 38.3 | 40.4 | 39.1 | 37.3 | 33.0 | 30.2 | 25.2 | 23.6 | 9.30 | 3.73 | 2.50 | 1.58 | 1.19 | 0.952 | 0.862 |
|  | | | FAS006 | 1 | TR | BQL | 5.73 | 36.1 | 72.1 | 90.2 | 97.2 | 105 | 122 | 123 | 114 | 92.0 | 81.3 | 73.0 | 66.0 | 17.6 | 5.04 | 2.63 | 1.34 | 1.00 | 0.731 | 0.628 |
|  | | | FAS007 | 2 | RT | BQL | 2.10 | 11.5 | 21.5 | 28.1 | 32.7 | 31.6 | 30.4 | 28.4 | 27.2 | 25.2 | 22.6 | 18.9 | 18.5 | 6.28 | 2.94 | 2.04 | 1.49 | 1.24 | 0.956 | 0.953 |
|  | | | FAS008 | 1 | TR | BQL | 2.68 | 7.87 | 15.1 | 21.9 | 25.4 | 30.2 | 31.5 | 33.4 | 32.6 | 30.8 | 28.4 | 24.9 | 22.6 | 8.11 | 3.42 | 2.29 | 1.48 | 1.21 | 0.771 | 0.667 |
|  | | | FAS009 | 2 | RT | BQL | 5.33 | 39.2 | 74.1 | 74.4 | 77.3 | 82.6 | 77.8 | 73.8 | 66.2 | 62.5 | 55.5 | 47.9 | 40.5 | 15.0 | 4.97 | 2.84 | 1.57 | 1.06 | 0.801 | 0.715 |
|  | | | FAS010 | 2 | RT | 0.747 | 10.8 | 62.7 | 86.9 | 113* | 99.9 | 96.0 | 90.2 | 85.2 | 88.3 | 80.4 | 72.7 | 57.8 | 53.0 | 15.7 | 4.80 | 2.94 | 1.89 | 1.54 | 1.35 | 1.04 |
|  | | | FAS011 | 1 | TR | BQL | 1.57 | 9.06 | 22.1 | 28.9 | 38.2 | 40.9 | 40.0 | 34.5 | 35.4 | 32.1 | 28.5 | 23.8 | 21.0 | 5.72 | 2.04 | 1.27 | 0.869 | 0.718 | BQL | BQL |
|  | | | FAS012 | 1 | TR | BQL | 2.65 | 8.79 | 19.4 | 30.0 | 39.6 | 45.8 | 46.4 | 45.1 | 41.9 | 37.7 | 32.1 | 28.4 | 26.1 | 9.92 | 3.45 | 2.13 | 1.08 | 0.852 | 0.584 | BQL |
|  | | | FAS013 | 1 | TR | BQL | 3.21 | 18.8 | 38.3 | 45.5 | 49.6 | 47.1 | 45.1 | 43.0 | 39.6 | 37.3 | 34.1 | 28.5 | 24.9 | 8.90 | 3.48 | 2.40 | 1.34 | 1.07 | 0.693 | 0.592 |
|  | | | FAS014 | 2 | RT | BQL | 4.59 | 13.5 | 29.5 | 43.5 | 55.9 | 65.6 | 70.5 | 68.0 | 63.2 | 58.9 | 54.6 | 49.3 | 42.8 | 14.5 | 4.98 | 3.12 | 1.97 | 1.69 | 1.18 | 0.905 |
|  | | | FAS015 | 1 | TR | BQL | 4.30 | 7.80 | 18.7 | 32.7 | 38.3 | 44.5 | 46.4 | 46.7 | 44.9 | 42.6 | 38.7 | 33.6 | 31.5 | 9.72 | 3.40 | 2.51 | 1.96 | 1.55 | 1.13 | 0.947 |
|  | | | FAS016 | 2 | RT | BQL | 8.82 | 34.7 | 58.2 | 69.7 | 70.1 | 73.3 | 76.0 | 74.4 | 76.8 | 71.5 | 60.4 | 49.8 | 46.8 | 17.1 | 5.71 | 3.37 | 2.11 | 1.62 | 1.23 | 0.983 |
|  | | | FAS017 | 1 | TR | BQL | 2.90 | 9.65 | 22.9 | 29.7 | 33.2 | 35.4 | 35.2 | 35.5 | 31.4 | 28.6 | 25.5 | 22.4 | 19.3 | 7.78 | 2.56 | 1.68 | 1.15 | 0.838 | 0.654 | 0.574 |
|  | | | FAS018 | 1 | TR | BQL | 7.12 | 26.5 | 42.6 | 54.9 | 60.4 | 61.7 | 64.6 | 59.1 | 53.0 | 38.2 | 44.4 | 37.1 | 33.1 | 10.1 | 3.19 | 1.99 | 1.23 | 1.09 | 0.861 | 0.632 |
|  | | | FAS019 | 2 | RT | BQL | 3.64 | 32.1 | 49.9 | 64.2 | 73.0 | 76.8 | 79.3 | 72.2 | 62.6 | 61.2 | 51.1 | 43.1 | 38.0 | 10.9 | 3.51 | 1.94 | 1.15 | 0.768 | 0.539 | 0.579 |
|  | | | FAS020 | 2 | RT | BQL | 5.22 | 31.9 | 42.9 | 59.5 | 61.6 | 60.2 | 61.3 | 59.5 | 51.6 | 50.7 | 45.1 | 37.1 | 31.9 | 12.1 | 4.53 | 2.41 | 1.24 | 0.902 | 0.630 | 0.601 |
|  | | | FAS021 | 1 | TR | BQL | 3.62 | 19.1 | 35.0 | 46.4 | 63.4 | 66.5 | 67.3 | 61.7 | 57.4 | 50.5 | 43.8 | 38.5 | 32.0 | 9.01 | 3.09 | 1.90 | 1.15 | 0.965 | 0.643 | BQL |
|  | | | FAS022 | 1 | TR | BQL | 1.08 | 3.58 | 14.7 | 26.8 | 45.5 | 59.6 | 63.9 | 55.6 | 51.7 | 50.8 | 43.3 | 38.2 | 30.8 | 11.4 | 4.03 | 2.47 | 1.20 | 0.871 | 0.601 | BQL |
|  | | | FAS023 | 2 | RT | BQL | 8.89 | 33.6 | 50.4 | 55.1 | 61.1 | 61.5 | 55.6 | 54.5 | 48.5 | 41.5 | 37.0 | 29.6 | 25.3 | 7.60 | 3.04 | 1.76 | 1.00 | 0.691* | 0.533 | BQL |
|  | | | FAS024 | 2 | RT | BQL | 1.39 | 3.94 | 11.1 | 25.6 | 31.6 | 34.9 | 35.9 | 34.9 | 35.7 | 31.2 | 31.6 | 28.2 | 25.0 | 9.20 | 3.56 | 2.00 | 1.27 | 0.958 | 0.586 | 0.526 |
|  | | | FAS025 | 2 | RT | BQL | 2.82 | 26.9 | 51.8 | 67.4 | 70.5 | 84.3 | 82.1 | 81.5 | 81.3 | 74.6 | 68.0 | 41.1 | 50.3 | 15.0 | 4.78 | 2.83 | 1.64 | 1.38 | 0.924 | 0.830 |
|  | | | FAS026 | 2 | RT | 0.609 | 10.5 | 29.3 | 39.1 | 47.7 | 54.2 | 58.1 | 62.5 | 59.5 | 55.0 | 48.9 | 43.4 | 36.0 | 33.1 | 11.7 | 5.16 | 3.68 | 2.56 | 2.17 | 1.26 | 1.20 |
|  | | | FAS027 | 1 | TR | BQL | 4.90 | 13.3 | 27.3 | 37.7 | 43.0 | 50.2 | 54.6 | 52.1 | 50.8 | 48.4 | 41.2 | 38.4 | 34.2 | 10.6 | 3.16 | 2.34 | 1.37 | 0.942 | 0.739 | 0.641 |
|  | | | FAS028 | 1 | TR | BQL | 1.25 | 10.6 | 25.5 | 35.4 | 39.2 | 42.4 | 40.7 | 39.2 | 35.5 | 32.3 | 27.5 | 24.5 | 21.3 | 6.63 | 2.27 | 1.60 | 0.851 | 0.702 | BQL | BQL |
|  | | | FAS029 | 2 | RT | BQL | 6.38 | 32.5 | 48.7 | 59.1 | 62.2 | 65.4 | 62.0 | 58.1 | 53.3 | 50.2 | 43.6 | 36.4 | 34.7 | 12.1 | 4.19 | 2.71 | 1.51 | 1.14 | 0.893 | 0.782 |
|  | | | FAS030 | 1 | TR | BQL | 0.692 | 1.57 | 2.79 | 7.88 | 13.4 | 18.5 | 17.6 | 18.3 | 18.6 | 16.8 | 15.4 | 13.8 | 12.2 | 4.91 | 1.93 | 1.35 | 0.875 | 0.619 | 0.671 | BQL |
|  | | | FAS031 | 1 | TR | BQL | BQL | 1.11 | 1.27 | 3.65 | 8.83 | 12.4 | 14.1 | 14.8 | 14.1 | 12.8 | 11.7 | 10.8 | 9.15 | 3.06 | 1.33 | 0.981 | 0.687 | BQL | BQL | BQL |
|  | | | FAS032 | 2 | RT | BQL | 14.8 | 54.2 | 70.4 | 84.0 | 82.2 | 83.1 | 86.7 | 87.7 | 78.4 | 73.0 | 58.1 | 47.5 | 41.5 | 10.6 | 2.72 | 1.81 | 1.14 | 1.07 | 0.673 | 0.563 |
|  | | | FAS033 | 2 | RT | BQL | 2.72 | 7.56 | 21.4 | 34.2 | 39.9 | 45.2 | 44.5 | 45.3 | 43.0 | 40.7 | 37.9 | 33.7 | 31.7 | 11.3 | 3.93 | 2.50 | 1.28 | 0.884 | 0.541 | 0.546 |
|  | | | FAS034 | 1 | TR | BQL | 2.51 | 9.14 | 19.8 | 31.8 | 37.2 | 41.8 | 39.1 | 39.2 | 37.0 | 33.6 | 31.1 | 26.7 | 23.7 | 8.37 | 2.63 | 1.73 | 1.03 | 0.721 | 0.516 | BQL |
|  | | | FAS035 | 2 | RT | BQL | 2.95 | 17.0 | 25.8 | 34.7 | 32.8 | 32.6 | 33.3 | 28.9 | 26.8 | 26.2 | 22.9 | 19.3 | 16.7 | 6.36 | 2.80 | 1.90 | 1.37 | 1.04 | 0.900 | 0.823 |
|  | | | FAS036 | 1 | TR | BQL | 2.78 | 14.1 | 27.1 | 37.9 | 41.5 | 49.5 | 54.7 | 57.1 | 56.6 | 57.6 | 49.2 | 44.6 | 40.7 | 14.4 | 5.15 | 2.83 | 1.66 | 1.27 | 0.863 | 0.759 |
|  | | | FAS037 | 1 | TR | BQL | 0.813 | 2.05 | 5.54 | 15.7 | 24.2 | 28.7 | 30.5 | 29.5 | 29.2 | 27.1 | 24.4 | 22.3 | 19.4 | 7.25 | 3.01 | 1.90 | 1.35 | 0.755 | 0.583 | 0.575 |
|  | | | FAS038 | 1 | TR | BQL | 3.90 | 27.9 | 43.3 | 51.2 | 56.9 | 68.0 | 67.4* | 64.1 | 58.6 | 51.5 | 45.9 | 41.8 | 36.2 | 10.5 | 3.33 | 2.03 | 1.34 | 1.26 | 0.890 | 0.726 |
|  | | | FAS039 | 2 | RT | BQL | 2.65 | 20.9 | 33.9 | 45.5 | 47.0 | 49.5 | 47.9 | 41.6 | 37.9 | 34.7 | 30.8 | 27.2 | 22.9 | 7.98 | 2.87 | 2.07 | 1.48 | 1.07 | 0.652 | 0.648 |
|  | | | FAS040 | 2 | RT | BQL | 2.12 | 12.2 | 26.7 | 37.2 | 42.6 | 42.8 | 41.6 | 39.0 | 36.3 | 34.6 | 31.8 | 28.3 | 24.4 | 9.68 | 3.98 | 2.65 | 1.41 | 1.00 | 0.699 | 0.593 |
|  | | |  | | N | 40 | 40 | 40 | 40 | 39 | 40 | 40 | 39 | 40 | 40 | 40 | 40 | 40 | 40 | 40 | 40 | 40 | 40 | 39 | 40 | 40 |
|  | | |  | | Mean | 0.0339 | 4.23 | 19.1 | 33.2 | 42.0 | 48.8 | 52.5 | 52.9 | 51.3 | 48.2 | 44.2 | 39.3 | 33.5 | 30.1 | 9.96 | 3.52 | 2.23 | 1.37 | 1.06 | 0.724 | 0.546 |
|  | | |  | | SD | 0.150 | 3.26 | 14.2 | 19.7 | 19.0 | 20.0 | 20.4 | 21.6 | 21.0 | 19.8 | 17.4 | 15.0 | 12.2 | 11.4 | 3.33 | 1.01 | 0.568 | 0.372 | 0.364 | 0.299 | 0.354 |
|  |  |  |  |  | CV% | 443.8 | 77.0 | 74.1 | 59.4 | 45.3 | 41.1 | 38.8 | 40.7 | 41.0 | 41.1 | 39.3 | 38.2 | 36.3 | 38.0 | 33.5 | 28.6 | 25.5 | 27.1 | 34.4 | 41.3 | 64.9 |
|  | | |  | | Min | 0.00 | 0.00 | 1.11 | 1.27 | 3.65 | 8.83 | 12.4 | 14.1 | 14.8 | 14.1 | 12.8 | 11.7 | 10.8 | 9.15 | 3.06 | 1.33 | 0.981 | 0.687 | 0.00 | 0.00 | 0.00 |
|  | | |  | | Max | 0.747 | 14.8 | 62.7 | 86.9 | 90.2 | 99.9 | 105 | 122 | 123 | 114 | 92.0 | 81.3 | 73.0 | 66.0 | 17.6 | 5.71 | 3.68 | 2.56 | 2.17 | 1.35 | 1.20 |

BQL: lower than the lower limit of quantification; *: If the sampling time exceeds the time window, it will not be included in the descriptive statistical analysis

Table S2.Plasma concentration of lisinopril (ng/ml) after oral administration of reference Lisinopril/amlodipine tablets (lisinopril 10mg / amlodipine 5 mg) to 39 subjects under fasting conditions

|  | | | | | | Nominal_Time | | | | | | | | | | | | | | | | | | | | |
| --- | --- | --- | --- | --- | --- | --- | --- | --- | --- | --- | --- | --- | --- | --- | --- | --- | --- | --- | --- | --- | --- | --- | --- | --- | --- | --- |
|  |  |  |  |  |  | (h) | | | | | | | | | | | | | | | | | | | | |
|  |  |  |  |  |  | 0 | 1 | 2 | 3 | 4 | 5 | 6 | 7 | 8 | 9 | 10 | 11 | 12 | 13 | 24 | 36 | 48 | 72 | 96 | 144 | 168 |
| Group | Analyte | Formulation | Subject | Period | Sequence | Concentration | | | | | | | | | | | | | | | | | | | | |
|  |  |  |  |  |  | (ng/ml) | | | | | | | | | | | | | | | | | | | | |
| FAS | LSN | R | FAS001 | 1 | RT | BQL | 4.01 | 36.7 | 60.9 | 72.6 | 66.3 | 69.9 | 66.8 | 64.1 | 63.5 | 58.8 | 52.2 | 42.7 | 39.5 | 10.6 | 3.32 | 1.94 | 1.35 | 1.07 | 0.727 | 0.661 |
|  | | | FAS002 | 1 | RT | BQL | 1.20 | 3.30 | 7.20 | 14.2 | 18.3 | 22.0 | 23.9 | 23.1 | 22.8 | 20.9 | 18.8 | 16.4 | 15.2 | 5.68 | 2.59 | 1.52 | 0.965 | 0.853 | 0.603 | 0.536 |
|  | | | FAS003 | 2 | TR | BQL | 1.77 | 15.3 | 35.9 | 49.6 | 51.5 | 55.6 | 61.8 | 59.5 | 59.0 | 50.7 | 44.4 | 37.5 | 33.9 | 10.1 | 3.05 | 1.96 | 1.40 | 1.01 | 0.721 | 0.617 |
|  | | | FAS004 | 2 | TR | BQL | 4.66 | 23.0 | 46.0 | 57.4 | 60.9 | 57.4 | 57.9 | 51.1 | 42.8 | 38.9 | 32.0 | 26.4 | 23.1 | 7.24 | 2.60 | 1.88 | 1.44 | 1.07 | 0.772 | 0.660 |
|  | | | FAS005 | 1 | RT | BQL | 1.74 | 10.6 | 25.7 | 38.6 | 44.3 | 47.3 | 49.0 | 46.0 | 44.4 | 41.6 | 39.2 | 33.6 | 30.2 | 10.0 | 3.92 | 2.38 | 1.48 | 1.11 | 0.808 | 0.716 |
|  | | | FAS006 | 2 | TR | BQL | 2.86 | 35.5 | 56.8 | 76.0 | 89.9 | 97.1 | 93.4 | 91.1 | 84.9 | 82.1 | 75.5 | 65.3 | 59.0 | 17.6 | 5.25 | 2.90 | 1.71 | 1.18 | 0.808 | 0.711 |
|  | | | FAS007 | 1 | RT | BQL | 2.30 | 10.0 | 25.8 | 34.5 | 42.6 | 46.6 | 47.3 | 45.2 | 41.6 | 38.2 | 36.3 | 31.4 | 28.0 | 10.2 | 3.47 | 2.44 | 1.44 | 1.08 | 0.775 | 0.713 |
|  | | | FAS008 | 2 | TR | BQL | 5.57 | 16.2 | 25.2 | 32.5 | 36.6 | 40.5 | 43.5 | 41.0 | 39.5 | 36.3 | 33.2 | 28.6 | 27.5 | 9.17 | 3.61 | 2.40 | 1.73 | 1.35 | 0.872 | 0.821 |
|  | | | FAS009 | 1 | RT | BQL | 1.54 | 9.23 | 34.0 | 48.1 | 54.7 | 61.2 | 64.5 | 61.0 | 60.5 | 54.1 | 46.5 | 38.4 | 34.4 | 11.3 | 4.28 | 2.45 | 1.33 | 1.15 | 0.793 | 0.635 |
|  | | | FAS010 | 1 | RT | BQL | 1.55 | 11.1 | 22.2 | 35.0 | 36.6 | 42.5 | 46.4* | 43.8 | 40.5 | 36.8 | 32.3 | 29.4 | 24.7 | 7.67 | 3.31 | 2.25 | 1.63 | 1.73 | 1.30 | 1.14 |
|  | | | FAS011 | 2 | TR | BQL | 1.50 | 4.97 | 11.4 | 18.8 | 23.1 | 25.3 | 25.4 | 25.4 | 23.8 | 23.1 | 19.9 | 17.1 | 14.3 | 5.69 | 2.29 | 1.50 | 1.04 | 0.761 | 0.546 | BQL |
|  | | | FAS012 | 2 | TR | BQL | 4.17 | 19.6 | 34.8 | 44.4 | 51.7 | 57.4 | 53.9 | 55.8 | 47.6 | 45.5 | 41.4 | 34.6 | 28.1 | 12.1 | 3.86 | 2.48 | 1.27 | 1.01 | 0.680 | 0.560* |
|  | | | FAS013 | 2 | TR | BQL | 3.97 | 18.8 | 39.5 | 46.4 | 47.2 | 47.5 | 45.7 | 42.8 | 39.5 | 36.9 | 32.6 | 27.4 | 25.0 | 7.93 | 3.54 | 2.48 | 1.47 | 0.982 | 0.804 | 0.653 |
|  | | | FAS014 | 1 | RT | BQL | 4.52 | 25.1 | 42.4 | 57.1 | 70.3 | 78.0 | 80.5 | 75.5 | 69.8 | 62.3 | 54.6 | 47.5 | 43.2 | 13.8 | 5.30 | 3.18 | 1.92 | 1.65 | 1.18 | 0.950 |
|  | | | FAS015 | 2 | TR | BQL | 2.79 | 11.2 | 26.6 | 40.2 | 44.5 | 50.1 | 55.6 | 55.8 | 54.0 | 51.2 | 46.1 | 39.6 | 38.4 | 11.2 | 4.41 | 2.79 | 2.06 | 1.64 | 1.22 | 1.01 |
|  | | | FAS016 | 1 | RT | BQL | 3.09 | 7.18 | 18.7 | 24.6 | 32.9 | 38.0 | 41.9 | 45.4 | 45.6 | 46.4 | 40.6 | 35.8 | 30.3 | 10.9 | 4.18 | 3.05 | 1.60 | 1.22 | 0.920 | 0.791 |
|  | | | FAS017 | 2 | TR | BQL | 2.09 | 5.05 | 11.1 | 20.0 | 21.3 | 25.7 | 26.6 | 25.6 | 24.7 | 23.9 | 20.9 | 17.7 | 16.3 | 5.98 | 2.60 | 1.82 | 1.13 | 0.879 | 0.662 | 0.595 |
|  | | | FAS018 | 2 | TR | BQL | 3.46 | 19.3 | 32.7 | 43.0 | 48.0 | 51.9 | 43.5 | 42.6 | 39.8 | 37.6 | 35.1 | 29.5 | 26.0 | 7.05 | 2.99 | 1.94 | 1.51 | 1.35 | 0.776 | 0.733 |
|  | | | FAS019 | 1 | RT | BQL | 4.50 | 22.5 | 34.7 | 49.6 | 57.2 | 62.5 | 71.0 | 65.2 | 58.5 | 50.0 | 44.5 | 36.3 | 30.7 | 9.23 | 2.93 | 1.67 | 0.990 | 0.728 | 0.578 | BQL |
|  | | | FAS020 | 1 | RT | BQL | 3.29 | 33.8 | 65.8 | 86.0 | 94.2 | 97.3 | 99.9 | 95.2 | 90.4 | 81.0 | 69.8 | 59.3 | 53.2 | 18.7 | 5.62 | 2.85 | 1.35 | 0.947 | 0.753 | 0.533 |
|  | | | FAS021 | 2 | TR | BQL | 4.04 | 16.5 | 36.3 | 60.8 | 77.8 | 76.2 | 75.0 | 67.9 | 60.7 | 56.0 | 46.9 | 40.4 | 35.0 | 10.3 | 3.56 | 2.22 | 1.35 | 0.863 | 0.681 | 0.536 |
|  | | | FAS022 | 2 | TR | BQL | 1.35 | 7.25 | 18.7 | 33.3 | 56.0 | 74.2 | 76.1 | 73.6 | 65.0 | 59.0 | 52.0 | 45.6 | 37.4 | 14.4 | 4.41 | 2.57 | 1.42 | 1.01 | 0.679 | 0.506 |
|  | | | FAS023 | 1 | RT | BQL | 13.4 | 50.5 | 69.3 | 83.8 | 92.2 | 90.8 | 83.6 | 83.3 | 67.3 | 62.5 | 55.0 | 44.5 | 39.6 | 10.7 | 2.96 | 1.67 | 0.968 | 0.774 | 0.605 | BQL |
|  | | | FAS024 | 1 | RT | BQL | 1.23 | 3.67 | 8.54 | 17.9 | 26.7 | 31.3 | 32.9 | 34.4 | 32.8 | 32.6 | 29.2 | 25.9 | 23.1 | 8.20 | 2.94 | 1.81 | 1.22 | 0.814 | 0.620 | BQL |
|  | | | FAS025 | 1 | RT | BQL | 2.09 | 11.1 | 25.0 | 40.5 | 47.6 | 58.7 | 58.1 | 58.7 | 54.9 | 52.3 | 45.5 | 44.2 | 37.7 | 12.2 | 3.66 | 2.08 | 1.33 | 1.16 | 0.830 | 0.689 |
|  | | | FAS026 | 1 | RT | BQL | 6.85 | 15.9 | 28.2 | 33.8 | 41.0 | 43.1 | 46.5 | 41.9 | 39.1 | 36.2 | 31.1 | 27.3 | 23.8 | 10.5 | 4.92 | 3.60 | 2.57 | 1.99 | 1.28 | 1.08 |
|  | | | FAS027 | 2 | TR | BQL | 3.34 | 16.0 | 25.1 | 32.3 | 41.2 | 43.8 | 44.7 | 41.7 | 40.3 | 38.6 | 33.3 | 28.6 | 25.2 | 8.27 | 2.89 | 2.26 | 1.33 | 1.01 | 0.725 | 0.693 |
|  | | | FAS028 | 2 | TR | BQL | 1.89 | 4.56 | 14.4 | 26.1 | 35.1 | 39.6 | 38.7 | 37.7 | 36.1 | 34.0 | 30.9 | 26.2 | 23.9 | 10.4 | 3.52 | 2.20 | 1.18 | 0.938 | 0.628 | BQL |
|  | | | FAS029 | 1 | RT | BQL | 4.55 | 13.7 | 28.7 | 35.5 | 39.4 | 40.7 | 43.9 | 41.7 | 40.1 | 35.9 | 30.6 | 26.0 | 23.8 | 7.18 | 3.46 | 2.27 | 1.35 | 1.08 | 0.731 | 0.657 |
|  | | | FAS030 | 2 | TR | BQL | 5.16 | 12.0 | 16.2 | 18.9 | 22.3 | 23.2 | 21.5 | 20.4 | 19.3 | 17.1 | 15.8 | 14.2 | 12.8 | 4.78 | 2.38 | 1.57 | 1.11 | 0.725 | 0.551 | BQL |
|  | | | FAS031 | 2 | TR | BQL | 0.814 | 2.79 | 5.60 | 13.1 | 14.9 | 18.3 | 18.8 | 19.7 | 17.7 | 16.2 | 14.4 | 12.6 | 11.1 | 4.10 | 1.98 | 1.51 | 0.968 | 0.730 | BQL | BQL |
|  | | | FAS032 | 1 | RT | BQL | 10.9 | 53.8 | 74.4 | 87.8 | 80.8 | 86.9 | 86.2 | 76.5 | 72.0 | 63.3 | 50.7 | 43.5 | 38.7 | 9.97 | 2.44 | 1.49 | 0.948 | 0.769 | 0.558 | 0.550 |
|  | | | FAS033 | 1 | RT | BQL | 2.44 | 21.9 | 39.0 | 48.8 | 55.8 | 62.3 | 59.8 | 58.9 | 54.8 | 50.6 | 45.9 | 39.6 | 35.0 | 11.2 | 3.83 | 2.09 | 1.06 | 0.798 | 0.547 | BQL |
|  | | | FAS034 | 2 | TR | BQL | 3.34 | 17.0 | 31.1 | 37.8 | 44.7 | 38.8 | 43.6 | 40.5 | 38.2 | 36.8 | 30.7 | 24.1 | 24.1 | 8.13 | 2.98 | 1.89 | 1.12 | 0.862 | 0.585 | 0.535 |
|  | | | FAS035 | 1 | RT | BQL | 5.77 | 31.0 | 46.9 | 55.3 | 57.4 | 57.9 | 59.8 | 53.5 | 50.9 | 46.8 | 37.5 | 32.9 | 28.9 | 9.15 | 3.92 | 2.57 | 1.71 | 1.14 | 0.842 | 0.789 |
|  | | | FAS036 | 2 | TR | BQL | 2.95 | 6.72 | 15.7 | 33.8 | 47.0 | 57.3 | 63.6 | 59.6 | 58.1 | 56.0 | 48.8 | 40.4 | 35.9 | 13.6 | 5.48 | 3.40 | 2.20 | 1.56 | 0.949 | 0.949 |
|  | | | FAS037 | 2 | TR | BQL | 1.06 | 2.89 | 14.8 | 24.2 | 27.2 | 29.2 | 31.1 | 29.5 | 30.2 | 28.3 | 24.4 | 22.2 | 19.6 | 7.25 | 2.98 | 2.05 | 1.65 | 1.07 | 0.594 | 0.572 |
|  | | | FAS039 | 1 | RT | BQL | 1.24 | 14.2 | 33.3 | 44.4 | 49.4 | 48.9 | 46.8 | 42.8 | 40.6 | 39.3 | 34.9 | 29.0 | 25.5 | 8.75 | 2.70 | 1.74 | 1.09 | 0.845 | 0.627 | 0.504 |
|  | | | FAS040 | 1 | RT | BQL | 3.24 | 22.3 | 39.2 | 45.4 | 51.4 | 49.8 | 51.9 | 51.5 | 48.4 | 42.5 | 37.4 | 32.3 | 29.3* | 10.4 | 4.07 | 2.38 | 1.29 | 0.942 | 0.743 | 0.538 |
|  | | |  | | N | 39 | 39 | 39 | 39 | 39 | 39 | 39 | 38 | 39 | 39 | 39 | 39 | 39 | 38 | 39 | 39 | 39 | 39 | 39 | 39 | 38 |
|  | | |  | | Mean | 0.00 | 3.49 | 17.0 | 31.5 | 42.6 | 48.7 | 52.4 | 53.5 | 51.0 | 47.7 | 44.1 | 38.7 | 33.2 | 29.5 | 9.79 | 3.54 | 2.24 | 1.40 | 1.07 | 0.745 | 0.555 |
|  | | |  | | SD | 0.00 | 2.53 | 12.2 | 17.1 | 19.4 | 19.8 | 20.3 | 20.1 | 18.6 | 16.9 | 15.2 | 13.3 | 11.4 | 10.3 | 3.10 | 0.917 | 0.533 | 0.357 | 0.301 | 0.230 | 0.328 |
|  |  |  |  |  | CV% |  | 72.5 | 72.1 | 54.2 | 45.5 | 40.7 | 38.7 | 37.5 | 36.5 | 35.4 | 34.5 | 34.4 | 34.2 | 34.7 | 31.7 | 25.9 | 23.8 | 25.5 | 28.1 | 30.9 | 59.2 |
|  | | |  | | Min | 0.00 | 0.814 | 2.79 | 5.60 | 13.1 | 14.9 | 18.3 | 18.8 | 19.7 | 17.7 | 16.2 | 14.4 | 12.6 | 11.1 | 4.10 | 1.98 | 1.49 | 0.948 | 0.725 | 0.00 | 0.00 |
|  | | |  | | Max | 0.00 | 13.4 | 53.8 | 74.4 | 87.8 | 94.2 | 97.3 | 99.9 | 95.2 | 90.4 | 82.1 | 75.5 | 65.3 | 59.0 | 18.7 | 5.62 | 3.60 | 2.57 | 1.99 | 1.30 | 1.14 |

Table S3.Plasma concentration of amlodipine (ng/ml) after oral administration of test Lisinopril/amlodipine tablets (lisinopril 10mg / amlodipine 5 mg) to 40 subjects under fasting condition

|  | | | | | | Nominal_Time | | | | | | | | | | | | | | | | | | | | |
| --- | --- | --- | --- | --- | --- | --- | --- | --- | --- | --- | --- | --- | --- | --- | --- | --- | --- | --- | --- | --- | --- | --- | --- | --- | --- | --- |
|  |  |  |  |  |  | (h) | | | | | | | | | | | | | | | | | | | | |
|  |  |  |  |  |  | 0 | 1 | 2 | 3 | 4 | 5 | 6 | 7 | 8 | 9 | 10 | 11 | 12 | 13 | 24 | 36 | 48 | 72 | 96 | 144 | 168 |
| Group | Analyte | Formulation | Subject | Period | Sequence | Concentration | | | | | | | | | | | | | | | | | | | | |
|  |  |  |  |  |  | (ng/ml) | | | | | | | | | | | | | | | | | | | | |
| FAS | Amlodipine | T | FAS001 | 2 | RT | BQL | 0.387 | 0.822 | 1.05 | 1.40 | 1.82 | 2.45 | 2.27 | 2.30 | 1.97 | 1.93 | 1.84 | 1.73 | 1.71 | 1.47 | 1.13 | 0.894 | 0.618 | 0.395 | 0.184 | 0.149 |
|  | | | FAS002 | 2 | RT | BQL | 0.189 | 0.457 | 0.645 | 0.871 | 1.37 | 1.07 | 1.51 | 1.49 | 1.18 | 1.30 | 1.12 | 1.10 | 1.06 | 0.952 | 0.732 | 0.638 | 0.400 | 0.241 | 0.0986 | 0.0570 |
|  | | | FAS003 | 1 | TR | BQL | 0.723 | 1.81 | 2.40 | 2.18 | 2.29 | 2.39 | 2.44 | 2.28 | 2.22 | 2.12 | 1.95 | 1.87 | 1.74 | 1.65 | 1.24 | 1.14 | 0.819 | 0.527 | 0.249 | 0.178 |
|  | | | FAS004 | 1 | TR | BQL | 0.776 | 1.14 | 1.45 | 1.37 | 1.86 | 1.99 | 1.76 | 1.71 | 1.61 | 1.50 | 1.43 | 1.45 | 1.38 | 1.05 | 0.937 | 0.766 | 0.518 | 0.311 | 0.133 | 0.0883 |
|  | | | FAS005 | 2 | RT | BQL | 0.539 | 1.04 | 1.39 | 1.57 | 2.31 | 2.12 | 2.48 | 2.24 | 2.18 | 2.07 | 1.95 | 1.75 | 1.83 | 1.68 | 1.21 | 1.00 | 0.727 | 0.471 | 0.240 | 0.147 |
|  | | | FAS006 | 1 | TR | BQL | 1.31 | 1.27 | 2.25 | 2.24 | 2.24 | 2.45 | 2.59 | 2.45 | 2.48 | 2.16 | 2.08 | 2.26 | 2.14 | 1.59 | 1.36 | 1.02 | 0.729 | 0.532 | 0.269 | 0.161 |
|  | | | FAS007 | 2 | RT | BQL | 0.730 | 1.24 | 1.26 | 1.57 | 1.88 | 2.20 | 2.44 | 2.78 | 2.72 | 2.65 | 2.47 | 2.28 | 2.36 | 1.71 | 1.58 | 1.10 | 0.801 | 0.537 | 0.222 | 0.164 |
|  | | | FAS008 | 1 | TR | BQL | 1.29 | 1.96 | 2.36 | 3.00 | 3.00 | 2.77 | 2.79 | 2.59 | 2.38 | 2.31 | 2.28 | 2.24 | 1.84 | 1.62 | 1.35 | 1.30 | 1.04 | 0.641 | 0.319 | 0.212 |
|  | | | FAS009 | 2 | RT | BQL | 0.338 | 0.697 | 1.02 | 1.21 | 1.31 | 1.27 | 1.58 | 1.54 | 1.44 | 1.38 | 1.42 | 1.53 | 1.43 | 1.04 | 0.838 | 0.673 | 0.482 | 0.319 | 0.137 | 0.0831 |
|  | | | FAS010 | 2 | RT | BQL | 0.454 | 1.32 | 1.22 | 2.74* | 2.32 | 2.61 | 2.52 | 2.78 | 2.85 | 2.47 | 2.96 | 2.29 | 2.33 | 1.74 | 1.28 | 0.852 | 0.557 | 0.375 | 0.152 | 0.102 |
|  | | | FAS011 | 1 | TR | BQL | 0.561 | 1.28 | 1.12 | 1.53 | 1.98 | 2.24 | 1.92 | 2.12 | 2.26 | 2.39 | 2.20 | 2.25 | 2.04 | 1.57 | 1.32 | 1.08 | 0.761 | 0.529 | 0.226 | 0.141 |
|  | | | FAS012 | 1 | TR | BQL | 0.602 | 1.21 | 1.68 | 1.43 | 2.32 | 2.02 | 1.96 | 1.83 | 1.67 | 1.58 | 1.46 | 1.39 | 1.39 | 1.21 | 1.00 | 0.825 | 0.544 | 0.391 | 0.165 | 0.118 |
|  | | | FAS013 | 1 | TR | BQL | 0.270 | 0.642 | 0.866 | 0.991 | 1.27 | 1.66 | 1.85 | 1.64 | 1.65 | 1.66 | 1.74 | 1.56 | 1.58 | 1.35 | 1.16 | 0.976 | 0.664 | 0.454 | 0.188 | 0.140 |
|  | | | FAS014 | 2 | RT | BQL | 0.562 | 1.02 | 0.867 | 0.992 | 1.37 | 1.47 | 1.54 | 1.72 | 1.52 | 1.35 | 1.38 | 1.32 | 1.22 | 1.16 | 0.966 | 0.841 | 0.504 | 0.317 | 0.116 | 0.0696 |
|  | | | FAS015 | 1 | TR | BQL | 0.813 | 1.06 | 1.38 | 1.84 | 2.19 | 1.97 | 1.71 | 1.61 | 1.59 | 1.44 | 1.34 | 1.35 | 1.32 | 0.987 | 0.772 | 0.621 | 0.420 | 0.273 | 0.129 | 0.0805 |
|  | | | FAS016 | 2 | RT | BQL | 1.13 | 2.31 | 2.07 | 1.88 | 2.20 | 2.24 | 1.97 | 1.79 | 1.59 | 1.48 | 1.45 | 1.31 | 1.24 | 1.01 | 0.818 | 0.616 | 0.448 | 0.285 | 0.151 | 0.107 |
|  | | | FAS017 | 1 | TR | BQL | 1.33 | 1.60 | 1.48 | 1.40 | 1.61 | 1.59 | 1.48 | 1.47 | 1.23 | 1.10 | 1.10 | 1.08 | 0.973 | 0.756 | 0.619 | 0.502 | 0.383 | 0.329 | 0.136 | 0.0894 |
|  | | | FAS018 | 1 | TR | BQL | 0.761 | 1.35 | 1.47 | 1.67 | 2.17 | 2.44 | 2.27 | 2.42 | 2.70 | 1.78 | 2.16 | 2.36 | 2.65 | 1.87 | 1.50 | 1.26 | 0.845 | 0.564 | 0.277 | 0.196 |
|  | | | FAS019 | 2 | RT | 0.0683 | 0.385 | 0.901 | 1.25 | 2.04 | 2.66 | 3.58 | 3.60 | 4.20 | 3.45 | 3.27 | 3.13 | 3.08 | 2.67 | 2.65 | 2.40 | 1.89 | 1.56 | 1.08 | 0.560 | 0.428 |
|  | | | FAS020 | 2 | RT | BQL | 0.788 | 1.20 | 1.29 | 1.79 | 2.04 | 2.13 | 2.13 | 2.31 | 2.44 | 2.21 | 2.00 | 1.85 | 1.71 | 1.49 | 1.25 | 0.933 | 0.522 | 0.348 | 0.124 | 0.0902 |
|  | | | FAS021 | 1 | TR | BQL | 0.209 | 0.774 | 1.19 | 1.02 | 1.42 | 1.30 | 1.42 | 1.22 | 1.39 | 1.37 | 1.20 | 1.25 | 1.30 | 1.02 | 0.717 | 0.589 | 0.345 | 0.220 | 0.0879 | 0.0567 |
|  | | | FAS022 | 1 | TR | BQL | 0.764 | 1.07 | 1.22 | 1.36 | 1.76 | 1.55 | 1.54 | 1.52 | 1.43 | 1.30 | 1.31 | 1.29 | 1.17 | 0.992 | 0.785 | 0.591 | 0.417 | 0.225 | 0.0943 | 0.0594 |
|  | | | FAS023 | 2 | RT | BQL | 0.511 | 0.841 | 1.06 | 1.92 | 2.77 | 2.78 | 2.89 | 3.24 | 3.17 | 3.11 | 3.07 | 2.91 | 2.71 | 2.28 | 1.83 | 1.68 | 1.12 | 0.748* | 0.407 | 0.366 |
|  | | | FAS024 | 2 | RT | BQL | 0.493 | 1.05 | 1.12 | 1.65 | 2.23 | 2.68 | 2.08 | 2.17 | 2.24 | 2.17 | 2.30 | 2.13 | 2.25 | 1.90 | 1.30 | 1.11 | 0.842 | 0.566 | 0.233 | 0.160 |
|  | | | FAS025 | 2 | RT | BQL | 0.293 | 0.975 | 1.01 | 1.29 | 1.43 | 1.59 | 1.69 | 1.79 | 1.72 | 1.58 | 1.64 | 1.41 | 1.39 | 1.34 | 0.950 | 0.835 | 0.591 | 0.461 | 0.208 | 0.164 |
|  | | | FAS026 | 2 | RT | BQL | 0.889 | 1.56 | 0.876 | 1.19 | 1.30 | 1.59 | 1.79 | 1.63 | 1.51 | 1.43 | 1.23 | 1.36 | 1.36 | 1.17 | 0.843 | 0.778 | 0.556 | 0.355 | 0.152 | 0.108 |
|  | | | FAS027 | 1 | TR | BQL | 0.621 | 1.08 | 1.11 | 1.30 | 2.43 | 1.98 | 2.08 | 1.93 | 1.82 | 1.81 | 1.81 | 1.60 | 1.53 | 1.37 | 1.08 | 0.944 | 0.592 | 0.437 | 0.161 | 0.108 |
|  | | | FAS028 | 1 | TR | BQL | 0.826 | 2.54 | 2.77 | 1.86 | 2.09 | 2.18 | 2.32 | 2.33 | 2.31 | 2.44 | 2.26 | 2.28 | 2.27 | 1.58 | 1.51 | 1.20 | 0.828 | 0.584 | 0.311 | 0.254 |
|  | | | FAS029 | 2 | RT | BQL | 0.232 | 0.566 | 0.746 | 0.857 | 1.15 | 0.925 | 1.08 | 0.924 | 0.829 | 0.750 | 0.766 | 0.739 | 0.713 | 0.622 | 0.460 | 0.300 | 0.172 | 0.0991 | BQL | BQL |
|  | | | FAS030 | 1 | TR | BQL | 0.798 | 1.33 | 1.42 | 1.63 | 2.23 | 2.09 | 2.07 | 2.05 | 2.00 | 1.75 | 1.78 | 1.71 | 1.53 | 1.29 | 1.12 | 0.860 | 0.579 | 0.332 | 0.147 | 0.0954 |
|  | | | FAS031 | 1 | TR | BQL | 0.196 | 0.986 | 0.946 | 1.38 | 2.52 | 2.26 | 2.32 | 2.41 | 2.44 | 2.09 | 2.04 | 1.96 | 1.96 | 1.48 | 1.31 | 1.18 | 0.889 | 0.537 | 0.312 | 0.229 |
|  | | | FAS032 | 2 | RT | BQL | 0.287 | 0.653 | 0.990 | 1.28 | 1.64 | 1.78 | 1.92 | 2.11 | 1.96 | 1.98 | 1.81 | 1.89 | 1.71 | 1.81 | 1.38 | 1.20 | 1.05 | 0.695 | 0.342 | 0.269 |
|  | | | FAS033 | 2 | RT | BQL | 1.13 | 1.89 | 1.98 | 1.64 | 1.45 | 1.82 | 1.69 | 1.82 | 1.92 | 1.69 | 1.72 | 1.72 | 1.74 | 1.28 | 0.952 | 0.789 | 0.571 | 0.363 | 0.164 | 0.109 |
|  | | | FAS034 | 1 | TR | BQL | 0.636 | 1.19 | 1.42 | 1.52 | 1.83 | 1.99 | 1.78 | 2.23 | 2.04 | 1.83 | 1.83 | 2.04 | 1.87 | 1.54 | 1.18 | 1.01 | 0.714 | 0.476 | 0.218 | 0.136 |
|  | | | FAS035 | 2 | RT | BQL | 0.789 | 1.21 | 1.39 | 1.61 | 1.64 | 2.02 | 2.70 | 2.28 | 2.11 | 2.06 | 1.76 | 1.83 | 1.81 | 1.38 | 1.13 | 0.957 | 0.601 | 0.427 | 0.227 | 0.156 |
|  | | | FAS036 | 1 | TR | BQL | 0.638 | 0.846 | 1.15 | 1.38 | 1.50 | 1.73 | 1.78 | 1.69 | 1.51 | 1.36 | 1.25 | 1.33 | 1.21 | 0.968 | 0.747 | 0.587 | 0.394 | 0.238 | 0.113 | 0.0740 |
|  | | | FAS037 | 1 | TR | BQL | 0.181 | 0.424 | 0.680 | 0.947 | 1.31 | 2.08 | 1.34 | 1.51 | 1.48 | 1.27 | 1.22 | 1.19 | 1.06 | 0.961 | 0.747 | 0.588 | 0.452 | 0.358 | 0.157 | 0.0954 |
|  | | | FAS038 | 1 | TR | BQL | 0.324 | 1.07 | 1.85 | 2.23 | 1.99 | 2.32 | 2.35* | 2.59 | 2.47 | 2.50 | 2.43 | 2.40 | 2.18 | 1.80 | 1.70 | 1.32 | 0.979 | 0.777 | 0.416 | 0.306 |
|  | | | FAS039 | 2 | RT | BQL | 0.643 | 0.584 | 1.08 | 1.11 | 1.95 | 2.15 | 2.08 | 1.89 | 1.80 | 1.78 | 1.67 | 1.61 | 1.55 | 1.37 | 1.15 | 0.847 | 0.654 | 0.364 | 0.162 | 0.118 |
|  | | | FAS040 | 2 | RT | BQL | 0.475 | 0.860 | 2.21 | 2.12 | 2.42 | 3.03 | 2.95 | 2.65 | 2.57 | 2.61 | 2.35 | 2.33 | 2.28 | 1.76 | 1.43 | 1.05 | 0.747 | 0.467 | 0.212 | 0.118 |
|  | | |  | | N | 40 | 40 | 40 | 40 | 39 | 40 | 40 | 39 | 40 | 40 | 40 | 40 | 40 | 40 | 40 | 40 | 40 | 40 | 39 | 40 | 40 |
|  | | |  | | Mean | 0.00171 | 0.622 | 1.15 | 1.37 | 1.55 | 1.93 | 2.06 | 2.06 | 2.08 | 2.00 | 1.88 | 1.82 | 1.78 | 1.71 | 1.41 | 1.14 | 0.934 | 0.661 | 0.433 | 0.205 | 0.145 |
|  | | |  | | SD | 0.0108 | 0.315 | 0.467 | 0.508 | 0.446 | 0.464 | 0.529 | 0.516 | 0.587 | 0.562 | 0.543 | 0.546 | 0.510 | 0.494 | 0.407 | 0.369 | 0.311 | 0.256 | 0.176 | 0.104 | 0.0855 |
|  |  |  |  |  | CV% | 632.5 | 50.6 | 40.8 | 37.1 | 28.9 | 24.0 | 25.6 | 25.0 | 28.2 | 28.2 | 29.0 | 29.9 | 28.7 | 29.0 | 28.8 | 32.2 | 33.3 | 38.8 | 40.7 | 50.9 | 59.1 |
|  | | |  | | Min | 0.00 | 0.181 | 0.424 | 0.645 | 0.857 | 1.15 | 0.925 | 1.08 | 0.924 | 0.829 | 0.750 | 0.766 | 0.739 | 0.713 | 0.622 | 0.460 | 0.300 | 0.172 | 0.0991 | 0.00 | 0.00 |
|  | | |  | | Max | 0.0683 | 1.33 | 2.54 | 2.77 | 3.00 | 3.00 | 3.58 | 3.60 | 4.20 | 3.45 | 3.27 | 3.13 | 3.08 | 2.71 | 2.65 | 2.40 | 1.89 | 1.56 | 1.08 | 0.560 | 0.428 |

Table S4. Plasma concentration of amlodipine (ng/ml) after oral administration of reference Lisinopril/amlodipine tablets (lisinopril 10mg / amlodipine 5 mg) to 39 subjects under fasting condition

|  | | | | | | Nominal_Time | | | | | | | | | | | | | | | | | | | | |
| --- | --- | --- | --- | --- | --- | --- | --- | --- | --- | --- | --- | --- | --- | --- | --- | --- | --- | --- | --- | --- | --- | --- | --- | --- | --- | --- |
|  |  |  |  |  |  | (h) | | | | | | | | | | | | | | | | | | | | |
|  |  |  |  |  |  | 0 | 1 | 2 | 3 | 4 | 5 | 6 | 7 | 8 | 9 | 10 | 11 | 12 | 13 | 24 | 36 | 48 | 72 | 96 | 144 | 168 |
| Group | Analyte | Formulation | Subject | Period | Sequence | Concentration | | | | | | | | | | | | | | | | | | | | |
|  |  |  |  |  |  | (ng/ml) | | | | | | | | | | | | | | | | | | | | |
| FAS | Amlodipine | R | FAS001 | 1 | RT | BQL | 0.280 | 0.785 | 1.85 | 1.68 | 1.74 | 2.95 | 2.67 | 2.66 | 2.36 | 2.02 | 2.04 | 1.84 | 1.77 | 1.49 | 1.12 | 0.943 | 0.578 | 0.442 | 0.209 | 0.102 |
|  | | | FAS002 | 1 | RT | BQL | 0.450 | 0.857 | 1.01 | 0.834 | 1.26 | 1.25 | 1.03 | 0.988 | 0.918 | 0.902 | 0.864 | 0.831 | 0.842 | 0.750 | 0.596 | 0.453 | 0.328 | 0.184 | 0.0835 | 0.0581 |
|  | | | FAS003 | 2 | TR | BQL | 0.288 | 0.762 | 1.42 | 1.55 | 2.43 | 2.08 | 2.41 | 2.38 | 2.45 | 2.43 | 2.22 | 2.11 | 1.95 | 1.81 | 1.41 | 1.19 | 0.813 | 0.574 | 0.299 | 0.170 |
|  | | | FAS004 | 2 | TR | BQL | 0.519 | 0.852 | 1.32 | 1.32 | 1.66 | 1.60 | 1.60 | 1.47 | 1.49 | 1.40 | 1.38 | 1.26 | 1.12 | 1.16 | 0.772 | 0.611 | 0.409 | 0.241 | 0.0914 | 0.0620 |
|  | | | FAS005 | 1 | RT | BQL | 0.340 | 1.10 | 1.77 | 1.39 | 2.17 | 2.08 | 1.91 | 2.02 | 2.02 | 1.82 | 1.83 | 1.95 | 1.84 | 1.44 | 1.17 | 0.977 | 0.650 | 0.436 | 0.176 | 0.129 |
|  | | | FAS006 | 2 | TR | BQL | 0.720 | 2.15 | 2.06 | 2.01 | 2.13 | 2.74 | 2.50 | 2.33 | 2.32 | 2.19 | 2.07 | 2.12 | 1.89 | 1.72 | 1.62 | 1.10 | 0.870 | 0.590 | 0.327 | 0.249 |
|  | | | FAS007 | 1 | RT | BQL | 0.724 | 1.15 | 2.12 | 1.57 | 2.19 | 2.46 | 2.36 | 2.44 | 2.14 | 2.13 | 2.23 | 2.21 | 2.00 | 1.59 | 1.47 | 1.13 | 0.770 | 0.555 | 0.246 | 0.174 |
|  | | | FAS008 | 2 | TR | BQL | 1.20 | 2.02 | 2.10 | 2.39 | 2.98 | 2.84 | 2.67 | 2.76 | 2.75 | 2.52 | 2.29 | 2.12 | 2.02 | 1.86 | 1.45 | 1.37 | 0.896 | 0.652 | 0.275 | 0.221 |
|  | | | FAS009 | 1 | RT | BQL | 0.265 | 0.452 | 0.598 | 1.03 | 1.40 | 1.66 | 1.42 | 1.41 | 1.32 | 1.18 | 1.20 | 1.33 | 1.20 | 1.08 | 0.860 | 0.729 | 0.486 | 0.306 | 0.142 | 0.104 |
|  | | | FAS010 | 1 | RT | BQL | 0.608 | 1.79 | 1.69 | 1.87 | 2.23 | 3.20 | 2.59* | 2.71 | 2.40 | 2.28 | 2.21 | 2.06 | 2.11 | 1.53 | 1.24 | 0.825 | 0.460 | 0.315 | 0.102 | 0.0535 |
|  | | | FAS011 | 2 | TR | BQL | 0.580 | 1.39 | 1.64 | 1.64 | 2.53 | 2.27 | 2.39 | 2.53 | 2.25 | 2.27 | 2.10 | 2.36 | 2.22 | 1.79 | 1.50 | 1.23 | 0.805 | 0.644 | 0.266 | 0.164 |
|  | | | FAS012 | 2 | TR | BQL | 0.646 | 0.740 | 1.36 | 1.45 | 1.95 | 2.12 | 2.14 | 2.22 | 1.96 | 1.78 | 1.68 | 1.44 | 1.49 | 1.37 | 0.885 | 0.841 | 0.649 | 0.418 | 0.201 | 0.142* |
|  | | | FAS013 | 2 | TR | BQL | 0.477 | 0.965 | 1.24 | 1.36 | 1.82 | 1.67 | 1.77 | 1.82 | 2.14 | 1.82 | 1.79 | 1.70 | 1.64 | 1.34 | 1.19 | 0.831 | 0.635 | 0.404 | 0.164 | 0.122 |
|  | | | FAS014 | 1 | RT | BQL | 0.470 | 0.959 | 1.34 | 1.15 | 1.63 | 1.61 | 1.47 | 1.42 | 1.27 | 1.18 | 1.24 | 1.33 | 1.20 | 0.985 | 0.775 | 0.595 | 0.416 | 0.262 | 0.0977 | 0.0634 |
|  | | | FAS015 | 2 | TR | BQL | 0.554 | 1.05 | 1.33 | 1.21 | 2.37 | 1.64 | 1.86 | 1.82 | 1.90 | 1.84 | 1.71 | 1.66 | 1.60 | 1.43 | 1.16 | 0.952 | 0.575 | 0.389 | 0.173 | 0.0923 |
|  | | | FAS016 | 1 | RT | BQL | 2.24 | 2.57 | 3.08 | 2.44 | 2.45 | 2.33 | 1.99 | 1.92 | 1.73 | 1.51 | 1.43 | 1.42 | 1.33 | 1.06 | 0.837 | 0.760 | 0.479 | 0.371 | 0.153 | 0.120 |
|  | | | FAS017 | 2 | TR | BQL | 0.579 | 1.30 | 1.92 | 1.84 | 1.75 | 2.03 | 2.04 | 1.80 | 1.71 | 1.56 | 1.43 | 1.38 | 1.37 | 1.03 | 0.747 | 0.649 | 0.481 | 0.348 | 0.177 | 0.102 |
|  | | | FAS018 | 2 | TR | BQL | 0.434 | 0.920 | 1.02 | 1.52 | 1.87 | 2.06 | 1.93 | 2.44 | 2.21 | 2.47 | 2.10 | 2.19 | 2.41 | 1.62 | 1.42 | 1.12 | 0.791 | 0.594 | 0.222 | 0.166 |
|  | | | FAS019 | 1 | RT | BQL | 0.886 | 1.71 | 1.48 | 2.42 | 2.96 | 4.18 | 2.53 | 3.48 | 3.04 | 2.86 | 2.92 | 2.98 | 2.75 | 2.45 | 1.96 | 1.69 | 1.21 | 1.11 | 0.574 | 0.455 |
|  | | | FAS020 | 1 | RT | BQL | 0.887 | 1.89 | 2.54 | 2.26 | 2.66 | 2.63 | 2.29 | 2.34 | 2.11 | 1.91 | 1.91 | 1.96 | 1.87 | 1.55 | 1.30 | 0.895 | 0.595 | 0.393 | 0.146 | 0.0915 |
|  | | | FAS021 | 2 | TR | BQL | 0.700 | 0.969 | 0.915 | 1.41 | 1.46 | 1.43 | 1.50 | 1.45 | 1.55 | 1.45 | 1.26 | 1.19 | 1.16 | 0.982 | 0.754 | 0.654 | 0.411 | 0.236 | 0.108 | 0.0628 |
|  | | | FAS022 | 2 | TR | BQL | 0.833 | 1.50 | 1.37 | 1.72 | 1.82 | 1.73 | 1.65 | 1.57 | 1.50 | 1.43 | 1.39 | 1.25 | 1.30 | 1.03 | 0.750 | 0.623 | 0.395 | 0.237 | 0.0889 | 0.0521 |
|  | | | FAS023 | 1 | RT | BQL | 0.426 | 0.800 | 0.866 | 1.41 | 2.18 | 2.76 | 2.23 | 2.74 | 2.78 | 2.68 | 2.62 | 2.37 | 2.42 | 1.90 | 1.58 | 1.24 | 0.944 | 0.682 | 0.332 | 0.231 |
|  | | | FAS024 | 1 | RT | BQL | 0.587 | 1.22 | 1.59 | 1.66 | 2.49 | 2.30 | 2.19 | 1.91 | 1.80 | 1.58 | 1.54 | 1.57 | 1.46 | 1.23 | 0.934 | 0.781 | 0.548 | 0.322 | 0.151 | 0.0973 |
|  | | | FAS025 | 1 | RT | BQL | 0.353 | 1.11 | 0.927 | 1.18 | 1.33 | 1.45 | 1.57 | 1.28 | 1.30 | 1.36 | 1.07 | 1.18 | 1.05 | 0.903 | 0.702 | 0.637 | 0.428 | 0.342 | 0.145 | 0.0969 |
|  | | | FAS026 | 1 | RT | BQL | 1.05 | 1.34 | 1.23 | 0.835 | 1.37 | 1.66 | 1.49 | 1.46 | 1.21 | 1.18 | 1.15 | 1.06 | 1.14 | 0.924 | 0.769 | 0.675 | 0.468 | 0.352 | 0.125 | 0.0893 |
|  | | | FAS027 | 2 | TR | BQL | 0.528 | 1.08 | 1.29 | 1.43 | 2.46 | 2.51 | 2.36 | 2.53 | 2.23 | 2.02 | 2.17 | 1.92 | 1.89 | 1.50 | 1.22 | 0.973 | 0.646 | 0.398 | 0.162 | 0.0960 |
|  | | | FAS028 | 2 | TR | BQL | 1.28 | 1.20 | 2.06 | 2.02 | 2.52 | 2.44 | 2.70 | 2.40 | 2.37 | 2.29 | 2.16 | 2.15 | 1.99 | 1.65 | 1.40 | 1.17 | 0.826 | 0.579 | 0.311 | 0.204 |
|  | | | FAS029 | 1 | RT | BQL | 0.347 | 0.663 | 0.980 | 0.951 | 1.30 | 1.14 | 1.16 | 1.14 | 0.933 | 0.806 | 0.820 | 0.829 | 0.812 | 0.603 | 0.482 | 0.391 | 0.226 | 0.123 | BQL | BQL |
|  | | | FAS030 | 2 | TR | BQL | 0.828 | 1.38 | 1.23 | 1.41 | 1.83 | 2.13 | 2.17 | 1.99 | 1.91 | 1.74 | 1.68 | 1.76 | 1.55 | 1.37 | 1.10 | 0.772 | 0.532 | 0.385 | 0.162 | 0.0941 |
|  | | | FAS031 | 2 | TR | BQL | 0.710 | 1.81 | 1.37 | 1.44 | 2.09 | 2.28 | 2.33 | 2.32 | 2.18 | 2.10 | 2.29 | 2.34 | 2.22 | 1.93 | 1.68 | 1.38 | 1.06 | 0.753 | 0.320 | 0.234 |
|  | | | FAS032 | 1 | RT | BQL | 0.692 | 1.30 | 1.78 | 2.29 | 2.30 | 2.50 | 2.08 | 1.98 | 2.05 | 1.95 | 1.86 | 1.68 | 1.82 | 1.74 | 1.36 | 1.17 | 0.853 | 0.643 | 0.361 | 0.267 |
|  | | | FAS033 | 1 | RT | BQL | 1.16 | 2.07 | 1.98 | 1.88 | 2.13 | 2.09 | 2.11 | 2.10 | 1.82 | 1.73 | 1.52 | 1.52 | 1.43 | 1.37 | 1.12 | 0.850 | 0.577 | 0.371 | 0.167 | 0.106 |
|  | | | FAS034 | 2 | TR | BQL | 0.834 | 1.25 | 1.45 | 1.76 | 2.01 | 1.93 | 2.59 | 2.64 | 2.44 | 2.39 | 2.14 | 2.08 | 1.87 | 1.76 | 1.25 | 0.974 | 0.651 | 0.453 | 0.197 | 0.142 |
|  | | | FAS035 | 1 | RT | BQL | 0.941 | 1.79 | 1.68 | 2.20 | 2.25 | 2.12 | 1.94 | 1.83 | 1.59 | 1.69 | 1.45 | 1.58 | 1.41 | 1.38 | 0.967 | 0.844 | 0.619 | 0.394 | 0.189 | 0.134 |
|  | | | FAS036 | 2 | TR | BQL | 0.820 | 1.14 | 1.19 | 1.34 | 2.12 | 2.36 | 2.18 | 2.48 | 2.11 | 2.00 | 1.92 | 1.84 | 1.65 | 1.46 | 1.16 | 0.984 | 0.626 | 0.487 | 0.197 | 0.173 |
|  | | | FAS037 | 2 | TR | BQL | 0.260 | 0.519 | 0.862 | 0.877 | 1.59 | 1.60 | 1.49 | 1.60 | 1.51 | 1.33 | 1.30 | 1.30 | 1.30 | 1.03 | 0.853 | 0.683 | 0.580 | 0.373 | 0.194 | 0.142 |
|  | | | FAS039 | 1 | RT | BQL | 0.372 | 0.851 | 1.09 | 1.38 | 1.68 | 1.84 | 1.89 | 1.94 | 1.84 | 1.72 | 1.80 | 1.61 | 1.71 | 1.48 | 1.11 | 0.989 | 0.792 | 0.459 | 0.197 | 0.149 |
|  | | | FAS040 | 1 | RT | BQL | 0.892 | 1.13 | 2.09 | 2.45 | 2.25 | 2.57 | 2.76 | 2.62 | 2.70 | 2.56 | 2.43 | 2.45 | 2.56* | 1.79 | 1.22 | 1.52 | 0.865 | 0.561 | 0.253 | 0.151 |
|  | | |  | | N | 39 | 39 | 39 | 39 | 39 | 39 | 39 | 38 | 39 | 39 | 39 | 39 | 39 | 38 | 39 | 39 | 39 | 39 | 39 | 39 | 38 |
|  | | |  | | Mean | 0.00 | 0.686 | 1.24 | 1.51 | 1.60 | 2.03 | 2.16 | 2.04 | 2.08 | 1.96 | 1.85 | 1.77 | 1.74 | 1.65 | 1.41 | 1.13 | 0.928 | 0.640 | 0.446 | 0.200 | 0.137 |
|  | | |  | | SD | 0.00 | 0.369 | 0.483 | 0.507 | 0.461 | 0.445 | 0.587 | 0.446 | 0.539 | 0.504 | 0.495 | 0.486 | 0.480 | 0.450 | 0.380 | 0.333 | 0.288 | 0.210 | 0.183 | 0.101 | 0.0805 |
|  |  |  |  |  | CV% |  | 53.8 | 38.8 | 33.6 | 28.7 | 21.9 | 27.2 | 21.9 | 26.0 | 25.8 | 26.8 | 27.4 | 27.6 | 27.3 | 26.9 | 29.6 | 31.1 | 32.8 | 41.0 | 50.4 | 58.6 |
|  | | |  | | Min | 0.00 | 0.260 | 0.452 | 0.598 | 0.834 | 1.26 | 1.14 | 1.03 | 0.988 | 0.918 | 0.806 | 0.820 | 0.829 | 0.812 | 0.603 | 0.482 | 0.391 | 0.226 | 0.123 | 0.00 | 0.00 |
|  | | |  | | Max | 0.00 | 2.24 | 2.57 | 3.08 | 2.45 | 2.98 | 4.18 | 2.76 | 3.48 | 3.04 | 2.86 | 2.92 | 2.98 | 2.75 | 2.45 | 1.96 | 1.69 | 1.21 | 1.11 | 0.574 | 0.455 |

Table S5.Plasma concentration of lisinopril (ng/ml) after oral administration of test Lisinopril/amlodipine tablets (lisinopril 10mg / amlodipine 5 mg) to 38 subjects under fed condition

|  | | | | | | Group | | | | | | | | | | | | | | | | | | | | |
| --- | --- | --- | --- | --- | --- | --- | --- | --- | --- | --- | --- | --- | --- | --- | --- | --- | --- | --- | --- | --- | --- | --- | --- | --- | --- | --- |
|  |  |  |  |  |  | FED | | | | | | | | | | | | | | | | | | | | |
|  |  |  |  |  |  | Nominal_Time | | | | | | | | | | | | | | | | | | | | |
|  |  |  |  |  |  | (h) | | | | | | | | | | | | | | | | | | | | |
|  |  |  |  |  |  | 0 | 1 | 2 | 3 | 4 | 5 | 6 | 7 | 8 | 9 | 10 | 11 | 12 | 13 | 24 | 36 | 48 | 72 | 96 | 144 | 168 |
| Analyte | Formulation | Subject | Period | Sequence | Stage | Concentration | | | | | | | | | | | | | | | | | | | | |
|  |  |  |  |  |  | (ng/ml) | | | | | | | | | | | | | | | | | | | | |
| LSN | T | FED001 | 2 | RT | 1 | BQL | BQL | 1.74 | 4.03 | 13.9 | 16.5 | 19.4 | 17.7 | 16.6 | 15.3 | 13.8 | 11.5 | 9.71 | 8.68 | 3.39 | 1.73 | 1.36 | 0.900 | 0.789 | 0.590 | 0.563 |
|  | | FED002 | 2 | RT | 1 | BQL | BQL | 2.61 | 9.24 | 16.5 | 17.9 | 18.7 | 18.0 | 17.8 | 16.1 | 16.4 | 13.6 | 12.2 | 11.2 | 3.85 | 2.08 | 1.59 | 1.50 | 1.02 | 0.507 | BQL |
|  | | FED003 | 1 | TR | 1 | BQL | BQL | 0.851 | 2.84 | 6.11 | 9.13 | 11.6 | 12.6 | 13.4 | 13.7 | 11.8 | 11.3 | 9.99 | 9.03 | 3.13 | 1.38 | 1.05 | 0.746 | 0.618 | BQL | BQL |
|  | | FED004 | 1 | TR | 1 | BQL | BQL | 1.19 | 3.08 | 5.90 | 9.90 | 10.7 | 11.1 | 12.5 | 12.5 | 11.9 | 10.6 | 9.79 | 9.02 | 4.38 | 2.94 | 2.24 | 1.71 | 1.32 | 0.932 | 0.891 |
|  | | FED005 | 2 | RT | 1 | BQL | 1.10 | 6.10 | 13.1 | 15.5 | 15.3 | 15.4 | 14.0 | 13.5 | 12.0 | 11.8 | 10.1 | 8.82 | 8.75 | 4.01 | 2.39 | 1.87 | 1.52 | 1.22 | 0.901 | 0.790 |
|  | | FED006 | 1 | TR | 1 | BQL | 0.698 | 1.46 | 2.68 | 2.94 | 4.01 | 5.11 | 5.33 | 6.38 | 6.32 | 6.64 | 6.37 | 6.05 | 5.70 | 3.58 | 2.25 | 1.92 | 1.80 | 1.47 | 0.914 | 0.770 |
|  | | FED007 | 2 | RT | 1 | BQL | BQL | 2.13 | 5.68 | 11.1 | 13.5 | 14.7 | 15.3 | 14.8 | 14.6 | 14.1 | 12.7 | 11.6 | 10.9 | 4.44 | 2.06 | 1.72 | 2.72 | 1.01 | 0.726 | 0.592 |
|  | | FED008 | 1 | TR | 1 | BQL | BQL | 1.94 | 5.29 | 6.19 | 9.08 | 9.98 | 10.1 | 9.85 | 9.49 | 8.93 | 7.69 | 7.43 | 7.02 | 3.32 | 1.84 | 1.71 | 1.74 | 1.30 | 0.733 | 0.707 |
|  | | FED009 | 2 | RT | 1 | BQL | 0.969 | 2.72 | 4.95 | 7.84 | 9.71 | 10.1 | 10.9 | 11.0 | 10.1 | 9.74 | 9.15 | 8.36 | 7.32 | 3.55 | 2.04 | 1.46 | 1.02 | 0.862 | 0.579 | BQL |
|  | | FED010 | 2 | RT | 1 | BQL | BQL | BQL | 1.18 | 1.56 | 3.59 | 6.14 | 7.26 | 7.80 | 7.86 | 8.01 | 7.75 | 6.73 | 6.21 | 2.69 | 1.44 | 1.19 | 0.814 | 0.847 | 0.577 | BQL |
|  | | FED011 | 1 | TR | 1 | BQL | 1.77 | 4.87 | 10.3 | 11.8 | 12.6 | 12.9 | 12.9 | 12.2 | 11.1 | 10.8 | 9.41 | 8.15 | 7.71 | 3.67 | 1.97 | 1.63 | 1.08 | 0.919 | 0.649 | 0.582 |
|  | | FED012 | 1 | TR | 1 | BQL | BQL | 1.01 | 2.25 | 5.73 | 10.5 | 12.2 | 13.7 | 12.9 | 12.1 | 12.4 | 11.4 | 10.0 | 8.49 | 3.15 | 1.51 | 1.09 | 0.657 | 0.585 | BQL | BQL |
|  | | FED026 | 2 | RT | 1 | BQL | 1.31 | 4.31 | 5.73 | 10.7 | 11.6 | 13.1 | 13.1 | 12.5 | 11.5 | 9.89 | 9.31 | 8.41 | 7.95 | 4.36 | 2.76 | 2.83 | 1.86 | 1.31 | 0.850 | 0.796 |
|  | | FED027 | 1 | TR | 1 | BQL | 1.96 | 4.36 | 13.5 | 19.3 | 21.3 | 22.1 | 21.3 | 19.9 | 19.1 | 17.1 | 16.3 | 13.7 | 13.1 | 5.35 | 2.61 | 1.84 | 1.20 | 1.04 | 0.701 | 0.634 |
|  | | FED028 | 1 | TR | 1 | BQL | 0.688 | 2.45 | 2.45 | 5.08 | 6.49 | 7.18 | 7.91 | 8.14 | 8.26 | 7.79 | 7.20 | 6.94 | 5.85 | 3.31 | 1.82 | 1.71 | 1.42 | 1.21 | 0.682 | 0.670 |
|  | | FED029 | 2 | RT | 1 | BQL | BQL | 1.31 | 2.44 | 5.16 | 7.50 | 9.45 | 10.8 | 10.7 | 9.91 | 10.5 | 9.12 | 8.27 | 7.25 | 2.58 | 1.48 | 1.17 | 0.950 | 0.833 | 0.587 | BQL |
|  | | FED030 | 1 | TR | 1 | BQL | 1.32 | 4.73 | 10.2 | 14.7 | 16.3 | 17.0 | 17.1 | 16.7 | 15.1 | 14.1 | 12.4 | 10.8 | 9.81 | 4.00 | 1.87 | 1.46 | 1.00 | 0.723 | BQL | BQL |
|  | | FED031 | 1 | TR | 1 | BQL | BQL | 0.834 | 1.34 | 3.80 | 7.20 | 9.56 | 10.7 | 11.1 | 10.9 | 10.3 | 9.80 | 8.54 | 7.78 | 3.31 | 1.63 | 1.45 | 0.971 | 0.803 | 0.526 | 0.527 |
|  | | FED032 | 2 | RT | 1 | BQL | 1.83 | 4.23 | 11.2 | 15.2 | 16.3 | 17.3 | 16.7 | 16.3 | 14.7 | 14.6 | 12.9 | 11.5 | 10.9 | 4.49 | 2.38 | 1.94 | 1.31 | 1.10 | 0.777 | 0.740 |
|  | | FED033 | 2 | RT | 1 | BQL | 0.926 | 2.13 | 3.45 | 9.45 | 11.4 | 14.1 | 15.4 | 14.9 | 13.9 | 13.8 | 11.8 | 11.4 | 11.0 | 5.11 | 3.31 | 2.70 | 1.80 | 1.45 | 1.06 | 0.918 |
|  | | FED034 | 1 | TR | 1 | BQL | 1.91 | 3.39 | 6.52 | 11.8 | 16.2 | 16.4 | 17.4 | 16.4 | 15.6 | 14.9 | 13.1 | 11.7 | 10.2 | 4.77 | 2.66 | 2.15 | 1.59 | 1.32 | 0.964 | 0.910 |
|  | | FED035 | 2 | RT | 1 | BQL | 2.88 | 9.01 | 17.2 | 23.9 | 26.4 | 28.0 | 27.9 | 26.1 | 25.3 | 24.3 | 21.4 | 20.3 | 18.2 | 8.21 | 3.52 | 2.85 | 1.98 | 1.61 | 1.22 | 1.09 |
|  | | FED036 | 1 | TR | 1 | BQL | 0.997 | 2.11 | 2.87 | 5.76 | 8.10 | 9.29 | 9.72 | 10.4 | 10.1 | 10.3 | 9.47 | 9.11 | 8.54 | 4.59 | 2.78 | 2.27 | 1.64 | 1.29 | 0.968 | 0.818 |
|  | | FED037 | 1 | TR | 1 | BQL | 1.68 | 5.54 | 12.7 | 18.1 | 20.8 | 21.1 | 19.2 | 18.4 | 16.8 | 15.9 | 14.4 | 13.0 | 11.5 | 4.56 | 2.27 | 1.80 | 1.26 | 0.866 | 0.594 | BQL |
|  | | FED038 | 1 | TR | 1 | BQL | BQL | 0.663 | 1.34 | 1.89 | 3.76 | 6.96 | 9.14 | 10.7 | 12.2 | 12.7 | 10.8 | 9.83 | 9.39 | 3.93 | 2.07 | 1.65 | 1.11 | 0.991 | 0.798 | 0.606 |
|  | | FED039 | 2 | RT | 1 | BQL | BQL | 1.30 | 2.79 | 7.52 | 10.6 | 13.0 | 15.0 | 16.0 | 16.1 | 14.8 | 13.6 | 12.4 | 11.5 | 5.46 | 2.69 | 2.05 | 1.38 | 1.13 | 0.841 | 0.754 |
|  | | FED040 | 2 | RT | 1 | 0.615 | 2.13 | 5.22 | 8.66 | 10.9 | 12.1 | 12.2 | 13.4 | 13.4 | 13.2 | 12.1 | 10.3 | 9.94 | 8.35 | 4.36 | 2.53 | 2.07 | 1.73 | 1.54 | 1.42 | 1.47 |
|  | | FED301 | 2 | RT | 2 | BQL | 0.546 | 2.00 | 5.73 | 10.9 | 12.5 | 14.8 | 14.7 | 15.0 | 14.3 | 13.2 | 12.5 | 10.5 | 9.64 | 3.73 | 2.06 | 1.50 | 1.01 | 0.818 | 0.618 | 0.547 |
|  | | FED302 | 2 | RT | 2 | BQL | BQL | 0.879 | 1.35 | 1.88 | 2.64 | 3.15 | 3.64 | 4.14 | 4.22 | 4.17 | 4.34 | 4.06 | 3.81 | 2.75 | 1.92 | 1.67 | 1.74 | 1.52 | 1.11 | 0.887 |
|  | | FED303 | 1 | TR | 2 | BQL | BQL | 2.20 | 2.27 | 3.52 | 5.75 | 6.64 | 7.70 | 9.00 | 8.77 | 8.61 | 8.09 | 7.02 | 6.91 | 3.23 | 1.91 | 1.60 | —— | —— | —— | —— |
|  | | FED304 | 1 | TR | 2 | BQL | 1.32 | 4.78 | 8.67 | 12.9 | 14.8 | 15.0 | 14.8 | 14.1 | 13.4 | 13.5 | 12.0 | 10.9 | 10.3 | 4.88 | 2.63 | 1.82 | 1.17 | 0.966 | 0.700 | 0.693 |
|  | | FED305 | 2 | RT | 2 | BQL | 1.13 | 4.00 | 8.07 | 12.1 | 13.3 | 14.0 | 14.1 | 13.4 | 12.3 | 11.7 | 10.4 | 9.51 | 8.25 | 3.66 | 1.69 | 1.45 | 1.02 | 0.923 | 0.696 | 0.547 |
|  | | FED306 | 1 | TR | 2 | BQL | 0.598 | 1.81 | 3.77 | 8.91 | 11.5 | 13.1 | 13.5 | 14.1 | 13.1 | 12.9 | 10.7 | 9.33 | 8.62 | 3.59 | 2.12 | 1.76 | 1.19 | 1.04 | 0.716 | 0.656 |
|  | | FED307 | 1 | TR | 2 | BQL | BQL | 1.83 | 3.81 | 7.52 | 10.8 | 11.4 | 12.3 | 11.3 | 11.1 | 10.8 | 9.92 | 8.65 | 7.80 | 3.26 | 1.87 | 1.45 | 1.45 | 1.36 | 0.979 | 0.828 |
|  | | FED309 | 1 | TR | 2 | BQL | 1.27 | 3.74 | 5.73 | 8.27 | 10.5 | 10.8 | 11.3 | 11.3 | 10.4 | 10.2 | 9.01 | 8.49 | 7.59 | 3.55 | 2.13 | 1.70 | 1.40 | 0.989 | 0.648 | 0.513 |
|  | | FED310 | 2 | RT | 2 | BQL | 14.9 | 14.3 | 16.2 | 18.0 | 18.4 | 17.7 | 16.6 | 14.5 | 13.8 | 12.2 | 10.8 | 9.33 | 7.64 | 2.99 | 1.70 | 1.19 | 0.908 | 0.790 | 0.566 | BQL |
|  | | FED311 | 1 | TR | 2 | BQL | 0.919 | 2.52 | 4.44 | 9.19 | 15.6 | 15.1 | 18.0 | 17.9 | 17.3 | 15.8 | 14.3 | 13.2 | 11.4 | 5.91 | 3.41 | 2.76 | 1.94 | 1.42 | 1.10 | 1.00 |
|  | | FED312 | 2 | RT | 2 | 0.570 | 0.712 | 2.00 | 4.56 | 8.73 | 11.3 | 13.2 | 12.7 | 13.7 | 13.1 | 12.9 | 11.2 | 10.5 | 9.66 | 4.73 | 3.13 | 2.42 | 2.26 | 1.85 | 1.53 | 1.27 |
|  | |  | | | N | 38 | 38 | 38 | 38 | 38 | 38 | 38 | 38 | 38 | 38 | 38 | 38 | 38 | 38 | 38 | 38 | 38 | 37 | 37 | 37 | 37 |
|  | |  | | | Mean | 0.0312 | 1.15 | 3.11 | 6.10 | 9.74 | 12.0 | 13.1 | 13.5 | 13.4 | 12.8 | 12.2 | 11.0 | 9.90 | 9.03 | 4.05 | 2.23 | 1.79 | 1.39 | 1.10 | 0.750 | 0.588 |
|  | |  | | | SD | 0.134 | 2.42 | 2.63 | 4.32 | 5.34 | 5.23 | 5.00 | 4.56 | 4.01 | 3.73 | 3.44 | 2.95 | 2.69 | 2.45 | 1.06 | 0.559 | 0.473 | 0.452 | 0.300 | 0.329 | 0.391 |
|  | |  | | | CV% | 430.3 | 211.1 | 84.4 | 70.9 | 54.8 | 43.7 | 38.1 | 33.8 | 30.0 | 29.2 | 28.1 | 26.9 | 27.2 | 27.1 | 26.2 | 25.1 | 26.4 | 32.5 | 27.2 | 43.8 | 66.4 |
|  | |  | | | Min | 0.00 | 0.00 | 0.00 | 1.18 | 1.56 | 2.64 | 3.15 | 3.64 | 4.14 | 4.22 | 4.17 | 4.34 | 4.06 | 3.81 | 2.58 | 1.38 | 1.05 | 0.657 | 0.585 | 0.00 | 0.00 |
|  | |  | | | Max | 0.615 | 14.9 | 14.3 | 17.2 | 23.9 | 26.4 | 28.0 | 27.9 | 26.1 | 25.3 | 24.3 | 21.4 | 20.3 | 18.2 | 8.21 | 3.52 | 2.85 | 2.72 | 1.85 | 1.53 | 1.47 |

Table S6.Plasma concentration of lisinopril (ng/ml) after oral administration of reference Lisinopril/amlodipine tablets (lisinopril 10mg / amlodipine 5 mg) to 37 subjects under fed condition

|  | | | | | | Group | | | | | | | | | | | | | | | | | | | | |
| --- | --- | --- | --- | --- | --- | --- | --- | --- | --- | --- | --- | --- | --- | --- | --- | --- | --- | --- | --- | --- | --- | --- | --- | --- | --- | --- |
|  |  |  |  |  |  | FED | | | | | | | | | | | | | | | | | | | | |
|  |  |  |  |  |  | Nominal_Time | | | | | | | | | | | | | | | | | | | | |
|  |  |  |  |  |  | (h) | | | | | | | | | | | | | | | | | | | | |
|  |  |  |  |  |  | 0 | 1 | 2 | 3 | 4 | 5 | 6 | 7 | 8 | 9 | 10 | 11 | 12 | 13 | 24 | 36 | 48 | 72 | 96 | 144 | 168 |
| Analyte | Formulation | Subject | Period | Sequence | Stage | Concentration | | | | | | | | | | | | | | | | | | | | |
|  |  |  |  |  |  | (ng/ml) | | | | | | | | | | | | | | | | | | | | |
| LSN | R | FED001 | 1 | RT | 1 | BQL | BQL | 0.974 | 2.13 | 4.64 | 7.46 | 9.64 | 10.8 | 10.0 | 9.60 | 9.19 | 8.03 | 7.04 | 6.13 | 2.57 | 1.47 | 1.26 | 0.862 | 0.640 | BQL | BQL |
|  | | FED002 | 1 | RT | 1 | BQL | BQL | 1.05 | 2.43 | 5.23 | 8.30 | 9.30 | 10.0 | 10.1 | 9.73 | 9.87 | 9.09 | 7.70 | 7.32 | 3.35 | 1.94 | 1.80 | 1.27 | 0.818 | BQL | BQL |
|  | | FED003 | 2 | TR | 1 | BQL | 0.899 | 3.60 | 11.1 | 14.2 | 16.7 | 18.5 | 20.4 | 19.0 | 15.2 | 17.9 | 15.8 | 14.3 | 12.8 | 4.92 | 2.19 | 1.37 | 0.899 | 0.756 | BQL | BQL |
|  | | FED004 | 2 | TR | 1 | BQL | BQL | BQL | 0.951 | 2.11 | 4.25 | 7.42 | 9.38 | 10.9 | 12.7 | 13.1 | 11.6 | 10.5 | 9.69 | 5.19 | 3.19 | 2.42 | 1.94 | 1.62 | 1.05 | 0.904 |
|  | | FED005 | 1 | RT | 1 | BQL | BQL | 2.22 | 4.36 | 11.0 | 12.6 | 13.5 | 13.0 | 12.3 | 12.3 | 11.3 | 9.71 | 8.65 | 7.78 | 3.34 | 1.84 | 1.48 | 1.42 | 1.10 | 0.866 | 0.689 |
|  | | FED006 | 2 | TR | 1 | BQL | 0.567 | 1.30 | 1.78 | 2.71 | 3.94 | 5.23 | 5.93 | 6.87 | 7.48 | 7.76 | 7.82 | 7.78 | 7.39 | 4.69 | 2.97 | 2.66 | 2.87 | 2.72 | 2.07 | 1.66 |
|  | | FED007 | 1 | RT | 1 | BQL | 0.719 | 2.62 | 10.4 | 15.9 | 18.9 | 20.0 | 21.2 | 19.7 | 19.2 | 17.4 | 15.9 | 13.3 | 11.9 | 4.29 | 2.17 | 1.60 | 1.11 | 0.811 | BQL | BQL |
|  | | FED008 | 2 | TR | 1 | BQL | 1.48 | 8.73 | 16.8 | 20.7 | 22.9 | 22.4 | 22.6 | 22.4 | 21.1 | 20.7 | 17.5 | 15.9 | 14.6 | 5.05 | 2.45 | 2.09 | 2.02 | 1.59 | 0.911 | 0.780 |
|  | | FED009 | 1 | RT | 1 | BQL | BQL | BQL | 0.890 | 1.62 | 2.12 | 3.45 | 4.83 | 5.67 | 6.73 | 7.39 | 7.38 | 7.40 | 7.16 | 3.46 | 1.98 | 1.57 | 1.18 | 0.868 | 0.569 | 0.534 |
|  | | FED010 | 1 | RT | 1 | BQL | BQL | BQL | 1.35 | 1.50 | 2.84 | 4.65 | 6.05 | 6.73 | 7.55 | 7.63 | 7.58 | 6.90 | 6.49 | 2.58 | 1.27 | 0.976 | 0.749 | 0.564 | BQL | BQL |
|  | | FED011 | 2 | TR | 1 | BQL | 3.22 | 8.41 | 18.6 | 23.8 | 23.9 | 23.2 | 22.5 | 21.2 | 19.2 | 19.0 | 16.0 | 14.4 | 12.1 | 4.93 | 3.14 | 2.17 | 1.38 | 1.22 | 0.775 | 0.708 |
|  | | FED012 | 2 | TR | 1 | BQL | 1.13 | 5.01 | 15.3 | 20.9 | 24.9 | 26.2 | 26.5 | 24.8 | 23.0 | 21.5* | 19.6 | 15.7 | 13.0 | 4.22 | 2.00 | 1.56 | 1.02 | 0.965 | 0.691 | 0.504 |
|  | | FED026 | 1 | RT | 1 | BQL | 0.941 | 2.81 | 2.78 | 5.24 | 6.27 | 7.59 | 8.11 | 8.17 | 7.93 | 7.32 | 6.64 | 6.19 | 5.38 | 3.38 | 2.00 | 1.77 | 1.29 | 0.951 | 0.777 | 0.629 |
|  | | FED027 | 2 | TR | 1 | BQL | 3.14 | 12.0 | 22.4 | 29.2 | 29.7 | 29.5 | 29.8 | 27.4 | 27.1 | 24.1 | 21.9 | 19.8 | 17.0 | 6.18 | 3.20 | 2.14 | 1.62 | 1.12 | 0.834 | 0.750 |
|  | | FED028 | 2 | TR | 1 | BQL | 0.842 | 3.23 | 4.39 | 7.68 | 10.4 | 11.1 | 11.8 | 12.2 | 12.1 | 11.4 | 9.94 | 9.14 | 8.51 | 3.84 | 2.06 | 1.80 | 1.64 | 1.59 | 0.868 | 0.711 |
|  | | FED029 | 1 | RT | 1 | BQL | BQL | 0.551 | 0.963 | 1.41 | 3.00 | 4.80 | 5.47 | 7.09 | 7.68 | 7.11 | 6.84 | 6.38 | 5.89 | 2.51 | 1.36 | 1.12 | 0.843 | 0.721 | 0.514 | BQL |
|  | | FED030 | 2 | TR | 1 | BQL | 1.25 | 3.71 | 9.06 | 14.0 | 16.9 | 17.9 | 18.3 | 18.3 | 17.0 | 16.1 | 15.5 | 13.8 | 12.7 | 4.87 | 2.16 | 1.54 | 1.10 | 0.753 | BQL | BQL |
|  | | FED032 | 1 | RT | 1 | BQL | 0.826 | 1.11 | 1.39 | 3.07 | 4.97 | 9.39 | 11.2 | 12.0 | 13.1 | 13.2 | 12.3 | 12.0 | 11.9 | 5.15 | 2.64 | 2.28 | 1.63 | 1.27 | 0.885 | 0.787 |
|  | | FED033 | 1 | RT | 1 | BQL | 0.793 | 1.93 | 2.48 | 4.77 | 6.31 | 6.90 | 7.13 | 7.60 | 7.64 | 8.05 | 7.48 | 7.24 | 6.52 | 4.06 | 2.77 | 2.16 | 1.60 | 1.33 | 0.919 | 0.832 |
|  | | FED034 | 2 | TR | 1 | BQL | 1.55 | 3.60 | 5.52 | 9.40 | 12.0 | 13.5 | 14.0 | 14.0 | 13.5 | 13.2 | 12.0 | 10.7 | 9.74 | 5.23 | 3.07 | 2.48 | 1.87 | 1.53 | 1.07 | 0.987 |
|  | | FED035 | 1 | RT | 1 | BQL | 0.556 | 2.32 | 4.13 | 8.38 | 13.7 | 19.6 | 22.4 | 23.0 | 23.1 | 23.2 | 21.7 | 20.8 | 19.0 | 8.44 | 3.64 | 2.55 | 1.60 | 1.43 | 1.00 | 0.890 |
|  | | FED036 | 2 | TR | 1 | 0.517 | 0.744 | 1.96 | 2.99 | 5.36 | 8.62 | 10.4 | 11.9 | 11.8 | 12.3 | 12.5 | 12.5 | 10.8 | 10.2 | 5.60 | 3.44 | 3.17 | 2.02 | 1.75 | 1.32 | 1.20 |
|  | | FED037 | 2 | TR | 1 | BQL | 1.26 | 6.07 | 14.3 | 21.9 | 27.3 | 29.4 | 28.3 | 26.6 | 24.5 | 23.5 | 20.8 | 18.4 | 16.9 | 6.29 | 2.79 | 2.08 | 1.33 | 1.06 | 0.715 | 0.696 |
|  | | FED038 | 2 | TR | 1 | BQL | 1.43 | 4.97 | 13.7 | 20.4 | 22.8 | 23.4 | 22.3 | 21.4 | 18.8 | 18.2 | 15.7 | 14.6 | 12.7 | 5.54 | 2.61 | 2.17 | 1.58 | 1.21 | 0.954 | 0.773 |
|  | | FED039 | 1 | RT | 1 | BQL | BQL | 1.26 | 2.22 | 4.41 | 7.52 | 9.63 | 11.2 | 12.5 | 12.3 | 11.5 | 10.7 | 9.83 | 9.22 | 4.35 | 1.86 | 1.43 | 0.978 | 0.738 | BQL | 0.528 |
|  | | FED040 | 1 | RT | 1 | BQL | 0.842 | 1.90 | 2.21 | 3.00 | 3.80 | 4.50 | 5.38 | 5.86 | 6.24 | 6.48 | 6.33 | 6.03 | 5.65 | 3.44 | 2.02 | 1.94 | 2.10 | 2.34 | 1.83 | 1.38 |
|  | | FED301 | 1 | RT | 2 | BQL | 0.747 | 1.73 | 4.04 | 9.13 | 13.9 | 16.2 | 17.3 | 17.4 | 17.5 | 16.4 | 14.7 | 13.2 | 12.1 | 4.26 | 1.95 | 1.66 | 1.12 | 1.00 | 0.661 | 0.522 |
|  | | FED302 | 1 | RT | 2 | BQL | BQL | 0.762 | 1.15 | 1.37 | 2.69 | 3.02 | 3.34 | 3.75 | 4.20 | 4.09 | 3.97 | 3.57 | 3.73 | 2.31 | 1.67 | 1.54 | 1.53 | 1.51 | 0.869 | 0.761 |
|  | | FED304 | 2 | TR | 2 | BQL | 1.81 | 5.03 | 9.00 | 13.6 | 15.4 | 15.8 | 15.0 | 14.9 | 13.4 | 13.1 | 11.8 | 10.7 | 10.2 | 4.55 | 2.55 | 1.81 | 1.28 | 1.03 | 0.760 | 0.659 |
|  | | FED305 | 1 | RT | 2 | BQL | BQL | 1.88 | 2.81 | 6.21 | 9.94 | 11.4 | 9.32 | 13.4 | 12.7 | 12.4 | 10.5 | 9.95 | 9.02 | 3.69 | 1.72 | 1.32 | 0.842 | 0.751 | 0.582 | 0.502 |
|  | | FED306 | 2 | TR | 2 | BQL | 0.710 | 1.75 | 2.42 | 5.91 | 8.56 | 10.5 | 11.4 | 11.1 | 11.1 | 10.5 | 9.38 | 8.51 | 8.21 | 3.86 | 2.30 | 2.15 | 1.77 | 1.20 | 0.969 | 0.849 |
|  | | FED307 | 2 | TR | 2 | BQL | 0.799 | 1.71 | 3.82 | 8.83 | 12.9 | 13.9 | 14.7 | 14.2 | 13.9 | 13.2 | 12.3 | 11.2 | 9.63 | 4.16 | 2.36 | 2.02 | 1.91 | 1.51 | 0.992 | 0.785 |
|  | | FED308 | 1 | RT | 2 | BQL | BQL | 1.76 | 2.63 | 8.04 | 15.0 | 18.3 | 20.5 | 21.6 | 20.5 | 19.2 | 17.1 | 13.9 | 12.4 | 4.43 | 2.10 | 1.54 | 1.09 | 0.824 | 0.736 | 0.620 |
|  | | FED309 | 2 | TR | 2 | BQL | 0.831 | 2.13 | 3.45 | 5.69 | 7.38 | 7.82 | 8.84 | 9.01 | 8.15 | 8.64 | 7.85 | 7.18 | 6.35 | 3.04 | 1.91 | 1.58 | 1.31 | 1.07 | 0.576 | 0.572 |
|  | | FED310 | 1 | RT | 2 | BQL | BQL | 0.838 | 2.85 | 8.35 | 12.5 | 13.5 | 12.7 | 13.3 | 12.4 | 11.2 | 9.74 | 8.33 | 7.35 | 3.04 | 1.44 | 1.06 | 0.736 | 0.620 | BQL | BQL |
|  | | FED311 | 2 | TR | 2 | 0.532 | 2.32 | 5.33 | 10.6 | 11.7 | 15.2 | 14.9 | 14.3 | 14.1 | 13.7 | 13.6 | 12.9 | 11.3 | 10.4 | 5.61 | 3.61 | 3.23 | 2.70 | 2.09 | 1.56 | 1.38 |
|  | | FED312 | 1 | RT | 2 | BQL | BQL | 1.01 | 1.75 | 2.57 | 4.10 | 4.56 | 5.73 | 6.40 | 6.39 | 6.61 | 6.48 | 6.23 | 5.77 | 3.56 | 2.18 | 2.03 | 1.76 | 1.64 | 1.10 | 1.04 |
|  | |  | | | N | 37 | 37 | 37 | 37 | 37 | 37 | 37 | 37 | 37 | 37 | 36 | 37 | 37 | 37 | 37 | 37 | 37 | 37 | 37 | 37 | 37 |
|  | |  | | | Mean | 0.0284 | 0.795 | 2.85 | 5.92 | 9.30 | 11.9 | 13.3 | 13.9 | 14.0 | 13.5 | 12.9 | 12.0 | 10.8 | 9.81 | 4.32 | 2.32 | 1.88 | 1.46 | 1.21 | 0.741 | 0.639 |
|  | |  | | | SD | 0.120 | 0.835 | 2.62 | 5.72 | 7.24 | 7.54 | 7.33 | 7.10 | 6.40 | 5.77 | 5.25 | 4.69 | 4.09 | 3.57 | 1.24 | 0.631 | 0.528 | 0.503 | 0.490 | 0.505 | 0.422 |
|  | |  | | | CV% | 424.1 | 105.1 | 92.2 | 96.6 | 77.9 | 63.5 | 55.3 | 51.2 | 45.9 | 42.6 | 40.6 | 39.2 | 37.9 | 36.4 | 28.8 | 27.1 | 28.1 | 34.5 | 40.5 | 68.2 | 66.1 |
|  | |  | | | Min | 0.00 | 0.00 | 0.00 | 0.890 | 1.37 | 2.12 | 3.02 | 3.34 | 3.75 | 4.20 | 4.09 | 3.97 | 3.57 | 3.73 | 2.31 | 1.27 | 0.976 | 0.736 | 0.564 | 0.00 | 0.00 |
|  | |  | | | Max | 0.532 | 3.22 | 12.0 | 22.4 | 29.2 | 29.7 | 29.5 | 29.8 | 27.4 | 27.1 | 24.1 | 21.9 | 20.8 | 19.0 | 8.44 | 3.64 | 3.23 | 2.87 | 2.72 | 2.07 | 1.66 |

Table S7.Plasma concentration of amlodipine (ng/ml) after oral administration of test Lisinopril/amlodipine tablets (lisinopril 10mg / amlodipine 5 mg) to 38 subjects under fed condition

|  | | | | | | Group | | | | | | | | | | | | | | | | | | | | |
| --- | --- | --- | --- | --- | --- | --- | --- | --- | --- | --- | --- | --- | --- | --- | --- | --- | --- | --- | --- | --- | --- | --- | --- | --- | --- | --- |
|  |  |  |  |  |  | FED | | | | | | | | | | | | | | | | | | | | |
|  |  |  |  |  |  | Nominal_Time | | | | | | | | | | | | | | | | | | | | |
|  |  |  |  |  |  | (h) | | | | | | | | | | | | | | | | | | | | |
|  |  |  |  |  |  | 0 | 1 | 2 | 3 | 4 | 5 | 6 | 7 | 8 | 9 | 10 | 11 | 12 | 13 | 24 | 36 | 48 | 72 | 96 | 144 | 168 |
| Analyte | Formulation | Subject | Period | Sequence | Stage | Concentration | | | | | | | | | | | | | | | | | | | | |
|  |  |  |  |  |  | (ng/ml) | | | | | | | | | | | | | | | | | | | | |
| Amlodipine | T | FED001 | 2 | RT | 1 | BQL | BQL | 0.0815 | 0.297 | 0.763 | 1.48 | 1.57 | 1.78 | 2.60 | 2.19 | 2.57 | 2.95 | 2.79 | 2.47 | 1.73 | 1.36 | 1.01 | 0.582 | 0.374 | 0.132 | 0.108 |
|  | | FED002 | 2 | RT | 1 | 0.0785 | 0.182 | 0.747 | 1.41 | 1.51 | 1.72 | 1.88 | 1.99 | 1.81 | 1.73 | 1.71 | 1.74 | 1.77 | 1.81 | 1.41 | 1.28 | 1.16 | 0.976 | 0.692 | 0.416 | 0.322 |
|  | | FED003 | 1 | TR | 1 | BQL | 0.0519 | 0.186 | 0.736 | 0.994 | 1.59 | 1.92 | 2.04 | 2.05 | 2.12 | 1.90 | 2.01 | 1.83 | 1.65 | 1.45 | 1.26 | 1.05 | 0.692 | 0.557 | 0.268 | 0.194 |
|  | | FED004 | 1 | TR | 1 | BQL | BQL | BQL | 0.347 | 0.957 | 1.74 | 1.94 | 2.36 | 2.35 | 2.50 | 2.23 | 2.06 | 2.18 | 1.92 | 1.61 | 1.21 | 0.882 | 0.590 | 0.334 | 0.119 | 0.0722 |
|  | | FED005 | 2 | RT | 1 | BQL | 0.244 | 1.87 | 2.31 | 1.84 | 2.38 | 2.59 | 2.19 | 2.18 | 2.22 | 2.04 | 2.01 | 2.03 | 1.98 | 1.38 | 1.29 | 0.863 | 0.635 | 0.446 | 0.204 | 0.162 |
|  | | FED006 | 1 | TR | 1 | BQL | 0.270 | 0.620 | 0.991 | 0.863 | 1.32 | 1.31 | 1.15 | 1.13 | 1.04 | 0.876 | 0.900 | 0.940 | 0.858 | 0.813 | 0.580 | 0.502 | 0.345 | 0.256 | 0.0991 | 0.0592 |
|  | | FED007 | 2 | RT | 1 | BQL | BQL | 0.0745 | 0.521 | 1.04 | 1.57 | 2.35 | 2.35 | 2.90 | 2.16 | 2.09 | 2.36 | 2.29 | 2.13 | 1.72 | 1.45 | 1.01 | 0.640 | 0.396 | 0.138 | 0.105 |
|  | | FED008 | 1 | TR | 1 | BQL | 0.262 | 0.498 | 1.41 | 0.863 | 1.56 | 1.60 | 1.48 | 1.44 | 1.83 | 1.76 | 1.69 | 1.73 | 1.55 | 1.47 | 1.21 | 1.00 | 0.748 | 0.499 | 0.208 | 0.160 |
|  | | FED009 | 2 | RT | 1 | BQL | 0.373 | 1.31 | 1.52 | 1.41 | 1.68 | 1.60 | 1.79 | 1.74 | 1.74 | 1.81 | 1.86 | 1.56 | 1.79 | 1.37 | 1.19 | 1.05 | 0.734 | 0.514 | 0.218 | 0.181 |
|  | | FED010 | 2 | RT | 1 | BQL | BQL | 0.0567 | 0.196 | 0.238 | 0.830 | 1.00 | 1.84 | 1.82 | 1.99 | 1.94 | 2.09 | 1.97 | 1.90 | 1.61 | 1.49 | 1.14 | 0.768 | 0.640 | 0.265 | 0.209 |
|  | | FED011 | 1 | TR | 1 | BQL | 1.29 | 1.99 | 2.51 | 2.11 | 2.01 | 1.91 | 2.05 | 1.98 | 1.83 | 1.86 | 1.75 | 1.65 | 1.66 | 1.33 | 0.870 | 0.805 | 0.483 | 0.292 | 0.103 | 0.0781 |
|  | | FED012 | 1 | TR | 1 | BQL | BQL | 0.319 | 0.615 | 1.01 | 1.73 | 1.61 | 1.66 | 1.51 | 2.16 | 2.07 | 2.25 | 2.33 | 2.14 | 1.83 | 1.37 | 1.09 | 0.717 | 0.482 | 0.201 | 0.130 |
|  | | FED026 | 2 | RT | 1 | BQL | 0.110 | 0.643 | 0.550 | 0.577 | 1.31 | 1.26 | 1.31 | 1.70 | 1.67 | 1.35 | 1.45 | 1.37 | 1.36 | 1.25 | 0.939 | 0.811 | 0.468 | 0.251 | 0.0994 | 0.0775 |
|  | | FED027 | 1 | TR | 1 | BQL | 0.720 | 1.30 | 1.56 | 1.42 | 1.49 | 1.66 | 1.54 | 1.62 | 1.57 | 1.53 | 1.41 | 1.37 | 1.40 | 1.23 | 0.951 | 0.816 | 0.589 | 0.391 | 0.196 | 0.119 |
|  | | FED028 | 1 | TR | 1 | BQL | 0.463 | 1.85 | 1.61 | 2.14 | 2.03 | 2.39 | 2.26 | 2.32 | 2.26 | 1.97 | 1.95 | 2.01 | 1.92 | 1.85 | 1.56 | 1.48 | 0.992 | 0.900 | 0.462 | 0.454 |
|  | | FED029 | 2 | RT | 1 | BQL | BQL | 0.196 | 0.248 | 0.390 | 0.702 | 1.07 | 1.35 | 1.43 | 1.10 | 1.20 | 1.49 | 1.29 | 1.49 | 1.23 | 1.01 | 0.809 | 0.631 | 0.468 | 0.253 | 0.217 |
|  | | FED030 | 1 | TR | 1 | BQL | 0.836 | 2.00 | 1.65 | 1.83 | 1.68 | 1.66 | 1.48 | 1.54 | 1.48 | 1.48 | 1.35 | 1.51 | 1.53 | 1.15 | 0.939 | 0.751 | 0.506 | 0.321 | 0.143 | 0.107 |
|  | | FED031 | 1 | TR | 1 | BQL | 0.0775 | 0.184 | 0.391 | 0.721 | 1.13 | 1.23 | 1.52 | 1.53 | 1.56 | 1.55 | 1.69 | 1.62 | 1.74 | 1.42 | 0.998 | 0.822 | 0.541 | 0.326 | 0.136 | 0.0947 |
|  | | FED032 | 2 | RT | 1 | BQL | 0.0677 | 0.422 | 0.466 | 0.938 | 1.24 | 1.64 | 1.39 | 1.56 | 1.39 | 1.39 | 1.41 | 1.63 | 1.53 | 1.45 | 1.21 | 0.954 | 0.644 | 0.421 | 0.213 | 0.141 |
|  | | FED033 | 2 | RT | 1 | BQL | 0.0526 | 0.105 | 0.217 | 0.569 | 1.08 | 1.52 | 1.68 | 1.82 | 1.79 | 1.89 | 1.93 | 2.01 | 1.95 | 1.64 | 1.42 | 1.15 | 0.852 | 0.638 | 0.364 | 0.298 |
|  | | FED034 | 1 | TR | 1 | BQL | 0.462 | 0.710 | 0.738 | 0.934 | 1.03 | 1.06 | 1.05 | 1.20 | 1.18 | 1.09 | 1.11 | 1.18 | 1.17 | 0.928 | 0.750 | 0.551 | 0.369 | 0.252 | 0.102 | 0.0897 |
|  | | FED035 | 2 | RT | 1 | BQL | 0.312 | 0.851 | 1.10 | 1.23 | 1.72 | 1.33 | 1.45 | 1.61 | 1.36 | 1.38 | 1.23 | 1.24 | 1.06 | 0.897 | 0.647 | 0.487 | 0.373 | 0.243 | 0.107 | 0.0773 |
|  | | FED036 | 1 | TR | 1 | BQL | 0.249 | 0.716 | 0.845 | 1.22 | 1.62 | 1.51 | 1.43 | 1.59 | 1.54 | 1.56 | 1.52 | 1.46 | 1.38 | 1.37 | 1.10 | 0.927 | 0.725 | 0.494 | 0.235 | 0.202 |
|  | | FED037 | 1 | TR | 1 | BQL | 1.00 | 1.84 | 1.84 | 2.22 | 2.35 | 2.39 | 2.31 | 2.52 | 2.36 | 2.10 | 2.06 | 1.93 | 1.92 | 1.86 | 1.54 | 1.24 | 1.03 | 0.699 | 0.380 | 0.293 |
|  | | FED038 | 1 | TR | 1 | BQL | 0.0674 | 0.0731 | 0.240 | 0.450 | 0.904 | 1.27 | 1.49 | 1.79 | 1.65 | 1.77 | 1.81 | 1.74 | 1.82 | 1.35 | 0.907 | 0.785 | 0.493 | 0.274 | 0.108 | 0.0543 |
|  | | FED039 | 2 | RT | 1 | BQL | BQL | 0.244 | 0.790 | 1.25 | 1.79 | 1.69 | 2.32 | 2.15 | 2.05 | 1.90 | 1.99 | 1.99 | 1.87 | 1.73 | 1.49 | 1.19 | 0.912 | 0.641 | 0.310 | 0.232 |
|  | | FED040 | 2 | RT | 1 | BQL | 0.840 | 1.10 | 1.14 | 1.40 | 1.15 | 1.41 | 1.49 | 1.58 | 1.36 | 1.22 | 1.26 | 1.25 | 1.21 | 1.02 | 0.814 | 0.599 | 0.415 | 0.297 | 0.141 | 0.113 |
|  | | FED301 | 2 | RT | 2 | BQL | 0.0822 | 0.462 | 0.751 | 1.08 | 1.40 | 1.73 | 1.69 | 1.62 | 1.54 | 1.42 | 1.52 | 1.51 | 1.38 | 1.08 | 0.950 | 0.611 | 0.430 | 0.313 | 0.103 | 0.0752 |
|  | | FED302 | 2 | RT | 2 | BQL | BQL | BQL | 0.196 | 0.459 | 0.873 | 0.909 | 1.35 | 1.61 | 1.48 | 1.50 | 1.69 | 1.62 | 1.80 | 1.25 | 1.01 | 0.722 | 0.519 | 0.288 | 0.0948 | 0.0744 |
|  | | FED303 | 1 | TR | 2 | BQL | 0.224 | 1.28 | 0.948 | 0.820 | 1.55 | 1.33 | 1.25 | 1.48 | 1.24 | 1.17 | 1.06 | 1.10 | 1.10 | 1.00 | 0.804 | 0.756 | —— | —— | —— | —— |
|  | | FED304 | 1 | TR | 2 | BQL | 0.721 | 1.54 | 1.42 | 1.37 | 1.92 | 1.57 | 1.60 | 1.51 | 1.36 | 1.33 | 1.29 | 1.35 | 1.33 | 1.21 | 0.945 | 0.882 | 0.624 | 0.448 | 0.251 | 0.200 |
|  | | FED305 | 2 | RT | 2 | BQL | 0.263 | 0.757 | 0.732 | 0.961 | 1.10 | 1.38 | 1.42 | 1.54 | 1.39 | 1.32 | 1.54 | 1.48 | 1.37 | 1.31 | 1.04 | 0.874 | 0.750 | 0.507 | 0.233 | 0.161 |
|  | | FED306 | 1 | TR | 2 | BQL | 0.0601 | 0.444 | 0.780 | 0.763 | 1.41 | 1.51 | 1.51 | 1.68 | 1.54 | 1.50 | 1.41 | 1.65 | 1.57 | 1.38 | 1.12 | 0.929 | 0.694 | 0.463 | 0.224 | 0.173 |
|  | | FED307 | 1 | TR | 2 | BQL | 0.194 | 0.430 | 0.719 | 1.19 | 1.74 | 1.69 | 1.27 | 1.36 | 1.92 | 1.64 | 1.63 | 1.82 | 2.04 | 1.61 | 1.49 | 1.24 | 0.920 | 0.671 | 0.337 | 0.280 |
|  | | FED309 | 1 | TR | 2 | BQL | 0.977 | 1.51 | 1.39 | 1.32 | 1.61 | 1.49 | 1.32 | 1.22 | 1.13 | 1.09 | 1.12 | 1.23 | 1.10 | 0.988 | 0.786 | 0.665 | 0.482 | 0.319 | 0.122 | 0.0992 |
|  | | FED310 | 2 | RT | 2 | BQL | 0.105 | 0.154 | 0.228 | 0.601 | 1.55 | 1.63 | 1.53 | 2.03 | 1.45 | 1.53 | 1.81 | 1.72 | 1.59 | 1.27 | 1.10 | 0.852 | 0.492 | 0.310 | 0.124 | 0.0789 |
|  | | FED311 | 1 | TR | 2 | BQL | BQL | 0.109 | 0.361 | 0.931 | 1.75 | 1.49 | 2.23 | 2.15 | 1.96 | 1.91 | 1.91 | 1.93 | 1.80 | 1.48 | 1.12 | 0.912 | 0.525 | 0.313 | 0.123 | 0.0595 |
|  | | FED312 | 2 | RT | 2 | BQL | BQL | 0.0933 | 0.592 | 0.566 | 1.14 | 1.69 | 1.32 | 1.61 | 1.44 | 1.34 | 1.17 | 1.16 | 1.13 | 0.971 | 0.833 | 0.723 | 0.641 | 0.409 | 0.180 | 0.115 |
|  | |  | | | N | 38 | 38 | 38 | 38 | 38 | 38 | 38 | 38 | 38 | 38 | 38 | 38 | 38 | 38 | 38 | 38 | 38 | 37 | 37 | 37 | 37 |
|  | |  | | | Mean | 0.00207 | 0.278 | 0.704 | 0.904 | 1.08 | 1.50 | 1.60 | 1.66 | 1.77 | 1.69 | 1.63 | 1.67 | 1.66 | 1.62 | 1.36 | 1.11 | 0.897 | 0.636 | 0.436 | 0.200 | 0.153 |
|  | |  | | | SD | 0.0127 | 0.341 | 0.644 | 0.599 | 0.491 | 0.392 | 0.381 | 0.374 | 0.399 | 0.380 | 0.367 | 0.414 | 0.388 | 0.358 | 0.279 | 0.262 | 0.221 | 0.180 | 0.158 | 0.0978 | 0.0893 |
|  | |  | | | CV% | 616.4 | 122.7 | 91.5 | 66.2 | 45.6 | 26.2 | 23.8 | 22.5 | 22.5 | 22.5 | 22.5 | 24.8 | 23.3 | 22.1 | 20.5 | 23.7 | 24.6 | 28.3 | 36.2 | 48.8 | 58.3 |
|  | |  | | | Min | 0.00 | 0.00 | 0.00 | 0.196 | 0.238 | 0.702 | 0.909 | 1.05 | 1.13 | 1.04 | 0.876 | 0.900 | 0.940 | 0.858 | 0.813 | 0.580 | 0.487 | 0.345 | 0.243 | 0.0948 | 0.0543 |
|  | |  | | | Max | 0.0785 | 1.29 | 2.00 | 2.51 | 2.22 | 2.38 | 2.59 | 2.36 | 2.90 | 2.50 | 2.57 | 2.95 | 2.79 | 2.47 | 1.86 | 1.56 | 1.48 | 1.03 | 0.900 | 0.462 | 0.454 |

Table S8.Plasma concentration of amlodipine (ng/ml) after oral administration of reference Lisinopril/amlodipine tablets (lisinopril 10mg / amlodipine 5 mg) to 37 subjects under fed condition

|  | | | | | | Group | | | | | | | | | | | | | | | | | | | | |
| --- | --- | --- | --- | --- | --- | --- | --- | --- | --- | --- | --- | --- | --- | --- | --- | --- | --- | --- | --- | --- | --- | --- | --- | --- | --- | --- |
|  |  |  |  |  |  | FED | | | | | | | | | | | | | | | | | | | | |
|  |  |  |  |  |  | Nominal_Time | | | | | | | | | | | | | | | | | | | | |
|  |  |  |  |  |  | (h) | | | | | | | | | | | | | | | | | | | | |
|  |  |  |  |  |  | 0 | 1 | 2 | 3 | 4 | 5 | 6 | 7 | 8 | 9 | 10 | 11 | 12 | 13 | 24 | 36 | 48 | 72 | 96 | 144 | 168 |
| Analyte | Formulation | Subject | Period | Sequence | Stage | Concentration | | | | | | | | | | | | | | | | | | | | |
|  |  |  |  |  |  | (ng/ml) | | | | | | | | | | | | | | | | | | | | |
| Amlodipine | R | FED001 | 1 | RT | 1 | BQL | 0.0617 | 0.243 | 0.789 | 1.02 | 1.73 | 1.75 | 2.17 | 2.51 | 2.49 | 2.49 | 2.49 | 2.42 | 2.39 | 2.15 | 1.67 | 1.27 | 0.736 | 0.500 | 0.176 | 0.112 |
|  | | FED002 | 1 | RT | 1 | BQL | 0.100 | 0.515 | 1.20 | 1.07 | 1.50 | 1.71 | 2.12 | 1.90 | 2.03 | 1.87 | 1.79 | 1.82 | 1.84 | 1.49 | 1.17 | 1.03 | 0.839 | 0.713 | 0.373 | 0.292 |
|  | | FED003 | 2 | TR | 1 | BQL | 0.639 | 0.932 | 1.56 | 1.26 | 2.06 | 1.86 | 2.12 | 2.54 | 1.90 | 2.36 | 2.25 | 2.08 | 2.20 | 1.86 | 1.43 | 1.22 | 1.02 | 0.680 | 0.358 | 0.271 |
|  | | FED004 | 2 | TR | 1 | BQL | BQL | BQL | 0.0820 | 0.340 | 0.994 | 1.54 | 1.85 | 2.02 | 2.32 | 2.35 | 2.01 | 2.29 | 2.09 | 1.64 | 1.23 | 0.993 | 0.582 | 0.395 | 0.154 | 0.111 |
|  | | FED005 | 1 | RT | 1 | BQL | 0.161 | 0.758 | 1.04 | 2.19 | 2.96 | 2.22 | 2.22 | 2.39 | 2.18 | 2.48 | 2.47 | 2.24 | 2.19 | 1.73 | 1.51 | 1.02 | 0.706 | 0.552 | 0.245 | 0.168 |
|  | | FED006 | 2 | TR | 1 | BQL | BQL | 0.341 | 0.310 | 0.421 | 1.10 | 1.16 | 0.932 | 0.988 | 0.899 | 0.908 | 0.864 | 0.914 | 0.879 | 0.730 | 0.532 | 0.425 | 0.305 | 0.220 | 0.0920 | 0.0589 |
|  | | FED007 | 1 | RT | 1 | BQL | 0.104 | 0.285 | 0.724 | 0.686 | 1.02 | 1.54 | 1.84 | 1.75 | 1.79 | 1.83 | 1.88 | 2.04 | 1.91 | 1.73 | 1.39 | 1.03 | 0.595 | 0.370 | 0.123 | 0.0757 |
|  | | FED008 | 2 | TR | 1 | BQL | 0.199 | 0.577 | 1.56 | 1.44 | 2.18 | 1.68 | 1.91 | 2.00 | 2.06 | 1.85 | 2.09 | 1.69 | 2.08 | 1.90 | 1.31 | 1.32 | 1.08 | 0.752 | 0.391 | 0.277 |
|  | | FED009 | 1 | RT | 1 | BQL | BQL | 0.104 | 0.326 | 0.830 | 1.19 | 1.48 | 1.52 | 1.60 | 1.87 | 1.58 | 1.51 | 1.55 | 1.57 | 1.36 | 1.04 | 0.893 | 0.639 | 0.444 | 0.180 | 0.126 |
|  | | FED010 | 1 | RT | 1 | BQL | BQL | 0.150 | 0.494 | 0.478 | 0.926 | 1.06 | 1.72 | 1.27 | 1.60 | 1.39 | 1.51 | 1.70 | 1.86 | 1.73 | 1.32 | 1.28 | 0.846 | 0.539 | 0.286 | 0.238 |
|  | | FED011 | 2 | TR | 1 | BQL | 0.686 | 0.944 | 1.30 | 1.71 | 1.93 | 1.52 | 1.86 | 1.98 | 1.86 | 1.73 | 1.60 | 1.56 | 1.65 | 1.33 | 1.10 | 0.744 | 0.459 | 0.330 | 0.128 | 0.0859 |
|  | | FED012 | 2 | TR | 1 | BQL | 0.112 | 0.350 | 0.721 | 0.821 | 1.60 | 1.39 | 1.64 | 1.88 | 1.80 | 2.30* | 2.53 | 2.35 | 2.27 | 1.89 | 1.62 | 1.21 | 0.812 | 0.631 | 0.286 | 0.173 |
|  | | FED026 | 1 | RT | 1 | BQL | 0.223 | 0.858 | 0.696 | 0.957 | 1.34 | 1.63 | 1.83 | 1.88 | 1.67 | 1.73 | 1.64 | 1.68 | 1.64 | 1.43 | 1.03 | 0.855 | 0.558 | 0.332 | 0.107 | 0.0929 |
|  | | FED027 | 2 | TR | 1 | BQL | 0.382 | 1.49 | 1.20 | 1.74 | 1.79 | 1.59 | 1.64 | 2.18 | 2.10 | 1.85 | 2.04 | 1.98 | 1.98 | 1.51 | 1.33 | 1.13 | 0.687 | 0.479 | 0.222 | 0.180 |
|  | | FED028 | 2 | TR | 1 | 0.0530 | 0.329 | 1.01 | 1.10 | 1.22 | 2.17 | 2.16 | 2.24 | 1.95 | 1.91 | 1.91 | 1.95 | 2.04 | 1.97 | 2.01 | 1.65 | 1.44 | 0.964 | 0.772 | 0.503 | 0.291 |
|  | | FED029 | 1 | RT | 1 | BQL | BQL | 0.188 | 0.251 | 0.388 | 0.915 | 1.30 | 1.34 | 1.90 | 1.57 | 1.28 | 1.47 | 1.60 | 1.50 | 1.42 | 1.18 | 1.13 | 0.703 | 0.566 | 0.327 | 0.249 |
|  | | FED030 | 2 | TR | 1 | BQL | 0.426 | 0.858 | 0.845 | 1.19 | 1.61 | 1.62 | 1.54 | 1.51 | 1.48 | 1.36 | 1.40 | 1.36 | 1.35 | 1.14 | 1.09 | 0.760 | 0.572 | 0.396 | 0.183 | 0.131 |
|  | | FED032 | 1 | RT | 1 | BQL | BQL | 0.0967 | 0.106 | 0.253 | 0.533 | 1.04 | 1.09 | 1.53 | 0.973 | 1.04 | 1.12 | 1.46 | 1.79 | 1.65 | 1.20 | 1.01 | 0.748 | 0.502 | 0.282 | 0.193 |
|  | | FED033 | 1 | RT | 1 | BQL | 0.238 | 0.953 | 1.23 | 1.90 | 2.07 | 1.97 | 1.85 | 2.01 | 1.84 | 1.84 | 1.87 | 2.00 | 2.02 | 1.69 | 1.37 | 1.14 | 0.786 | 0.602 | 0.251 | 0.197 |
|  | | FED034 | 2 | TR | 1 | BQL | 0.133 | 0.849 | 0.871 | 0.932 | 1.35 | 1.51 | 1.62 | 1.77 | 1.75 | 1.73 | 1.79 | 1.66 | 1.69 | 1.45 | 1.15 | 0.851 | 0.601 | 0.396 | 0.159 | 0.107 |
|  | | FED035 | 1 | RT | 1 | BQL | 0.0977 | 0.162 | 0.182 | 0.249 | 0.586 | 0.781 | 1.04 | 1.30 | 1.49 | 1.41 | 1.52 | 1.44 | 1.47 | 1.16 | 0.769 | 0.599 | 0.437 | 0.293 | 0.107 | 0.0744 |
|  | | FED036 | 2 | TR | 1 | BQL | 0.0879 | 0.235 | 0.691 | 0.990 | 1.55 | 1.60 | 1.57 | 1.54 | 1.48 | 1.58 | 1.67 | 1.70 | 1.54 | 1.39 | 1.18 | 1.04 | 0.695 | 0.594 | 0.314 | 0.236 |
|  | | FED037 | 2 | TR | 1 | 0.0701 | 0.262 | 0.664 | 0.638 | 0.930 | 1.95 | 2.71 | 1.85 | 2.60 | 2.40 | 2.42 | 2.60 | 2.52 | 2.38 | 2.34 | 1.98 | 1.67 | 1.23 | 0.947 | 0.564 | 0.422 |
|  | | FED038 | 2 | TR | 1 | BQL | 0.644 | 1.32 | 1.65 | 1.79 | 2.58 | 2.57 | 2.40 | 2.46 | 2.49 | 2.36 | 2.10 | 2.04 | 1.96 | 1.80 | 1.33 | 1.04 | 0.662 | 0.389 | 0.134 | 0.0930 |
|  | | FED039 | 1 | RT | 1 | BQL | 0.0968 | 0.357 | 0.656 | 1.06 | 1.73 | 2.06 | 1.95 | 1.97 | 1.96 | 1.96 | 1.91 | 2.01 | 2.04 | 1.90 | 1.45 | 1.28 | 0.919 | 0.652 | 0.331 | 0.229 |
|  | | FED040 | 1 | RT | 1 | BQL | 0.616 | 1.10 | 1.10 | 1.04 | 1.54 | 1.45 | 1.34 | 1.37 | 1.22 | 1.15 | 1.12 | 1.21 | 1.18 | 0.837 | 0.734 | 0.597 | 0.465 | 0.376 | 0.217 | 0.154 |
|  | | FED301 | 1 | RT | 2 | BQL | 0.457 | 0.721 | 0.436 | 0.873 | 1.56 | 1.70 | 1.55 | 1.52 | 1.64 | 1.51 | 1.61 | 1.68 | 1.60 | 1.29 | 1.16 | 0.849 | 0.542 | 0.365 | 0.121 | 0.0764 |
|  | | FED302 | 1 | RT | 2 | BQL | BQL | 0.0781 | 0.213 | 0.341 | 1.28 | 1.27 | 1.37 | 1.41 | 1.69 | 1.44 | 1.71 | 1.54 | 1.60 | 1.27 | 1.05 | 0.833 | 0.482 | 0.325 | 0.122 | 0.0698 |
|  | | FED304 | 2 | TR | 2 | BQL | 1.21 | 1.81 | 1.36 | 1.69 | 1.82 | 1.75 | 1.65 | 1.59 | 1.55 | 1.59 | 1.40 | 1.42 | 1.32 | 1.25 | 1.06 | 0.890 | 0.707 | 0.448 | 0.247 | 0.190 |
|  | | FED305 | 1 | RT | 2 | BQL | BQL | 0.159 | 0.394 | 0.645 | 0.985 | 1.13 | 1.02 | 1.32 | 1.54 | 1.47 | 1.30 | 1.42 | 1.37 | 1.24 | 1.04 | 0.901 | 0.630 | 0.443 | 0.230 | 0.174 |
|  | | FED306 | 2 | TR | 2 | BQL | 0.0888 | 0.311 | 0.576 | 0.818 | 1.15 | 1.41 | 1.55 | 1.70 | 1.65 | 1.69 | 1.52 | 1.55 | 2.00 | 1.51 | 1.07 | 0.939 | 0.732 | 0.528 | 0.274 | 0.181 |
|  | | FED307 | 2 | TR | 2 | BQL | 0.200 | 0.265 | 0.425 | 0.763 | 1.10 | 1.10 | 1.29 | 1.25 | 1.56 | 1.86 | 2.08 | 2.11 | 2.40 | 1.94 | 1.52 | 1.25 | 0.947 | 0.599 | 0.289 | 0.259 |
|  | | FED308 | 1 | RT | 2 | BQL | 0.0966 | 0.235 | 0.309 | 0.432 | 1.03 | 1.42 | 1.43 | 1.70 | 1.50 | 1.41 | 1.48 | 1.51 | 1.37 | 1.22 | 0.988 | 0.832 | 0.623 | 0.447 | 0.204 | 0.140 |
|  | | FED309 | 2 | TR | 2 | BQL | 0.240 | 0.391 | 0.681 | 0.704 | 1.35 | 1.43 | 1.38 | 1.28 | 1.21 | 1.21 | 1.26 | 1.31 | 1.13 | 0.950 | 0.738 | 0.607 | 0.444 | 0.364 | 0.159 | 0.129 |
|  | | FED310 | 1 | RT | 2 | BQL | BQL | BQL | 0.426 | 0.880 | 1.56 | 1.78 | 1.57 | 1.72 | 1.62 | 1.70 | 1.80 | 1.61 | 1.54 | 1.22 | 0.928 | 0.655 | 0.387 | 0.232 | 0.0906 | 0.0612 |
|  | | FED311 | 2 | TR | 2 | BQL | 0.279 | 0.906 | 1.41 | 0.793 | 1.29 | 1.70 | 1.56 | 1.94 | 1.81 | 1.61 | 1.99 | 1.78 | 1.97 | 1.44 | 1.27 | 0.897 | 0.594 | 0.370 | 0.132 | 0.0607 |
|  | | FED312 | 1 | RT | 2 | BQL | BQL | 0.292 | 0.628 | 0.911 | 1.33 | 1.32 | 1.44 | 1.45 | 1.42 | 1.35 | 1.31 | 1.21 | 1.22 | 1.07 | 0.755 | 0.616 | 0.499 | 0.331 | 0.118 | 0.0916 |
|  | |  | | | N | 37 | 37 | 37 | 37 | 37 | 37 | 37 | 37 | 37 | 37 | 36 | 37 | 37 | 37 | 37 | 37 | 37 | 37 | 37 | 37 | 37 |
|  | |  | | | Mean | 0.00333 | 0.221 | 0.554 | 0.762 | 0.966 | 1.50 | 1.59 | 1.65 | 1.78 | 1.74 | 1.70 | 1.75 | 1.74 | 1.76 | 1.50 | 1.20 | 0.980 | 0.682 | 0.483 | 0.229 | 0.164 |
|  | |  | | | SD | 0.0143 | 0.263 | 0.443 | 0.444 | 0.487 | 0.519 | 0.402 | 0.357 | 0.399 | 0.371 | 0.407 | 0.415 | 0.370 | 0.383 | 0.363 | 0.298 | 0.266 | 0.202 | 0.161 | 0.113 | 0.0845 |
|  | |  | | | CV% | 428.4 | 119.2 | 80.0 | 58.3 | 50.4 | 34.7 | 25.3 | 21.6 | 22.5 | 21.4 | 23.9 | 23.7 | 21.2 | 21.8 | 24.1 | 24.9 | 27.1 | 29.7 | 33.4 | 49.4 | 51.5 |
|  | |  | | | Min | 0.00 | 0.00 | 0.00 | 0.0820 | 0.249 | 0.533 | 0.781 | 0.932 | 0.988 | 0.899 | 0.908 | 0.864 | 0.914 | 0.879 | 0.730 | 0.532 | 0.425 | 0.305 | 0.220 | 0.0906 | 0.0589 |
|  | |  | | | Max | 0.0701 | 1.21 | 1.81 | 1.65 | 2.19 | 2.96 | 2.71 | 2.40 | 2.60 | 2.49 | 2.49 | 2.60 | 2.52 | 2.40 | 2.34 | 1.98 | 1.67 | 1.23 | 0.947 | 0.564 | 0.422 |

##

## Table S9. Changes of SBP after drug administration under fasting condition

| Time | Index | TGroup | RGroup | T | P |
| --- | --- | --- | --- | --- | --- |
| -1h | N(Missing) | 40(0) | 39(0) | t=0.67 | 0.5049 |
|  | Mean(SD) | 111.38(9.71) | 109.72(12.17) |  |  |
|  | Median | 112 | 109 |  |  |
|  | Q1,Q3 | 103,118.5 | 106,119 |  |  |
|  | Min,Max | 95,133 | 60,126 |  |  |
| 2h | N(Missing) | 40(0) | 39(0) | t=-0.09 | 0.9300 |
|  | Mean(SD) | 110.05(11.26) | 110.28(12.15) |  |  |
|  | Median | 108 | 110 |  |  |
|  | Q1,Q3 | 101.5,118.5 | 100,121 |  |  |
|  | Min,Max | 93,137 | 90,137 |  |  |
| 2h changes | N(Missing) | 40(0) | 39(0) | t=-0.69 | 0.4901 |
|  | Mean(SD) | -1.33(12.78) | 0.56(11.38) |  |  |
|  | Median | -1 | -1 |  |  |
|  | Q1,Q3 | -9,2 | -9,10 |  |  |
|  | Min,Max | -29,28 | -23,30 |  |  |
| 4h | N(Missing) | 40(0) | 39(0) | t=0.08 | 0.9387 |
|  | Mean(SD) | 106.33(11.16) | 106.13(11.51) |  |  |
|  | Median | 105 | 105 |  |  |
|  | Q1,Q3 | 100,114.5 | 97,115 |  |  |
|  | Min,Max | 80,130 | 84,128 |  |  |
| 4h changes | N(Missing) | 40(0) | 39(0) | t=-0.58 | 0.5619 |
|  | Mean(SD) | -5.05(9.74) | -3.59(12.41) |  |  |
|  | Median | -5.5 | -5 |  |  |
|  | Q1,Q3 | -11,-1 | -11,-1 |  |  |
|  | Min,Max | -27,22 | -24,45 |  |  |
| 6h | N(Missing) | 40(0) | 39(0) | t=-0.99 | 0.3250 |
|  | Mean(SD) | 104.65(10.74) | 107.18(11.94) |  |  |
|  | Median | 106.5 | 105 |  |  |
|  | Q1,Q3 | 98,111 | 98,118 |  |  |
|  | Min,Max | 81,127 | 84,131 |  |  |
| 6h changes | N(Missing) | 40(0) | 39(0) | t=-1.65 | 0.1040 |
|  | Mean(SD) | -6.73(9.83) | -2.54(12.64) |  |  |
|  | Median | -8.5 | -5 |  |  |
|  | Q1,Q3 | -12,-0.5 | -10,3 |  |  |
|  | Min,Max | -26,15 | -31,43 |  |  |
| 8h | N(Missing) | 40(0) | 39(0) | t=-0.73 | 0.4667 |
|  | Mean(SD) | 103.9(11.32) | 105.74(11.07) |  |  |
|  | Median | 104.5 | 106 |  |  |
|  | Q1,Q3 | 96,111.5 | 97,113 |  |  |
|  | Min,Max | 84,130 | 86,127 |  |  |
| 8h changes | N(Missing) | 40(0) | 39(0) | t=-1.30 | 0.1990 |
|  | Mean(SD) | -7.48(11.15) | -3.97(12.83) |  |  |
|  | Median | -7 | -2 |  |  |
|  | Q1,Q3 | -14.5,-1.5 | -14,4 |  |  |
|  | Min,Max | -28,19 | -27,38 |  |  |
| 12h | N(Missing) | 40(0) | 39(0) | t=-0.79 | 0.4330 |
|  | Mean(SD) | 110.55(9.99) | 112.46(11.53) |  |  |
|  | Median | 108 | 112 |  |  |
|  | Q1,Q3 | 104.5,117.5 | 104,120 |  |  |
|  | Min,Max | 96,140 | 93,137 |  |  |
| 12h changes | N(Missing) | 40(0) | 39(0) | t=-1.38 | 0.1711 |
|  | Mean(SD) | -0.83(8.52) | 2.74(13.72) |  |  |
|  | Median | -1.5 | 0 |  |  |
|  | Q1,Q3 | -7,4 | -7,11 |  |  |
|  | Min,Max | -17,26 | -18,50 |  |  |
| 24h | N(Missing) | 40(0) | 39(0) | t=-1.39 | 0.1674 |
|  | Mean(SD) | 114.68(9.28) | 117.79(10.59) |  |  |
|  | Median | 113 | 119 |  |  |
|  | Q1,Q3 | 108,123.5 | 110,125 |  |  |
|  | Min,Max | 94,130 | 98,139 |  |  |
| 24h changes | N(Missing) | 40(0) | 39(0) | t=-1.92 | 0.0586 |
|  | Mean(SD) | 3.3(9.25) | 8.08(12.65) |  |  |
|  | Median | 4 | 7 |  |  |
|  | Q1,Q3 | -3,8 | 1,15 |  |  |
|  | Min,Max | -16,27 | -12,56 |  |  |
| 48h | N(Missing) | 40(0) | 39(0) | t=-0.84 | 0.4023 |
|  | Mean(SD) | 120.13(10.96) | 122.1(9.86) |  |  |
|  | Median | 121.5 | 121 |  |  |
|  | Q1,Q3 | 112,127.5 | 116,129 |  |  |
|  | Min,Max | 98,140 | 103,139 |  |  |
| 48h changes | N(Missing) | 40(0) | 39(0) | t=-1.50 | 0.1370 |
|  | Mean(SD) | 8.75(10.41) | 12.38(11.09) |  |  |
|  | Median | 7.5 | 12 |  |  |
|  | Q1,Q3 | 2.5,13.5 | 4,20 |  |  |
|  | Min,Max | -12,44 | -12,48 |  |  |
| 72h | N(Missing) | 40(0) | 39(0) | t=-0.30 | 0.7681 |
|  | Mean(SD) | 117.2(9.65) | 117.87(10.52) |  |  |
|  | Median | 116.5 | 117 |  |  |
|  | Q1,Q3 | 110.5,123.5 | 110,125 |  |  |
|  | Min,Max | 96,138 | 97,139 |  |  |
| 72h changes | N(Missing) | 40(0) | 39(0) | t=-0.94 | 0.3524 |
|  | Mean(SD) | 5.83(10.16) | 8.15(11.91) |  |  |
|  | Median | 8 | 6 |  |  |
|  | Q1,Q3 | -0.5,11 | 0,16 |  |  |
|  | Min,Max | -23,40 | -15,48 |  |  |
| 96h | N(Missing) | 40(0) | 39(0) | t=0.02 | 0.9864 |
|  | Mean(SD) | 119.2(13.19) | 119.15(10.56) |  |  |
|  | Median | 119.5 | 117 |  |  |
|  | Q1,Q3 | 111,127.5 | 112,127 |  |  |
|  | Min,Max | 94,164 | 100,141 |  |  |
| 96h changes | N(Missing) | 40(0) | 39(0) | t=-0.59 | 0.5563 |
|  | Mean(SD) | 7.83(12.23) | 9.44(12) |  |  |
|  | Median | 9 | 7 |  |  |
|  | Q1,Q3 | -1.5,15 | 3,15 |  |  |
|  | Min,Max | -17,43 | -17,55 |  |  |
| 144h | N(Missing) | 40(0) | 39(0) | t=-0.17 | 0.8683 |
|  | Mean(SD) | 105.55(11.25) | 105.92(8.43) |  |  |
|  | Median | 106 | 107 |  |  |
|  | Q1,Q3 | 97,111 | 101,112 |  |  |
|  | Min,Max | 86,132 | 88,125 |  |  |
| 144h changes | N(Missing) | 40(0) | 39(0) | t=-0.77 | 0.4408 |
|  | Mean(SD) | -5.83(10.5) | -3.79(12.71) |  |  |
|  | Median | -8 | -7 |  |  |
|  | Q1,Q3 | -13,5 | -11,4 |  |  |
|  | Min,Max | -25,20 | -30,39 |  |  |
| 168h | N(Missing) | 40(0) | 39(0) | t=-1.10 | 0.2736 |
|  | Mean(SD) | 104.83(10.89) | 107.54(10.98) |  |  |
|  | Median | 102.5 | 107 |  |  |
|  | Q1,Q3 | 96.5,113.5 | 98,115 |  |  |
|  | Min,Max | 90,128 | 85,133 |  |  |
| 168h changes | N(Missing) | 40(0) | 39(0) | t=-1.58 | 0.1184 |
|  | Mean(SD) | -6.55(9.86) | -2.18(14.25) |  |  |
|  | Median | -5.5 | -7 |  |  |
|  | Q1,Q3 | -12,-1 | -11,7 |  |  |
|  | Min,Max | -29,20 | -24,51 |  |  |

## Table S10. Changes of DBP after drug administration under fasting condition

| Time | Index | T Group | R Group | T | P |
| --- | --- | --- | --- | --- | --- |
| -1h | N(Missing) | 40(0) | 39(0) | t=0.14 | 0.8929 |
|  | Mean(SD) | 67.68(7.53) | 67.41(9.77) |  |  |
|  | Median | 67.5 | 67 |  |  |
|  | Q1,Q3 | 63,73 | 63,74 |  |  |
|  | Min,Max | 53,89 | 33,87 |  |  |
| 2h | N(Missing) | 40(0) | 39(0) | t=0.19 | 0.8460 |
|  | Mean(SD) | 62.35(7.96) | 62(8.01) |  |  |
|  | Median | 62 | 60 |  |  |
|  | Q1,Q3 | 56,67.5 | 56,67 |  |  |
|  | Min,Max | 50,87 | 50,87 |  |  |
| 2h changes | N(Missing) | 40(0) | 39(0) | t=0.06 | 0.9554 |
|  | Mean(SD) | -5.33(6.07) | -5.41(7.39) |  |  |
|  | Median | -6.5 | -7 |  |  |
|  | Q1,Q3 | -9,-2 | -9,-2 |  |  |
|  | Min,Max | -16,11 | -16,18 |  |  |
| 4h | N(Missing) | 40(0) | 39(0) | t=-0.49 | 0.6238 |
|  | Mean(SD) | 57.75(7.01) | 58.59(8.12) |  |  |
|  | Median | 57 | 58 |  |  |
|  | Q1,Q3 | 53,62 | 52,63 |  |  |
|  | Min,Max | 47,83 | 47,82 |  |  |
| 4h changes | N(Missing) | 40(0) | 39(0) | t=-0.67 | 0.5080 |
|  | Mean(SD) | -9.93(6.01) | -8.82(8.49) |  |  |
|  | Median | -11 | -10 |  |  |
|  | Q1,Q3 | -14,-5.5 | -14,-5 |  |  |
|  | Min,Max | -23,5 | -24,17 |  |  |
| 6h | N(Missing) | 40(0) | 39(0) | t=-0.30 | 0.7619 |
|  | Mean(SD) | 55.43(7.74) | 55.97(8.32) |  |  |
|  | Median | 55.5 | 56 |  |  |
|  | Q1,Q3 | 50,60 | 51,62 |  |  |
|  | Min,Max | 43,73 | 41,76 |  |  |
| 6h changes | N(Missing) | 40(0) | 39(0) | t=-0.45 | 0.6568 |
|  | Mean(SD) | -12.25(7.3) | -11.44(8.86) |  |  |
|  | Median | -12 | -11 |  |  |
|  | Q1,Q3 | -16.5,-7 | -17,-8 |  |  |
|  | Min,Max | -30,2 | -28,17 |  |  |
| 8h | N(Missing) | 40(0) | 39(0) | t=-0.50 | 0.6209 |
|  | Mean(SD) | 55.53(7.46) | 56.31(6.51) |  |  |
|  | Median | 55.5 | 54 |  |  |
|  | Q1,Q3 | 52,59 | 52,60 |  |  |
|  | Min,Max | 44,78 | 43,73 |  |  |
| 8h changes | N(Missing) | 40(0) | 39(0) | t=-0.56 | 0.5749 |
|  | Mean(SD) | -12.15(7.17) | -11.1(9.25) |  |  |
|  | Median | -12 | -11 |  |  |
|  | Q1,Q3 | -17,-7 | -17,-7 |  |  |
|  | Min,Max | -27,2 | -25,20 |  |  |
| 12h | N(Missing) | 40(0) | 39(0) | t=-0.37 | 0.7142 |
|  | Mean(SD) | 64.6(7.21) | 65.15(6.12) |  |  |
|  | Median | 65 | 65 |  |  |
|  | Q1,Q3 | 58.5,69.5 | 61,69 |  |  |
|  | Min,Max | 52,85 | 53,79 |  |  |
| 12hchanges | N(Missing) | 40(0) | 39(0) | t=-0.45 | 0.6567 |
|  | Mean(SD) | -3.08(6.71) | -2.26(9.34) |  |  |
|  | Median | -2 | -2 |  |  |
|  | Q1,Q3 | -8.5,1 | -8,1 |  |  |
|  | Min,Max | -15,15 | -16,29 |  |  |
| 24h | N(Missing) | 40(0) | 39(0) | t=-0.21 | 0.8373 |
|  | Mean(SD) | 67.7(7.68) | 68.05(7.48) |  |  |
|  | Median | 68.5 | 68 |  |  |
|  | Q1,Q3 | 62,71.5 | 63,73 |  |  |
|  | Min,Max | 53,88 | 56,83 |  |  |
| 24hchanges | N(Missing) | 40(0) | 39(0) | t=-0.32 | 0.7526 |
|  | Mean(SD) | 0.03(6.33) | 0.64(10.42) |  |  |
|  | Median | -0.5 | -2 |  |  |
|  | Q1,Q3 | -4.5,4 | -6,3 |  |  |
|  | Min,Max | -13,14 | -14,41 |  |  |
| 48h | N(Missing) | 40(0) | 39(0) | t=-1.09 | 0.2790 |
|  | Mean(SD) | 71.23(7.62) | 73(6.81) |  |  |
|  | Median | 72 | 73 |  |  |
|  | Q1,Q3 | 64,77 | 68,78 |  |  |
|  | Min,Max | 55,86 | 60,86 |  |  |
| 48hchanges | N(Missing) | 40(0) | 39(0) | t=-1.07 | 0.2858 |
|  | Mean(SD) | 3.55(7.46) | 5.59(9.33) |  |  |
|  | Median | 2.5 | 3 |  |  |
|  | Q1,Q3 | -1,8.5 | 0,9 |  |  |
|  | Min,Max | -21,21 | -7,41 |  |  |
| 72h | N(Missing) | 40(0) | 39(0) | t=-0.84 | 0.4055 |
|  | Mean(SD) | 70.78(6.75) | 72.18(8.12) |  |  |
|  | Median | 72 | 74 |  |  |
|  | Q1,Q3 | 67,76 | 66,78 |  |  |
|  | Min,Max | 54,82 | 53,88 |  |  |
| 72h changes | N(Missing) | 40(0) | 39(0) | t=-0.90 | 0.3699 |
|  | Mean(SD) | 3.1(6.36) | 4.77(9.69) |  |  |
|  | Median | 2.5 | 3 |  |  |
|  | Q1,Q3 | -1.5,8 | 0,9 |  |  |
|  | Min,Max | -10,18 | -8,37 |  |  |
| 96h | N(Missing) | 40(0) | 39(0) | t=1.16 | 0.2500 |
|  | Mean(SD) | 71.95(8.95) | 69.82(7.27) |  |  |
|  | Median | 72.5 | 69 |  |  |
|  | Q1,Q3 | 65.5,78 | 65,74 |  |  |
|  | Min,Max | 54,91 | 53,85 |  |  |
| 96h changes | N(Missing) | 40(0) | 39(0) | t=1.04 | 0.3041 |
|  | Mean(SD) | 4.28(6.39) | 2.41(9.31) |  |  |
|  | Median | 4 | 2 |  |  |
|  | Q1,Q3 | 1,8 | -3,6 |  |  |
|  | Min,Max | -11,23 | -13,35 |  |  |
| 144h | N(Missing) | 40(0) | 39(0) | t=0.00 | 0.9974 |
|  | Mean(SD) | 58.2(7.9) | 58.21(5.97) |  |  |
|  | Median | 57.5 | 59 |  |  |
|  | Q1,Q3 | 53.5,61.5 | 55,63 |  |  |
|  | Min,Max | 43,89 | 44,70 |  |  |
| 144h changes | N(Missing) | 40(0) | 39(0) | t=-0.13 | 0.8945 |
|  | Mean(SD) | -9.48(7.11) | -9.21(10.53) |  |  |
|  | Median | -9 | -10 |  |  |
|  | Q1,Q3 | -12,-6.5 | -16,-4 |  |  |
|  | Min,Max | -23,6 | -25,22 |  |  |
| 168h | N(Missing) | 40(0) | 39(0) | t=0.49 | 0.6277 |
|  | Mean(SD) | 60.8(7.3) | 60(7.31) |  |  |
|  | Median | 59 | 60 |  |  |
|  | Q1,Q3 | 56,63 | 55,64 |  |  |
|  | Min,Max | 50,84 | 44,74 |  |  |
| 168h changes | N(Missing) | 40(0) | 39(0) | t=0.29 | 0.7691 |
|  | Mean(SD) | -6.88(7.08) | -7.41(8.98) |  |  |
|  | Median | -7.5 | -9 |  |  |
|  | Q1,Q3 | -12,-2.5 | -12,-5 |  |  |
|  | Min,Max | -22,7 | -29,22 |  |  |

## Table S11. Changes of SBP after drug administration under fed condition

| Time | Index | T Group | R Group | T | P |
| --- | --- | --- | --- | --- | --- |
| -1h | N(Missing) | 44(0) | 44(0) | t=-0.03 | 0.9734 |
|  | Mean(SD) | 113.93(13.83) | 114.02(11.53) |  |  |
|  | Median | 113 | 114 |  |  |
|  | Q1,Q3 | 105,120.5 | 106,121 |  |  |
|  | Min,Max | 92,170 | 93,139 |  |  |
| 2h | N(Missing) | 44(0) | 44(0) | t=0.34 | 0.7346 |
|  | Mean(SD) | 113.16(13.96) | 112.16(13.63) |  |  |
|  | Median | 111.5 | 110.5 |  |  |
|  | Q1,Q3 | 104.5,117.5 | 104,121.5 |  |  |
|  | Min,Max | 90,165 | 74,136 |  |  |
| 2h changes | N(Missing) | 44(0) | 44(0) | t=0.64 | 0.5261 |
|  | Mean(SD) | -0.77(7.74) | -1.86(8.32) |  |  |
|  | Median | 0 | -2 |  |  |
|  | Q1,Q3 | -5,2.5 | -8,5 |  |  |
|  | Min,Max | -15,21 | -25,14 |  |  |
| 4h | N(Missing) | 44(0) | 44(0) | t=-0.29 | 0.7731 |
|  | Mean(SD) | 108.95(15.63) | 109.86(13.8) |  |  |
|  | Median | 106.5 | 107.5 |  |  |
|  | Q1,Q3 | 98.5,120 | 102,118.5 |  |  |
|  | Min,Max | 84,178 | 83,140 |  |  |
| 4h changes | N(Missing) | 44(0) | 44(0) | t=-0.39 | 0.6960 |
|  | Mean(SD) | -4.98(9.38) | -4.16(10.19) |  |  |
|  | Median | -7 | -5 |  |  |
|  | Q1,Q3 | -11,0.5 | -11,3 |  |  |
|  | Min,Max | -22,24 | -28,23 |  |  |
| 6h | N(Missing) | 44(0) | 44(0) | t=-0.40 | 0.6909 |
|  | Mean(SD) | 108.59(12.59) | 109.7(13.58) |  |  |
|  | Median | 107 | 107.5 |  |  |
|  | Q1,Q3 | 99,115 | 101.5,118.5 |  |  |
|  | Min,Max | 90,148 | 79,137 |  |  |
| 6h changes | N(Missing) | 44(0) | 44(0) | t=-0.55 | 0.5840 |
|  | Mean(SD) | -5.34(8.06) | -4.32(9.35) |  |  |
|  | Median | -5.5 | -5.5 |  |  |
|  | Q1,Q3 | -10,-0.5 | -11.5,2.5 |  |  |
|  | Min,Max | -23,12 | -28,15 |  |  |
| 8h | N(Missing) | 44(0) | 44(0) | t=-0.61 | 0.5426 |
|  | Mean(SD) | 109.27(12.04) | 110.89(12.72) |  |  |
|  | Median | 107 | 109 |  |  |
|  | Q1,Q3 | 102,117 | 103.5,117 |  |  |
|  | Min,Max | 89,148 | 82,142 |  |  |
| 8h changes | N(Missing) | 44(0) | 44(0) | t=-0.71 | 0.4772 |
|  | Mean(SD) | -4.66(9.73) | -3.14(10.27) |  |  |
|  | Median | -5 | -3.5 |  |  |
|  | Q1,Q3 | -11.5,-1 | -12.5,4.5 |  |  |
|  | Min,Max | -23,24 | -26,15 |  |  |
| 12h | N(Missing) | 44(0) | 43(1) | t=0.14 | 0.8907 |
|  | Mean(SD) | 111.3(12.12) | 110.95(10.98) |  |  |
|  | Median | 111.5 | 109 |  |  |
|  | Q1,Q3 | 100.5,121 | 104,117 |  |  |
|  | Min,Max | 90,138 | 90,139 |  |  |
| 12h changes | N(Missing) | 44(0) | 43(1) | t=0.09 | 0.9312 |
|  | Mean(SD) | -2.64(10.76) | -2.81(8.15) |  |  |
|  | Median | -3 | -1 |  |  |
|  | Q1,Q3 | -7,3 | -8,3 |  |  |
|  | Min,Max | -32,32 | -24,14 |  |  |
| 24h | N(Missing) | 43(1) | 43(1) | t=-0.71 | 0.4784 |
|  | Mean(SD) | 114.42(13.02) | 116.35(12.1) |  |  |
|  | Median | 111 | 117 |  |  |
|  | Q1,Q3 | 105,123 | 107,124 |  |  |
|  | Min,Max | 93,150 | 91,145 |  |  |
| 24h changes | N(Missing) | 43(1) | 43(1) | t=-1.01 | 0.3160 |
|  | Mean(SD) | 0.09(11.78) | 2.58(11.08) |  |  |
|  | Median | -1 | 2 |  |  |
|  | Q1,Q3 | -9,6 | -5,10 |  |  |
|  | Min,Max | -28,36 | -26,25 |  |  |
| 48h | N(Missing) | 37(7) | 37(7) | t=-0.17 | 0.8663 |
|  | Mean(SD) | 117.7(11.69) | 118.16(11.7) |  |  |
|  | Median | 120 | 118 |  |  |
|  | Q1,Q3 | 113,126 | 111,128 |  |  |
|  | Min,Max | 90,136 | 93,137 |  |  |
| 48h changes | N(Missing) | 37(7) | 37(7) | t=-0.72 | 0.4757 |
|  | Mean(SD) | 2.49(10.94) | 4.51(13.27) |  |  |
|  | Median | 4 | 6 |  |  |
|  | Q1,Q3 | -2,9 | -3,12 |  |  |
|  | Min,Max | -40,21 | -28,28 |  |  |
| 72h | N(Missing) | 37(7) | 37(7) | t=-1.01 | 0.3140 |
|  | Mean(SD) | 117.95(9.72) | 120.49(11.74) |  |  |
|  | Median | 118 | 122 |  |  |
|  | Q1,Q3 | 111,126 | 115,128 |  |  |
|  | Min,Max | 99,136 | 96,143 |  |  |
| 72h changes | N(Missing) | 37(7) | 37(7) | t=-1.54 | 0.1273 |
|  | Mean(SD) | 2.73(12.02) | 6.84(10.85) |  |  |
|  | Median | 4 | 5 |  |  |
|  | Q1,Q3 | -2,10 | -1,14 |  |  |
|  | Min,Max | -44,23 | -12,40 |  |  |
| 96h | N(Missing) | 37(7) | 37(7) | t=-0.11 | 0.9121 |
|  | Mean(SD) | 119.41(11.84) | 119.68(8.93) |  |  |
|  | Median | 122 | 120 |  |  |
|  | Q1,Q3 | 110,129 | 114,126 |  |  |
|  | Min,Max | 96,140 | 103,136 |  |  |
| 96h changes | N(Missing) | 37(7) | 37(7) | t=-0.68 | 0.5017 |
|  | Mean(SD) | 4.19(11.9) | 6.03(11.51) |  |  |
|  | Median | 8 | 8 |  |  |
|  | Q1,Q3 | -1,12 | 1,12 |  |  |
|  | Min,Max | -39,23 | -25,29 |  |  |
| 144h | N(Missing) | 44(0) | 44(0) | t=-1.49 | 0.1389 |
|  | Mean(SD) | 111.23(11.36) | 115.14(13.13) |  |  |
|  | Median | 111 | 117 |  |  |
|  | Q1,Q3 | 106,118 | 103.5,125 |  |  |
|  | Min,Max | 79,143 | 91,143 |  |  |
| 144h changes | N(Missing) | 44(0) | 44(0) | t=-1.89 | 0.0622 |
|  | Mean(SD) | -2.7(9.1) | 1.11(9.84) |  |  |
|  | Median | -2 | 0.5 |  |  |
|  | Q1,Q3 | -6.5,4 | -6,10 |  |  |
|  | Min,Max | -27,13 | -17,20 |  |  |
| 168h | N(Missing) | 44(0) | 44(0) | t=-0.81 | 0.4182 |
|  | Mean(SD) | 106.89(13.05) | 109.11(12.63) |  |  |
|  | Median | 104 | 108 |  |  |
|  | Q1,Q3 | 97.5,114 | 100,117.5 |  |  |
|  | Min,Max | 82,150 | 90,153 |  |  |
| 168h changes | N(Missing) | 44(0) | 44(0) | t=-1.02 | 0.3085 |
|  | Mean(SD) | -7.05(10.09) | -4.91(9.47) |  |  |
|  | Median | -7.5 | -6 |  |  |
|  | Q1,Q3 | -11,-1.5 | -10.5,1 |  |  |
|  | Min,Max | -40,20 | -27,16 |  |  |

## Table S12. Changes of DBP after drug administration under fed condition

| Time | Index | T Group | R Group | T | P |
| --- | --- | --- | --- | --- | --- |
| -1h | N(Missing) | 44(0) | 44(0) | t=-0.63 | 0.5276 |
|  | Mean(SD) | 68.86(9.01) | 69.98(7.38) |  |  |
|  | Median | 69 | 69 |  |  |
|  | Q1,Q3 | 64,73.5 | 66,74.5 |  |  |
|  | Min,Max | 50,92 | 54,84 |  |  |
| 2h | N(Missing) | 44(0) | 44(0) | t=-0.16 | 0.8715 |
|  | Mean(SD) | 63.86(8.21) | 64.16(8.86) |  |  |
|  | Median | 63 | 64 |  |  |
|  | Q1,Q3 | 58,68.5 | 57.5,69.5 |  |  |
|  | Min,Max | 50,85 | 47,90 |  |  |
| 2h changes | N(Missing) | 44(0) | 44(0) | t=0.55 | 0.5809 |
|  | Mean(SD) | -5(6.71) | -5.82(7.13) |  |  |
|  | Median | -5 | -6.5 |  |  |
|  | Q1,Q3 | -10,-0.5 | -8.5,-2.5 |  |  |
|  | Min,Max | -26,7 | -24,11 |  |  |
| 4h | N(Missing) | 44(0) | 44(0) | t=-0.86 | 0.3908 |
|  | Mean(SD) | 61.98(8.42) | 63.45(7.63) |  |  |
|  | Median | 62 | 64 |  |  |
|  | Q1,Q3 | 56,65 | 58,67 |  |  |
|  | Min,Max | 46,86 | 45,84 |  |  |
| 4h changes | N(Missing) | 44(0) | 44(0) | t=-0.25 | 0.8065 |
|  | Mean(SD) | -6.89(7.4) | -6.52(6.45) |  |  |
|  | Median | -7 | -7 |  |  |
|  | Q1,Q3 | -11,-3 | -10.5,-3 |  |  |
|  | Min,Max | -32,14 | -19,9 |  |  |
| 6h | N(Missing) | 44(0) | 44(0) | t=-1.20 | 0.2347 |
|  | Mean(SD) | 59.09(7.29) | 61(7.67) |  |  |
|  | Median | 57 | 59 |  |  |
|  | Q1,Q3 | 54,62 | 55,64.5 |  |  |
|  | Min,Max | 50,82 | 47,79 |  |  |
| 6h changes | N(Missing) | 44(0) | 44(0) | t=-0.54 | 0.5881 |
|  | Mean(SD) | -9.77(6.69) | -8.98(7.03) |  |  |
|  | Median | -10 | -10 |  |  |
|  | Q1,Q3 | -13,-7 | -14,-3.5 |  |  |
|  | Min,Max | -25,10 | -28,9 |  |  |
| 8h | N(Missing) | 44(0) | 44(0) | t=-0.29 | 0.7709 |
|  | Mean(SD) | 61.02(7.75) | 61.5(7.58) |  |  |
|  | Median | 59 | 61 |  |  |
|  | Q1,Q3 | 55.5,65 | 56.5,65 |  |  |
|  | Min,Max | 48,84 | 47,78 |  |  |
| 8h changes | N(Missing) | 44(0) | 44(0) | t=0.42 | 0.6742 |
|  | Mean(SD) | -7.84(8.26) | -8.48(5.64) |  |  |
|  | Median | -8 | -7.5 |  |  |
|  | Q1,Q3 | -11.5,-2.5 | -10.5,-5 |  |  |
|  | Min,Max | -30,9 | -26,2 |  |  |
| 12h | N(Missing) | 44(0) | 43(1) | t=-0.47 | 0.6375 |
|  | Mean(SD) | 65.61(7.08) | 66.44(9.15) |  |  |
|  | Median | 65 | 66 |  |  |
|  | Q1,Q3 | 60,71.5 | 62,71 |  |  |
|  | Min,Max | 52,78 | 49,92 |  |  |
| 12h changes | N(Missing) | 44(0) | 43(1) | t=0.08 | 0.9362 |
|  | Mean(SD) | -3.25(6.41) | -3.37(7.72) |  |  |
|  | Median | -4 | -3 |  |  |
|  | Q1,Q3 | -7.5,1 | -8,2 |  |  |
|  | Min,Max | -21,14 | -18,13 |  |  |
| 24h | N(Missing) | 43(1) | 43(1) | t=-0.73 | 0.4687 |
|  | Mean(SD) | 67.77(7.42) | 68.98(7.98) |  |  |
|  | Median | 68 | 69 |  |  |
|  | Q1,Q3 | 62,74 | 63,72 |  |  |
|  | Min,Max | 55,83 | 50,91 |  |  |
| 24h changes | N(Missing) | 43(1) | 43(1) | t=-0.24 | 0.8122 |
|  | Mean(SD) | -1.21(7.39) | -0.84(7.08) |  |  |
|  | Median | 0 | -1 |  |  |
|  | Q1,Q3 | -5,4 | -3,4 |  |  |
|  | Min,Max | -26,13 | -20,16 |  |  |
| 48h | N(Missing) | 37(7) | 37(7) | t=-0.23 | 0.8173 |
|  | Mean(SD) | 71.62(7.29) | 72.03(7.74) |  |  |
|  | Median | 71 | 72 |  |  |
|  | Q1,Q3 | 67,76 | 67,79 |  |  |
|  | Min,Max | 58,87 | 58,89 |  |  |
| 48h changes | N(Missing) | 37(7) | 37(7) | t=-0.05 | 0.9631 |
|  | Mean(SD) | 2.43(7.62) | 2.51(7.4) |  |  |
|  | Median | 3 | 3 |  |  |
|  | Q1,Q3 | -2,6 | -3,7 |  |  |
|  | Min,Max | -29,17 | -20,21 |  |  |
| 72h | N(Missing) | 37(7) | 37(7) | t=0.09 | 0.9257 |
|  | Mean(SD) | 72.14(6.8) | 71.97(8.05) |  |  |
|  | Median | 72 | 73 |  |  |
|  | Q1,Q3 | 68,76 | 68,77 |  |  |
|  | Min,Max | 62,90 | 53,84 |  |  |
| 72h changes | N(Missing) | 37(7) | 37(7) | t=0.29 | 0.7726 |
|  | Mean(SD) | 2.95(7.28) | 2.46(7.14) |  |  |
|  | Median | 5 | 4 |  |  |
|  | Q1,Q3 | -1,7 | -3,7 |  |  |
|  | Min,Max | -19,16 | -13,18 |  |  |
| 96h | N(Missing) | 37(7) | 37(7) | t=-1.19 | 0.2392 |
|  | Mean(SD) | 70.81(7.46) | 72.89(7.63) |  |  |
|  | Median | 72 | 73 |  |  |
|  | Q1,Q3 | 66,76 | 68,77 |  |  |
|  | Min,Max | 54,84 | 56,88 |  |  |
| 96h changes | N(Missing) | 37(7) | 37(7) | t=-1.24 | 0.2173 |
|  | Mean(SD) | 1.62(6.67) | 3.38(5.41) |  |  |
|  | Median | 2 | 3 |  |  |
|  | Q1,Q3 | -2,6 | 1,7 |  |  |
|  | Min,Max | -16,15 | -15,12 |  |  |
| 144h | N(Missing) | 44(0) | 44(0) | t=-0.73 | 0.4664 |
|  | Mean(SD) | 61.25(8.35) | 62.66(9.67) |  |  |
|  | Median | 59 | 60 |  |  |
|  | Q1,Q3 | 54.5,66.5 | 56,68.5 |  |  |
|  | Min,Max | 48,88 | 45,85 |  |  |
| 144h changes | N(Missing) | 44(0) | 44(0) | t=-0.18 | 0.8549 |
|  | Mean(SD) | -7.61(7.4) | -7.32(7.71) |  |  |
|  | Median | -7 | -7 |  |  |
|  | Q1,Q3 | -10,-3 | -11.5,-3 |  |  |
|  | Min,Max | -28,6 | -25,17 |  |  |
| 168h | N(Missing) | 44(0) | 44(0) | t=0.36 | 0.7192 |
|  | Mean(SD) | 63.11(8.16) | 62.48(8.38) |  |  |
|  | Median | 62 | 62 |  |  |
|  | Q1,Q3 | 57.5,68 | 56,67 |  |  |
|  | Min,Max | 50,87 | 51,88 |  |  |
| 168h changes | N(Missing) | 44(0) | 44(0) | t=1.05 | 0.2983 |
|  | Mean(SD) | -5.75(7.86) | -7.5(7.83) |  |  |
|  | Median | -5.5 | -8 |  |  |
|  | Q1,Q3 | -10,-3 | -11,-3 |  |  |
|  | Min,Max | -29,24 | -27,11 |  |  |
